# Unsymmetrical Strategy on α-Diimine Nickel and Palladium Mediated Ethylene (Co)Polymerizations

**DOI:** 10.3390/molecules27248942

**Published:** 2022-12-15

**Authors:** Xin Ma, Yixin Zhang, Zhongbao Jian

**Affiliations:** 1 State Key Laboratory of Polymer Physics and Chemistry, Changchun Institute of Applied Chemistry, Chinese Academy of Sciences, Renmin Street 5625, Changchun 130022, China; 2 School of Applied Chemistry and Engineering, University of Science and Technology of China, Hefei 230026, China

**Keywords:** nickel and palladium catalysts, unsymmetrical, ethylene polymerization, polar monomer, polyolefin

## Abstract

Among various catalyst design strategies used in the α-diimine nickel(II) and palladium(II) catalyst systems, the unsymmetrical strategy is an effective and widely utilized method. In this contribution, unsymmetrical nickel and palladium α-diimine catalysts (**Ipty/*^i^*Pr-Ni** and **Ipty/*^i^*Pr-Pd**) derived from the dibenzobarrelene backbone were constructed via the combination of pentiptycenyl and diisopropylphenyl substituents, and investigated toward ethylene (co)polymerization. Both of these catalysts were capable of polymerizing ethylene in a broad temperature range of 0–120 °C, in which **Ipty/*^i^*Pr-Ni** could maintain activity in the level of 10^6^ g mol^−1^ h^−1^ even at 120 °C. The branching densities of polyethylenes generated by both nickel and palladium catalysts could be modulated by the reaction temperature. Compared with symmetrical **Ipty-Ni** and ***^i^*Pr-Ni**, **Ipty/*^i^*Pr-Ni** exhibited the highest activity, the highest polymer molecular weight, and the lowest branching density. In addition, **Ipty/*^i^*Pr-Pd** could produce copolymers of ethylene and methyl acrylate, with the polar monomer incorporating both on the main chain and the terminal of branches. Remarkably, the ratio of the in-chain and end-chain polar monomer incorporations could be modulated by varying the temperature.

## 1. Introduction

Over the past twenty years, late transition metal catalysed olefin polymerization has entered high-speed development, since the benchmark discovery of α-diimine catalysts [1,2,3]. The type of α-diimine catalysts serves as an important component in the late transition metal catalyst field, because of its unique chain walking polymerization pathway that could result in diverse chain structures of polyolefins. The structure of α-diimine catalyst is one of the key factors to produce desired polymers, thus tremendous efforts have been made in designing catalysts [4,5,6,7]. The modification of α-diimine catalysts has been mainly focused on *N*-substituent steric bulk [8,9,10,11,12,13,14,15,16,17,18,19,20], electronic disturbance [21,22,23,24,25], backbone hindrance [26,27,28,29,30], and external stimuli [31,32,33,34].

**Scheme 1:**
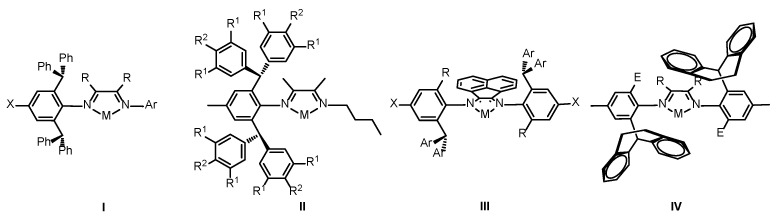
Selected examples of the unsymmetrical strategy on α-diimine nickel and palladium systems (M = Ni or Pd).

Among various catalyst design strategies, the unsymmetrical strategy has been applied extensively. To date, this strategy mainly has three operative manners: (1) different *N*-aryl substituents on the two sides of the α-diimine ligand; (2) different *N*-substituents involving nonaromatic groups on the two sides of the α-diimine ligand; and (3) different substituents on the 2,6-positions of one *N*-aryl ring. In the first manner of the unsymmetrical strategy, dibenzhydryl substituents have been utilized widely as one of the *N*-aryl moiety, coupling with another aryl group to build the α-diimine framework (Scheme 1, **I**). For example, Chen and coworkers reported a series of α-diimine palladium catalysts featuring the dibenzhydryl group and systematically steric demanding aryl rings [35]. These catalysts were capable of modulating activities, polymer molecular weights and branching densities in a wide range. Sun [36,37,38], Dai [39,40,41], and our group [42] also independently developed distinctive unsymmetrical α-diimine nickel and palladium catalysts based on the dibenzhydryl substituent. Besides the dibenzhydryl type, various steric encumbering *N*-aryl substituents have been used in the unsymmetrical systems [43,44,45,46,47]. Recently, a flexible alkyl amine was introduced into the α-diimine ligand, replacing one bulky aryl amine moiety (Scheme 1, **II**) [48]. The resultant unsymmetrical nickel and palladium catalysts exhibited moderate to high activities, and afforded low to high molecular weight polyethylenes with varied branching densities. On the contrast to the former two ways that construct catalysts with unsymmetrical structures, the third method of the unsymmetrical strategy generally endows the catalyst a *C*_2_ symmetrical geometry (Scheme 1, **III**) [49,50,51,52,53,54], among which α-diimine nickel catalysts usually demonstrated excellent ethylene polymerization performance, enabling high activities, high molecular weights, as well as high thermal stability. Moreover, these catalysts also exhibited good catalytic behaviour toward α-olefin polymerization, such as propylene and 1-hexene. Recently, with further combination of the rotation-restricted strategy, benzosuberyl substituents were adopted in the unsymmetrical strategy, forming unsymmetrical structures rather than *C*_2_ symmetrical structures (Scheme 1, **IV**) [55,56]. For example, “sandwich-like” geometries were found in the α-diimine nickel catalysts, which generated polyethylenes with higher molecular weights than those of their free-rotated dibenzhydryl counterparts. 

**Scheme 2:**
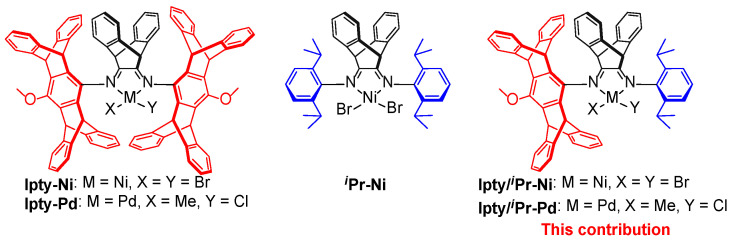
The unsymmetrical strategy on the α-diimine nickel and palladium catalysts in this contribution.

Recently, our group has developed α-diimine nickel and palladium catalysts derived from a bulky dibenzobarrelene backbone and bulky axial pentiptycenyl substituents (Scheme 2, **Ipty-Ni** and **Ipty-Pd**) [10,57,58,59]. These two catalysts could produce ultra-highly branched (>200/1000C) polyethylenes with exclusive methyl branches, of which the branching density generated by the palladium system could be modulated through a polar additive method. Based on these results, we attempted to apply the unsymmetrical strategy in this α-diimine system by replacing one of the pentiptycenyl substituents with the 2,6-diisopropyl-substituted phenyl group, which has been widely used in the catalyst modification [24,26,28,30,34]. Thus, in this contribution, unsymmetrical nickel (**Ipty/*^i^*Pr-Ni**) and palladium (**Ipty/*^i^*Pr-Pd**) α-diimine catalysts were synthesized, and further studied toward ethylene co(polymerization), which exhibited distinctive polymerization properties.

## 2. Results

### 2.1. Synthesis and Characterization of **Ipty/^i^Pr-Ni** and **Ipty/^i^Pr-Pd**

**Scheme 3:**
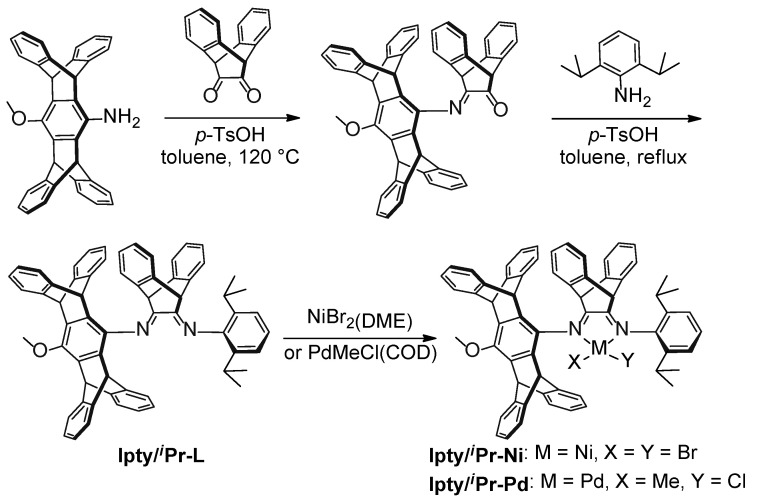
Synthesis of **Ipty/*^i^*Pr-Ni** and **Ipty/*^i^*Pr-Pd**.

As shown in Scheme 3, the unsymmetrical α-diimine ligand **Ipty/*^i^*Pr-L** was synthesized through a two-step procedure, in which the ketone-imine intermediate was first prepared. Treatments of **Ipty/*^i^*Pr-L** with precursors NiBr_2_(DME) or PdMeCl(COD) produced the corresponding nickel dibromide complex **Ipty/*^i^*Pr-Ni** or palladium methyl chloride **Ipty/*^i^*Pr-Pd** in high yields, respectively. For comparison, symmetrical nickel complexes ***^i^*Pr-Ni** [60] and **Ipty-Ni** [10] were also prepared according to the reported literature.

**Figure 1:**
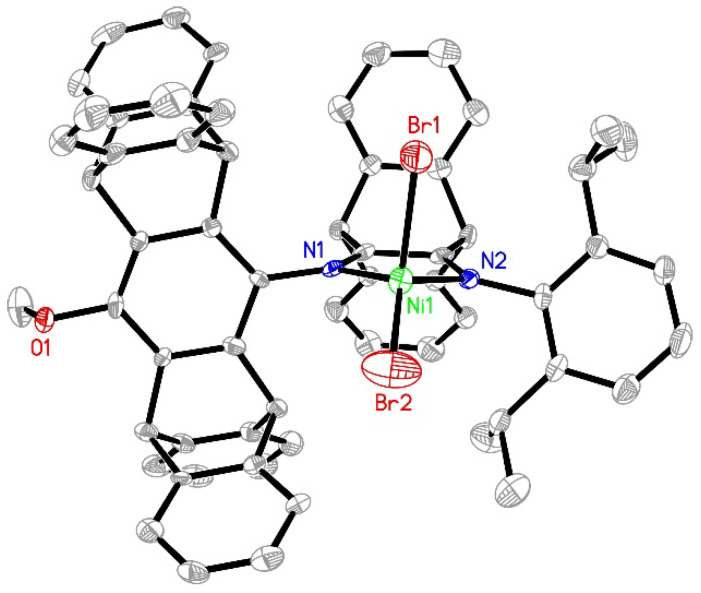
Molecular structure of **Ipty/*^i^*Pr-Ni** drawn with 30% probability ellipsoids. Hydrogen atoms are omitted for clarity. Selected bond lengths (Å): Ni1–Br1 2.360(2), Ni1–Br2 2.273(3), Ni1–N1 2.027(8), Ni1–N2 2.034(9).

**Figure 2:**
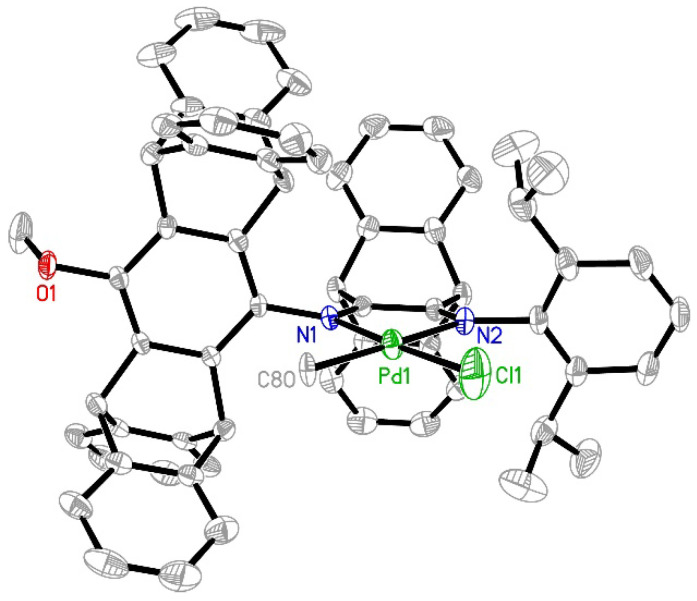
Molecular structure of **Ipty/*^i^*Pr-Pd** drawn with 30% probability ellipsoids. Hydrogen atoms and one CH_2_Cl_2_ molecule are omitted for clarity. Selected bond lengths (Å): Pd1–Cl1 2.280(2), Pd1–C80 2.084(5), Pd1–N1 2.063(4), Pd1–N2 2.151(5).

**Figure 3:**
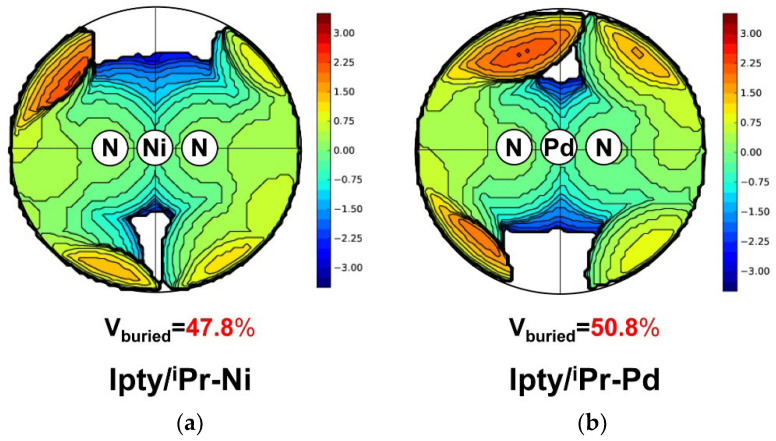
(**a**) Steric maps of **Ipty/*^i^*Pr-Ni** (*V*_bur_ = 47.8%) based on the solid-state structure. (**b**) Steric maps of **Ipty/*^i^*Pr-Pd** (*V*_bur_ = 50.8%) based on the solid-state structure.

The new α-diimine ligand and the palladium complex were fully characterized by ^1^H and ^13^C NMR spectroscopy, and elemental analysis, while the nickel complex was identified by mass spectroscopy and elemental analysis. In addition, molecular structures of **Ipty/*^i^*Pr-Ni** and **Ipty/*^i^*Pr-Pd** were further confirmed by X-ray diffraction analysis (Figure 1 and Figure 2). **Ipty/*^i^*Pr-Ni** displays a typical distorted tetrahedral geometry, in which the angle between the N−Ni−N plane and the Br−Ni−Br plane is 87.43°, smaller than that of **Ipty-Ni** (90.00°) [58]. Moreover, the twist angle between the pentiptycene aryl ring and the nickel diamine (N−Ni−N) plane is 78.77°, smaller than that of 84.25° in **Ipty-Ni**. This result suggests that the repulsive interaction between the dibenzobarrelene backbone and the pentiptycene substituent in **Ipty/*^i^*Pr-Ni** is larger than that in **Ipty-Ni**, which could lead to a more crowded environment around the nickel centre. The larger buried volume *V*_bur_ (47.8%) of **Ipty/*^i^*Pr-Ni** (Figure 3) than that (42.3%) of **Ipty-Ni** further verifies this anticipation. In comparison with ***^i^*Pr-Ni**, the twist angle between the 2,6-diisopropyl phenyl ring and the N−Ni−N plane in **Ipty/*^i^*Pr-Ni** is slightly larger (82.86° vs. 82.45°), indicative of a slightly larger steric hindrance. For the nickel complexes, the order of the buried volume is **Ipty/*^i^*Pr-Ni** (*V*_bur_ = 47.8%) > ***^i^*Pr-Ni** (*V*_bur_ = 45.3%) > **Ipty-Ni** (*V*_bur_ = 42.3%) [60]. For palladium analogues, the steric bulk also follows the order of **Ipty/*^i^*Pr-Pd** > ***^i^*Pr-Pd** > **Ipty-Pd** (*V*_bur_ = 50.8% vs. 49.4% vs. 44.6%). In addition, it is obvious that in the solid-state structures of both **Ipty/*^i^*Pr-Ni** and **Ipty/*^i^*Pr-Pd**, the rigid dibenzobarrelene backbone provides an effective shield on the back side of the metal centre, which could retard the associative chain transfer or chain transfer to monomer [28].

### 2.2. Ethylene Polymerizaiton by **Ipty/^i^Pr-Ni**

The catalytic properties of **Ipty/*^i^*Pr-Ni** were first investigated toward the effect of cocatalysts, wherein three aluminium activators (MMAO, MAO, and AlEt_2_Cl) were selected to carry out ethylene polymerization at 50 °C (Table 1). Among these three aluminium agents, the activation of MMAO endowed **Ipty/*^i^*Pr-Ni** the highest activity of 6.96 × 10^6^ g mol^−1^ h^−1^, along with a high molecular weight that was only slightly lower than the highest value achieved by MAO (1417 kDa vs. 1503 kDa). Moreover, the effect of MMAO and MAO on the branching density was negligible. Compared to MMAO and MAO, Et_2_AlCl had a preference for obtaining higher branching number (86/1000C), yet the polymer molecular weight sharply dropped (Table 1, entries 3 vs. 1, 2).

Taking the activity and the molecular weight into consideration, MMAO was selected as the activator in further studies of **Ipty/*^i^*Pr-Ni** catalytic performance toward ethylene polymerization (Table 2). In a broad temperature range of 0–120 °C, **Ipty/*^i^*Pr-Ni** displayed excellent ethylene polymerization activities in the level of 10^6^–10^7^ g mol^−1^ h^−1^ (Table 2, entries 1–6). With the temperature increasing, the activity ascended first, reaching the highest value of 1.43 × 10^7^ g mol^−1^ h^−1^ at 30 °C that is comparable to that of classical metallocene catalysts, and then descended. We assume that the initiation of **Ipty/*^i^*Pr-Ni** is incomplete at the low temperature of 0 °C, while the catalyst deactivation is attributed to the decrease in activities at higher temperatures. Note that although the activity dropped at 120 °C, it remained in the level of 10^6^ g mol^−1^ h^−1^ (Table 2, entry 6). In terms of the polymer molecular weight, a descending tendency was observed with the temperature increasing. This could be ascribed to the acceleration of chain transfer associated with β-H elimination, which was the outcome of the decreasing axial steric hindrance caused by the faster rotation of the *N*-aryl rings at elevated temperature. Furthermore, the increased β-H elimination by chain transfer could also enhance the probability of chain walking, leading to the increase in the branching density. As a result, the variation of polyethylene microstructures from a lightly branched (16/1000C) semi-crystalline structure (*T*_m_ = 111.9 °C) to a highly branched (105/1000C) amorphous structure was able to be modulated by changing the reaction temperature.

To study the polymerization medium influence on the catalytic and polymer properties, hexane was used under the condition of 50 °C. Compared to those in toluene, the activity and the polymer molecular weight in hexane were enhanced by about 1.63 and 1.43 times, respectively, while the branching density decreased (Table 1, entries 7 vs. 3). It is assumed that the metal cation and the cocatalyst anion interaction is weakened in the hexane [61], rendering the nickel centre more electrophilic [62]. In consequence, ethylene coordination and insertion as the rate-limiting step in α-diimine nickel mediated ethylene polymerization is facilitated to achieve a higher activity. Moreover, the relatively fast ethylene coordination and insertion relative to chain transfer and chain walking could also be responsible for the increase in the molecular weight and the decrease in the branching density.

**Figure 4:**
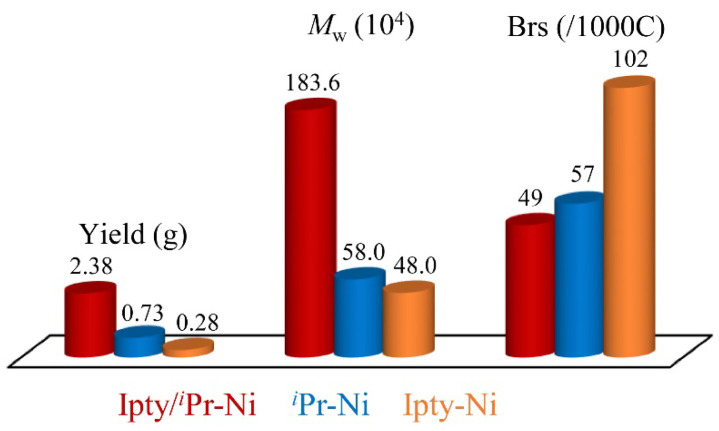
Comparisons of the yield, molecular weight, and branching density of polyethylenes generated by **Ipty/*^i^*Pr-Ni**, ***^i^*Pr-Ni** and **Ipty-Ni** with the activation of MMAO at 30 °C.

Parallel ethylene polymerization experiments of symmetrical ***^i^*Pr-Ni** and **Ipty-Ni** at 30 °C were performed (Table 1, entries 8, 9 vs. 2). Under otherwise identical conditions, unsymmetrical **Ipty/*^i^*Pr-Ni** exhibited the highest activity and polymer molecular weight (Figure 4). Generally, the increase in the steric hindrance around the metal centre is in disadvantage of ethylene coordination, as well as β-H elimination. The former will result in the decrease in the activity, while the latter can improve the polymer molecular weight and lower the branching density. However, as for these three nickel catalysts, the tendency on activity was **Ipty/*^i^*Pr-Ni** > ***^i^*Pr-Ni** > **Ipty-Ni**, in line with the steric bulk order of **Ipty/*^i^*Pr-Ni** > ***^i^*Pr-Ni** > **Ipty-Ni**, which did not fit the general rule. In view of the polymer molecular weight, the trend followed the order of **Ipty/*^i^*Pr-Ni** > ***^i^*Pr-Ni** > **Ipty-Ni**, in agreement with the increasing steric hindrance tendency. Note that the molecular weight of polyethylenes obtained by **Ipty/*^i^*Pr-Ni** was dramatically higher than those of ***^i^*Pr-Ni** and **Ipty-Ni**. Furthermore, the branching density showed an upward trend (**Ipty/*^i^*Pr-Ni** < ***^i^*Pr-Ni** < **Ipty-Ni**), consistent with the decrease in chain walking associated with the reduced β-H elimination.

### 2.3. Copolymerizaiton of Ethylene and Polar Monomer by **Ipty/^i^Pr-Ni**

**Ipty/*^i^*Pr-Ni** was further investigated toward the ethylene copolymerization with the bio-renewable monomer methyl 10-undecenoate (UA) [63] in the activation of MMAO. In the presence of 0.05 M of UA, **Ipty/*^i^*Pr-Ni** showed a high copolymerization activity of 6.3 × 10^5^ g mol^−1^ h^−1^, accompanied with a high molecular weight (443 kDa) and the incorporation ratio of 0.12 mol% (Table 3, entry 1). When the concentration of UA was increased to 0.1 M, the comonomer incorporation was marginally improved to 0.18 mol%, while the activity and the copolymer molecular weight slightly decreased (Table 3, entry 2). To further improve the comonomer incorporation, ethylene pressure was reduced from 8 bar to 6 bar to 2 bar (Table 3, entries 3 and 4). At the low ethylene pressure of 2 bar, the comonomer incorporation reached 0.41 mol%, yet the activity and the copolymer molecular weight sharply dropped, as anticipated.

### 2.4. Mechanical Properties of (Co)Polymers Generatedr by **Ipty/^i^Pr-Ni**

**Figure 5:**
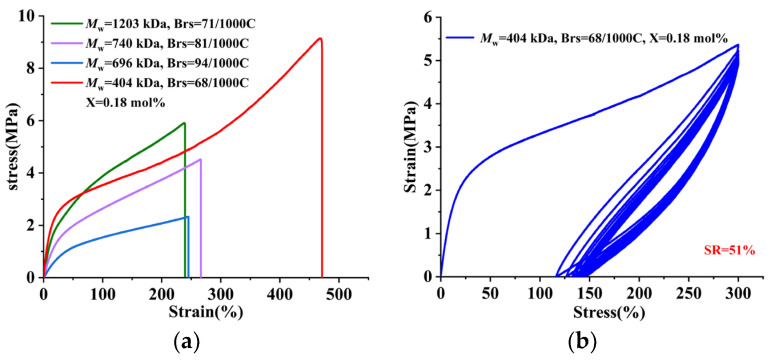
(**a**) Stress–strain curves for selected (co)polymer samples generated by **Ipty/*^i^*Pr-Ni**. (**b**) Plots of hysteresis experiments of ten cycles at a strain of 300% for the copolymer sample generated by **Ipty/*^i^*Pr-Ni**.

Three polyethylene samples with different branching densities were selected for tensile tests to investigate the mechanical properties (Figure 5a). Stress at break values of the three selected polyethylenes followed an opposite tendency (5.9 MPa > 4.5 MPa > 2.3 MPa) to the variation of the branching density (71/1000C < 81/1000C < 94/1000C). Strain at break values were observed in the range of 239% to 266%. For comparison, one copolymer sample with lower branching density was chosen for the mechanical study. Values of stress at break and strain at break were improved dramatically to 9.1 MPa and 470%, respectively. Moreover, the strain recovery of the selected copolymer showed a value of 51%, indicating the feature of elastomer (Figure 5b).

### 2.5. Ethylene Polymerizaiton by **Ipty/^i^Pr-Pd**

In the presence of sodium tetrakis(3,5-bis(trifluoromethyl)phenyl)borate (NaBArF), **Ipty/*^i^*Pr-Pd** could also catalyse ethylene polymerization in the broad temperature range of 0–120 °C (Table 4). Compared to the nickel analogue, the activity and the molecular weight exhibited similar trends with the temperature increasing, of which the activity rose first, and then fell, while the polymer molecular weight decreased. On the contrast, the branching density displayed a decreasing tend along with the elevated temperature. This phenomenon was in agreement with our previous reported **Ipty-Pd** [10], yet inconsistent with the general fact that the branching density in the α-diimine palladium systems is independent with the reaction temperature [64]. According to the previous report by Brookhart, one plausible explanation is that the activation entropy of ethylene insertion into a secondary Pd-alkyl bond is much more negative than that into a primary Pd-alkyl bond [65]. More importantly, highly branched (124–164/1000C) structures of polyethylenes were found, which is rare in the α-diimine palladium catalysts. The relationship of the branching density and ethylene pressure was studied by lowering ethylene pressure (Table 4, entries 7 and 8). Under otherwise identical conditions, the branching density remained almost constant with the variation of ethylene pressure (147–150/1000C). 

### 2.6. Copolymerizaiton of Ethylene and Polar Monomer by **Ipty/^i^Pr-Pd**

The catalytic properties of **Ipty/*^i^*Pr-Pd** toward the copolymerization of ethylene and methyl acrylate (MA) were investigated at various temperatures and comonomer concentrations (Table 5). By employing 1.0 M MA and the radical inhibitor galvinoxyl, the activity went up first, and then fell with the increase in temperature from 30 °C to 90 °C, as well as the copolymer molecular weight (Table 5, entries 1–4). The MA incorporation increased with the elevated temperature, reaching the highest value of 1.98 mol% at 90 °C, while the branching density showed a descending tend, similar to the situation in ethylene polymerization. Under otherwise identical conditions, the variation of comonomer concentration from 0.5 M to 1.0 M to 2.0 M resulted in the increase in MA incorporation (up to 2.21 mol%), yet at the cost of the activity and the copolymer molecular weight, as anticipated (Table 5, entries 3, 5 vs. 6). Moreover, highly branched architectures were formed in the **Ipty/*^i^*Pr-Pd**-catalysed copolymerization of ethylene and MA.

**Figure 6:**
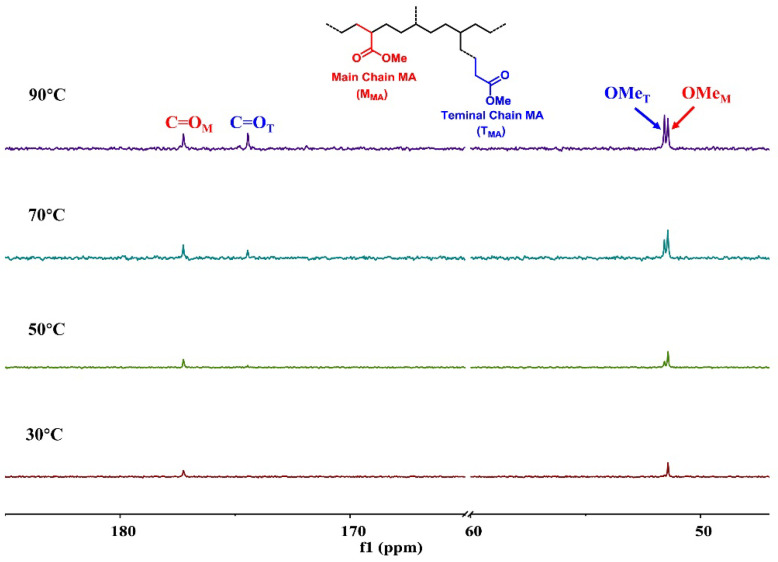
Comparison of C=O and OMe resonances (45–60 ppm and 165–185 ppm) of M_MA_ and T_MA_ at different reaction temperatures (30, 50, 70, and 90 °C; Table 5, entries 1–4) in the ^13^C NMR spectra (500 MHz, 293 K, CDCl_3_) of ethylene-MA copolymers.

Typically, the acrylate groups at the terminal of branches (T_MA_) dominate in the structures of copolymers generated by α-diimine palladium catalysts because of chain walking. **Ipty-Pd** [10] and two other palladium catalysts [15,27] have been reported as rare examples that produced highly branched copolymers featuring very high ratio of acrylate groups on the main chain (M_MA_), which is ascribed to the 1,2-insertion of MA. Compared to **Ipty-Pd**, the highly branched copolymers afforded by **Ipty/*^i^*Pr-Pd** also preferred in-chain acrylate units. As shown in Figure 6, ^13^C NMR spectroscopy revealed characteristic signals for M_MA_ units (177.24 ppm and 51.42 ppm), and T_MA_ units (174.45 ppm and 51.57 ppm) [15,27]. Note that the ratio of M_MA_ and T_MA_ generated by **Ipty/*^i^*Pr-Pd** could be modulated by varying the reaction temperature (Table 5, entries 1–4), in contrast with the slight change in the ratio in the **Ipty-Pd** system. At low temperature of 30 °C, the in-chain incorporation of MA was overwhelming favoured over the end-chain incorporation, with a ratio of 92:8 (Table 5, entry 1). With the temperature increasing, the proportion of the in-chain acrylate incorporation gradually dropped, reaching a similar value to that of the end-chain incorporation at 90 °C (53:47). We speculated that the replacement of one pentiptycenyl substituent with an isopropyl-substituted phenyl group changed the rigid environment of the palladium catalyst, eventually reducing the selectivity of 1,2-insertion. In addition, the comonomer concentration exerted a negligible influence on the comonomer insertion pathway (Table 5, entries 5, 6 vs. 3). 

## 3. Materials and Methods

### 3.1. General Information

All syntheses involving air- and moisture sensitive compounds were carried out using standard Schlenk-type glassware (or in a glove box) under an atmosphere of nitrogen. All solvents were purified from the MBraun SPS system. NMR spectra for the ligand, complexes, and polymers were recorded on a Bruker AV400 (^1^H: 400 MHz, ^13^C: 100 MHz, Bruker, Billerica, MA, USA) or a Bruker AV500 (^1^H: 500 MHz, ^13^C: 125 MHz). The molecular weights (*M*_n_ and *M*_w_) and molecular weight distributions (*M*_w_/*M*_n_) of polyethylenes and copolymers were measured by means of gel permeation chromatography (GPC) on a PL-GPC 220-type high-temperature chromatograph equipped with three PL-gel 10 μm Mixed-B LS type columns at 150 °C. Melting points (*T*_m_) of polyethylenes and copolymers were measured through DSC analyses, which were carried out on a Mettler TOPEM TM DSC Instruments (Mettler, Zurich, Switzerland) under nitrogen atmosphere at heating and cooling rates of 10 °C/min (temperature range: 20–160 °C). Mass spectra of the nickel complex was recorded on an Acquity UPLC & Quattro Premier. Elemental analyses were performed at the National Analytical Research Centre of Changchun Institute of Applied Chemistry. Elemental analysis was performed at the National Analytical Research Centre of Changchun Institute of Applied Chemistry. Stress/strain experiments were performed at 5 mm/min by means of an Electromechanical Universal Testing Machine (E43.104) at room temperature. Polymers were melt-pressed at 150 °C to obtain the test specimens, which have 41-mm gauge length, 17-mm width, and thickness of 1.5 mm. At least three specimens of each polymer were tested.

Materials: 9,10-dihydro-9,10-ethanoanthracene-11,12-dione [66] and pentiptycene aminophenol [67] were prepared using the literature procedures. All other reagents were commercially available from Energy Chemical or Shanghai Titan Scientific Co, Ltd, and used as received.

### 3.2. Synthesis of Ligands and Catalysts

#### 3.2.1. Synthesis of Pentiptycene Aminoanisole

To a solution of pentiptycene aminophenol (3 g, 6.5 mmol) in 100 mL dry DMF at room temperature was added NaH (468 mg, 19.5 mmol) under nitrogen. After the bubbling ceased, CH_3_I (0.6 mL, 9.75 mmol) was added, and the mixture was stirred for 24 h. The reaction was quenched by adding 400 mL of distilled water, and then the aqueous layer was extracted with 3 × 50 mL of CH_2_Cl_2_. The combined organic phase was washed with 3 × 50 mL of distilled water and 50 mL of brine, and then was separated and dried with sodium sulphate. After evaporation of the solvent, the pure product was obtained as a white solid (2.6 g, 84.1% yield). ^1^H NMR (500 MHz, 298 K, CDCl_3_, 7.26 ppm): δ = 7.27–7.36 (m, 8H, aryl-*H*), 6.98–6.88 (m, 8H, aryl-*H*), 5.67 (s, 2H, C*H*Ph_2_), 5.39 (s, 2H, C*H*Ph_2_), 3.85 (s, 3H, OC*H*_3_).

#### 3.2.2. Synthesis of Ligand **Ipty/*^i^*Pr-L**

A solution of pentiptycene aminoanisole (1.12 g, 2.35 mmol), 9,10-dihydro-9,10-ethanoanthracene-11,12-dione (0.5 g, 2.13 mmol) and *p*-toluenesulfonic acid (20 mg) in toluene (200 mL) was stirred at 120 °C for 24 h. The solvent was evaporated under reduced pressure and purified by column chromatography (hexane/CH_2_Cl_2_, 1:1) to afford the ketone-imine compound as an orange solid (0.9 g, 61% yield), which was used in next step without any further purification.

A solution of the ketone-imine compound (0.8 g, 1.15 mmol), 2,6-diisopropylaniline (0.336 g, 1.9 mmol) and *p*-toluenesulfonic acid (20 mg) in toluene (80 mL) was refluxed for 24 h, the solvent was evaporated under reduced pressure and purified by column chromatography (hexane/CH_2_Cl_2_, 1:1) to afford the targeted ligand as an orange solid (0.65g, 70% yield). ^1^H NMR (500 MHz, 298 K, CDCl_3_, 7.26 ppm): δ = 7.42 (d, 2H, aryl-*H*), 7.36–7.17 (m, 11H, aryl-*H*), 7.06–6.96 (m, 6H, aryl-*H*), 6.85–6.75 (m, 8H, aryl-*H*), 5.78 (s, 2H, C*H*), 5.13 (s, 1H, C*H*), 4.91 (s, 2H, C*H*), 4.78 (s, 1H, C*H*), 4.03 (s, 3H, OC*H*_3_), 2.81 (m, 2H, C*H*), 1.34 (dd, 6H, CHC*H*_3_), 1.29 (dd, 6H, CHC*H*_3_). ^13^C{^1^H} NMR (125 MHz, 298 K, CDCl_3_, 77.16 ppm): *δ* = 161.75, 159.40 (N=*C*-Me), 147.12, 145.98, 145.76, 145.62, 145.54, 144.56, 139.25, 138.52, 137.56, 136.41, 135.89, 132.14, 127.62, 127.18, 125.64, 125.42, 125.31, 124.92, 124.85, 124.57, 124.33, 123.51, 123.23, 63.19 (O*C*H_3_), 51.73, 51.48, 49.46, 48.38, 28.78, 27.06, 23.68, 22.86. Elemental analysis: Anal. Calcd for C_63_H_50_N_2_O: C, 88.91; H, 5.92; N, 3.29. Found: C, 88.82; H, 5.83; N, 3.32.

#### 3.2.3. Synthesis of the Nickel Catalyst **Ipty/*^i^*Pr-Ni**

A mixture of **Ipty/*^i^*Pr-L** (200.0 mg, 0.23 mmol) and (DME)NiBr_2_ (72.5 mg, 0.23 mmol) (DME = 1,2-dimethoxyethane) were stirred in 50 mL of dichloromethane for 1 day at room temperature. The solvent was evaporated under reduced pressure, and the desired compound can be isolated from repeated recrystallized from *n*-hexane and dichloromethane. The pure compound was obtained as a dark-yellow solid (180 mg, 73% yield). MALDI-TOF-MS (*m*/*z*): 851.8 [M-Ni-2Br]^+^. Elemental analysis: Anal. Calcd for C_63_H_50_Br_2_N_2_NiO: C, 70.74; H, 4.71; N, 2.62. Found: C, 70.56; H, 4.80; N, 2.56.

#### 3.2.4. Synthesis of the Nickel Catalyst **Ipty/*^i^*Pr-Pd**

To a solution of **Ipty/*^i^*Pr-L** (250 mg, 0.294 mmol) in dry dichloromethane (25 mL) was added 81.8 mg (0.308 mmol) of PdMeCl(COD). After stirring the mixture for 2 days at room temperature, the solvent was evaporated under reduced pressure. The desired compound can be isolated from repeated recrystallized from *n*-hexane and dichloromethane, and was obtained as an orange solid (220 mg, 74.2% yield). ^1^H NMR (500 MHz, 298 K, CDCl_3_, 7.26 ppm): *δ* = 7.46–7.41 (m, 6H, aryl-*H*), 7.35–7.20 (m, 9H, aryl-*H*), 7.07 (t, 2H, aryl-*H*), 6.98 (t, 2H, aryl-*H*), 6.84 (m, 4H, aryl-*H*), 6.76–6.71 (m, 4H, aryl-*H*), 5.80 (s, 2H, C*H*), 5.18 (s, 2H, C*H*), 5.09 (s, 1H, C*H*), 4.74 (s, 1H, C*H*), 4.05 (s, 3H, OC*H*_3_), 3.15 (m, 2H, C*H*), 1.63 (d, 6H, C*H*_3_), 1.33 (d, 6H, C*H*_3_), -0.20 (s, 3H, Pd-C*H*_3_). ^13^C{^1^H} NMR (125 MHz, 298 K, CDCl_3_, 77.16 ppm): *δ* = 174.11 (N=*C*-Me), 167.60 (N=*C*-Me), 149.26, 145.30, 144.98, 144.89, 143.50, 140.43, 139.25, 138.28, 137.20, 136.75, 135.90, 132.53, 128.66, 127.69, 125.89, 125.86, 125.76, 125.67, 125.42, 124.97, 124.89, 123.77, 123.60, 123.30, 63.13 (O*C*H_3_), 51.38, 50.62, 49.56, 48.39, 31.74, 31.20 29.30, 27.81 24.52, 23.60, 22.80, 14.26, 4.04 (Pd-*C*H_3_). Elemental analysis: Anal. Calcd for C_64_H_53_ClN_2_OPd: C, 76.26; H, 5.30; N, 2.78. Found: C, 76.37; H, 5.25; N, 2.84.

### 3.3. X-ray Diffraction

All data collections were performed at 293 K on a Bruker SMART APEX diffractometer with a CCD area detector, using graphite-monochromated Mo Kα radiation (λ = 0.71073 Å) or Cu Kα radiation (λ = 1.54178 Å). The determination of crystal class and unit cell parameters was carried out by the SMART program package [68]. The raw frame data were processed using SAINT and SADABS to yield the reflection data file [69]. All structures were solved by direct methods and refined by full-matrix least-squares procedures on *F*^2^ using SHELXTL or Olex2 [70,71]. Refinement was performed on *F*^2^ anisotropically for all non-hydrogen atoms by the full-matrix least-squares method. The hydrogen atoms were placed at the calculated positions and were included in the structure calculation without further refinement of the parameters. Exceptions and special features: For **Ipty/*^i^*Pr-Ni**, the BASF/TWIN refinement was performed to obtain the reasonable Flack x. The program SQUEEZE [72] was used to remove mathematically the effect of the solvent. The quoted formula and derived parameters are not included the squeezed solvent molecules. For **Ipty/*^i^*Pr-Pd**, the BASF/TWIN refinement was performed to obtain the reasonable Flack x. The program SQUEEZE [72] was used to remove mathematically the effect of the solvent. The quoted formula and derived parameters are not included the squeezed solvent molecules.

### 3.4. General Procedures for the Polymerizations

#### 3.4.1. A General Procedure for Ethylene Polymerization

In a typical experiment, a 350 mL glass pressure reactor connected with a high-pressure gas line was firstly dried at 90 °C under vacuum for at least 1 h. The reactor was then adjusted to the desired polymerization temperature. 98 mL of toluene and the cocatalyst were added to the reactor under N_2_ atmosphere, and then the desired amount of the catalyst in 2 mL of CH_2_Cl_2_ was injected into the polymerization system via syringe. With a rapid stirring, the reactor was pressurized and maintained at the desired ethylene pressure. After the desired reaction time, the pressure reactor was vented, and the polymerization was quenched via the addition of 100 mL EtOH (for the nickel system: acidic EtOH containing 5% HCl), filtered and dried in a vacuum oven to the constant weight.

#### 3.4.2. A General Procedure for the Copolymerization of Ethylene with Polar Monomer

In a typical experiment, a 150 mL glass pressure reactor connected with a high-pressure gas line was firstly dried at 90 °C under vacuum for at least 1 h. The reactor was then adjusted to the desired polymerization temperature. 20 mL of toluene with the cocatalyst and polar monomer was added to the reactor under N_2_ atmosphere, then the desired amount of the catalyst in 2 mL of CH_2_Cl_2_ was injected into the polymerization system via syringe subsequently. With a rapid stirring, the reactor was pressurized and maintained at desired ethylene pressure. After the desired reaction time, the pressure reactor was vented, and the polymerization was quenched via the addition of 20 mL EtOH (for the nickel system: acidic EtOH containing 5% HCl), filtered and dried in a vacuum oven to the constant weight.

## 4. Conclusions

With the combination of the pentiptycenyl and diisopropylphenyl substituents, the unsymmetrical α-diimine nickel and palladium complexes (**Ipty/*^i^*Pr-Ni** and **Ipty/*^i^*Pr-Pd**) were constructed. In the activation of MMAO, **Ipty/*^i^*Pr-Ni** exhibited excellent activities toward ethylene polymerization in a broad temperature range of 0–120 °C. Remarkably, the activity in the level of 10^6^ g mol^−1^ h^−1^ was accessible even at high temperature of 120 °C. High molecular weight polyethylenes with variable branching density were accessible by varying the reaction temperature. Therefore, polyethylenes could convert from the lightly branched semi-crystalline structure to the highly branched amorphous structure. Compared to symmetrical **Ipty-Ni** and ***^i^*Pr-Ni**, unsymmetrical **Ipty/*^i^*Pr-Ni** displayed much higher ethylene polymerization activity and molecular weight of polyethylenes, yet with the lowest branching density. Moreover, **Ipty/*^i^*Pr-Ni** could copolymerize ethylene and methyl 10-undecenoate. As for **Ipty/*^i^*Pr-Pd**, the broad temperature range of 0–120 °C was also applicable for ethylene polymerization. Polyethylenes with ultrahigh branching densities were obtained, and more importantly, the branching density could be modulated through changing the reaction temperature. In the copolymerization of ethylene and methyl acrylate, highly branched copolymers were afforded, with the acrylate units locating on both the main chain and the terminal of branches. In-chain acrylate incorporation dominated at low temperature of 30 °C, and the ratio of in-chain and end-chain incorporations could be modulated by the reaction temperature. This work shows an unsymmetrical strategy as an effective tool for modulating olefin polymerization.

## Figures and Tables

**Table 1:** Effect of cocatalysts on ethylene polymerization catalysed by **Ipty/*^i^*Pr-Ni ^a^**.

Entry	Co-Cat.	Yield (g)	Act. (10^6^) ^b^	*M*_n_ (10^4^) ^c^	*M*_w_ (10^4^) ^c^	*M*_w_/*M*_n_ ^c^	Brs ^d^	*T*_m_ (°C) ^e^
1	MMAO	2.32	6.96	81.2	141.7	1.7	65	54.6
2	MAO	1.62	4.86	92.0	150.3	1.6	64	60.2
3	Et_2_AlCl	1.87	5.61	48.1	88.3	1.8	86	35.3

^a^ Reaction conditions: Ni catalyst (1.0 μmol), cocatalyst (500 equiv.), toluene/CH_2_Cl_2_ (98 mL/2 mL), ethylene (8 bar), 50 °C, 20 min, 750 rpm, at least two runs, unless noted otherwise. ^b^ Activity is in unit of g mol^−1^ h^−1^. ^c^ Determined by GPC in 1,2,4-trichlorobenzene at 150 °C using a light scattering detector. ^d^ Number of branches per 1000C, as determined by ^1^H NMR (see supplementary materials) in C_2_D_2_Cl_4_ at 110 °C. ^e^ Determined by DSC (second heating).

**Table 2:** Ethylene polymerization catalysed by **Ipty/*^i^*Pr-Ni ^a^**.

Entry	*T* (°C)	Yield (g)	Act. (10^6^) ^b^	*M*_n_ (10^4^) ^c^	*M*_w_ (10^4^) ^c^	*M*_w_/*M*_n_ ^c^	Brs ^d^	*T*_m_ (°C) ^e^
1	0	2.32	13.92	166.3	231.4	1.4	16	111.9
2	30	2.38	14.28	111.3	183.6	1.6	49	78.0
3	50	1.96	11.76	65.6	120.3	1.8	71	58.0
4	70	1.87	11.22	40.4	74.0	1.8	81	45.9
5	90	1.18	7.08	36.8	69.6	1.9	94	-
6 ^f^	120	0.57	1.37	10.4	20.6	2.0	105	-
7 ^g^	50	3.20	19.20	96.2	173.1	1.8	62	71.7
8 ^h^	30	0.73	4.38	33.5	58.0	1.7	57	39.3
9 ^i^	30	0.28	1.68	29.8	48.0	1.6	102	118.7

^a^ Reaction conditions: Ni catalyst (1.0 μmol), MMAO (500 equiv.), toluene/CH_2_Cl_2_ (98 mL/2 mL), ethylene (8 bar), 10 min, 750 rpm, at least two runs, unless noted otherwise. ^b^ Activity is in unit of g mol^−1^ h^−1^. ^c^ Determined by GPC in 1,2,4-trichlorobenzene at 150 °C using a light scattering detector. ^d^ Number of branches per 1000C, as determined by ^1^H NMR in C_2_D_2_Cl_4_ at 110 °C. ^e^ Determined by DSC (second heating). ^f^ 25 min. ^g^ hexane. ^h^ ***^i^*Pr-Ni**. ^i^ **Ipty-Ni**.

**Table 3:** Copolymerization of ethylene and UA catalysed by **Ipty/*^i^*Pr-Ni ^a^**.

Entry	p (bar)	Yield (g)	Act. (10^6^) ^b^	*X* (mol%) ^c^	*M*_n_ (10^4^) ^d^	*M*_w_ (10^4^) ^d^	*M*_w_/*M*_n_ ^d^	Brs ^c^	*T*_m_ (°C) ^e^
1 ^f^	8	1.57	0.63	0.12	25.7	44.3	1.7	85	-
2	8	1.55	0.62	0.18	22.0	40.4	1.8	68	55.2
3	6	1.16	0.46	0.21	22.2	40.2	1.8	71	54.2
4	2	0.19	0.08	0.41	7.6	15.0	2.0	81	-

^a^ Reaction conditions: Ni catalyst (5.0 μmol), MMAO (500 equiv.), UA (0.1 mol/L), toluene/CH_2_Cl_2_ (23 mL/2 mL), 50 °C, 30 min, 750 rpm, at least two runs, unless noted otherwise. ^b^ Activity is in unit of g mol^−1^ h^−1^. ^c^ *X* = Incorporation of polar monomer, Brs = Number of branches per 1000C, as determined by ^1^H NMR in C_2_D_2_Cl_4_ at 110 °C. ^d^ Determined by GPC in 1,2,4-trichlorobenzene at 150 °C using a light scattering detector. ^e^ Determined by DSC (second heating). ^f^ UA (0.05 mol/L).

**Table 4:** Ethylene polymerization catalysed by **Ipty/*^i^*Pr-Pd ^a^**.

Entry	*T* (°C)	P (bar)	Yield (g)	Act. (10^6^) ^b^	*M*_n_ (10^4^) ^c^	*M*_w_ (10^4^) ^c^	*M*_w_/*M*_n_ ^c^	Brs ^d^
1 ^e^	0	8	1.38	0.06	10.5	15.5	1.5	164
2	30	8	0.55	0.37	9.7	15.2	1.6	156
3	50	8	1.86	1.24	7.1	16.2	2.3	147
4	70	8	1.15	0.77	3.7	7.7	2.1	139
5	90	8	0.28	0.19	2.2	4.6	2.1	133
6	120	8	0.10	0.07	1.1	2.2	2.0	124
7	50	4	1.63	1.08	8.0	16.4	2.0	150
8	50	2	1.01	0.67	5.2	11.1	2.1	149

^a^ Reaction conditions: Pd catalyst (3.0 μmol), NaBArF (1.5 equiv.), toluene/CH_2_Cl_2_ (98 mL/2 mL), 30 min, 750 rpm, at least two runs, unless noted otherwise. ^b^ Activity is in unit of g mol^−1^ h^−1^. ^c^ Determined by GPC in 1,2,4-trichlorobenzene at 150 °C using a light scattering detector. ^d^ Number of branches per 1000C, as determined by ^1^H NMR in CDCl_3_ at 25 °C. ^e^ 8 h.

**Table 5:** Copolymerization of ethylene and MA catalysed by **Ipty/*^i^*Pr-Pd ^a^**.

Entry	*T* (°C)	Yield (g)	Act. (10^4^) ^b^	*X* (mol%) ^c^	*M*_n_ (10^4^) ^d^	*M*_w_ (10^4^) ^d^	*M*_w_/*M*_n_ ^d^	Brs ^c^	M_MA_:T_MA_ ^e^
1	30	0.43	0.54	0.50	1.6	3.6	2.3	154	92:8
2	50	0.71	0.89	0.77	4.0	6.6	1.6	145	73:27
3	70	0.87	1.09	1.25	3.9	7.4	1.9	138	62:38
4	90	0.33	0.42	1.98	1.8	3.8	2.1	128	53:47
5 ^f^	70	0.90	1.12	0.71	4.7	9.3	2.0	143	64:36
6 ^g^	70	0.38	0.48	2.21	2.5	4.6	1.9	137	60:40

^a^ Reaction conditions: Pd catalyst (10.0 μmol), NaBArF (1.5 equiv.), galvinoxyl (5.0 equiv), MA (1.0 mol/L), toluene/CH_2_Cl_2_ (23 mL/2 mL), ethylene (8 bar), 8 h, 750 rpm, at least two runs, unless noted otherwise. ^b^ Activity is in unit of g mol^−1^ h^−1^. ^c^ X = Incorporation of polar monomer, Brs = Number of branches per 1000C, as determined by ^1^H NMR in CDCl_3_ at 25 °C. ^d^ Determined by GPC in 1,2,4-trichlorobenzene at 150 °C using a light scattering detector. ^e^ The ratio of MA incorporated into the main chain of the copolymer and MA incorporated at the terminal of branches of the copolymer (M_MA_:T_MA_), as determined by ^13^C NMR in CDCl_3_ at 25 °C. ^f^ MA (0.5 mol/L). ^g^ MA (2.0 mol/L).

## Data Availability

The data presented in this study are available in this article.

## Supplementary Materials

The following supporting information can be downloaded at: https://www.mdpi.com/article/10.3390/molecules27248942/s1, Figures S1–S5: Characterization of the ligand and complexes; Figures S6–S44: NMR figures of (co)polymers; Figures S45–S67: GPC figures of (co)polymers; Figures S68–S79: DSC figures of (co)polymers; Table S1: Crystallographic data for **Ipty/*^i^*Pr-Ni** and **Ipty/*^i^*Pr-Pd**.

## Sample Availability

Samples of the compounds **Ipty/*^i^*Pr-Ni** and **Ipty/*^i^*Pr-Pd** are available from the authors.
