# Supplementary material for: Unsymmetrical Strategy on α-Diimine Nickel and Palladium Mediated Ethylene (Co)Polymerizations
Source: Molecules. 2022 Dec 15;27(24):8942. doi: 10.3390/molecules27248942 (PMC9785926; doi:10.3390/molecules27248942)
Supplement: Supplementary file 1 [file molecules-27-08942-s001.zip › molecules-2081417-Supplementary.pdf]

Supplementary Information

Unsymmetrical Strategy on α-Diimine Nickel and Palladium Mediated Ethylene (Co)Polymerizations

Xin Ma,[a,b] Yixin Zhang,[a,*] Zhongbao Jian[a,b*]

[a] State Key Laboratory of Polymer Physics and Chemistry, Changchun Institute of Applied Chemistry, Chinese Academy of Sciences, Renmin Street 5625, Changchun 130022
[b] University of Science and Technology of China, Hefei 230026

Conents

1. Characterization of the ligand and complexes 2
2. NMR figures of (co)polymers 4
3. GPC figures of (co)polymers 23
4. DSC figures of (co)polymers 31
5. Crystallographic data 35

1. Characterization of the ligand and complexes

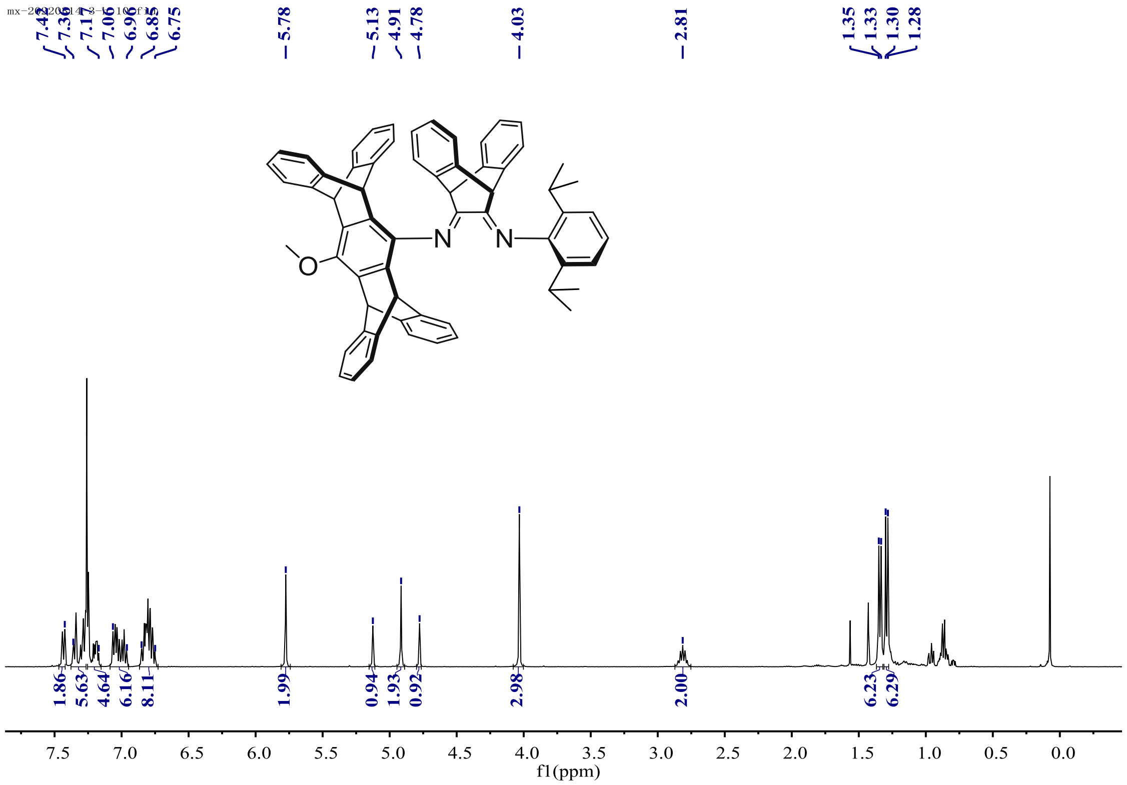

Figure S1. ^{1}H NMR spectrum of **Ipty/^{i}Pr-L** in $CDCl_3$.

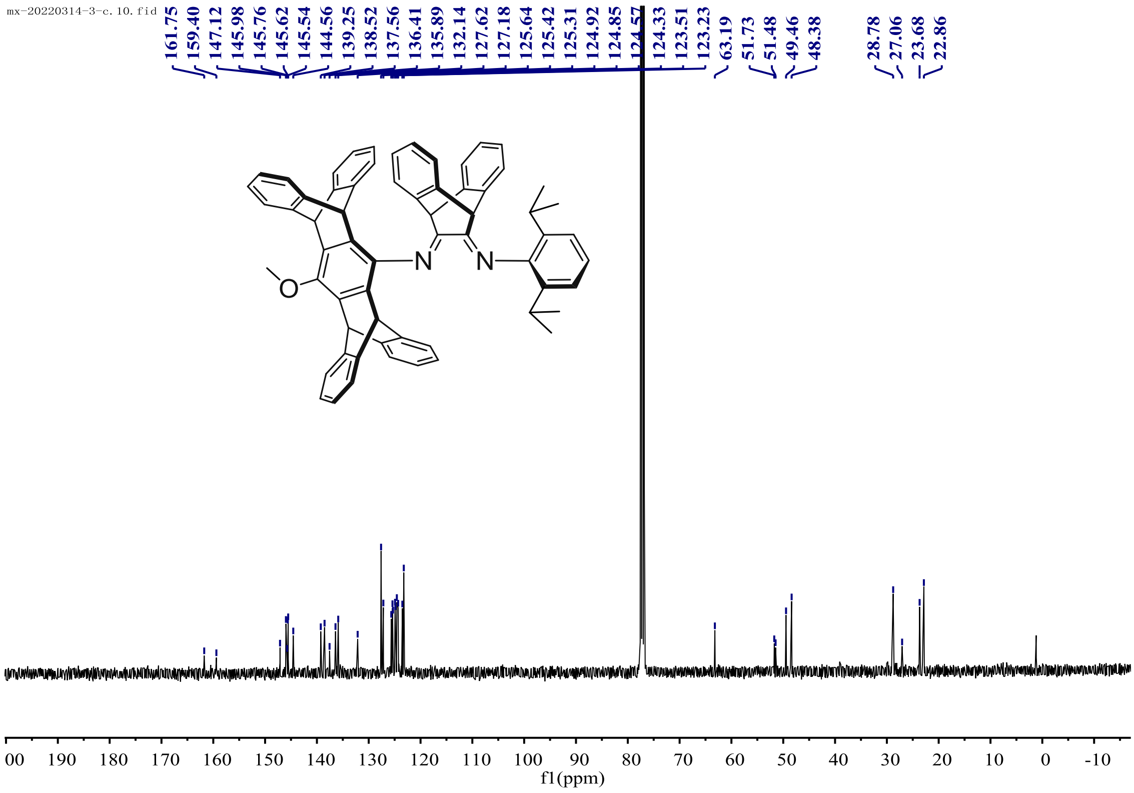

Figure S2. ^{13}C NMR spectrum of **Ipty/^{i}Pr-L** in $CDCl_3$.

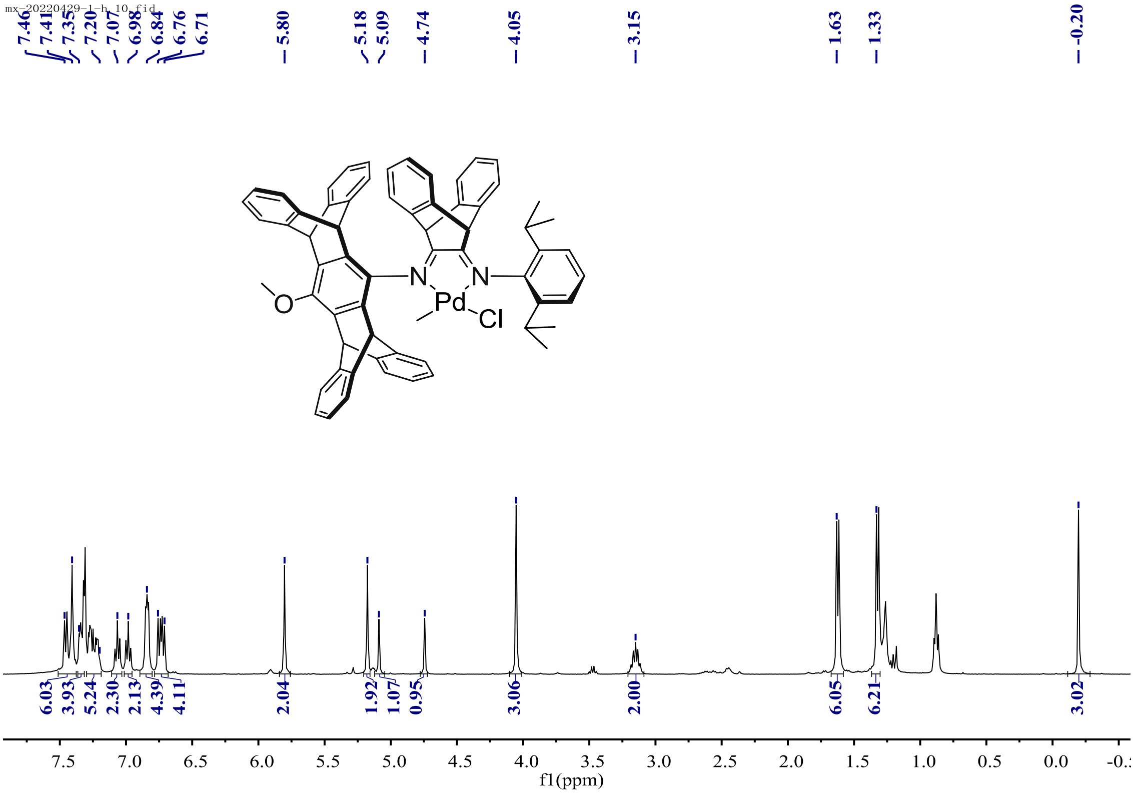

Figure S3. ^{1}H NMR spectrum of **Ipty/^{i}Pr-Pd** in $CDCl_3$.

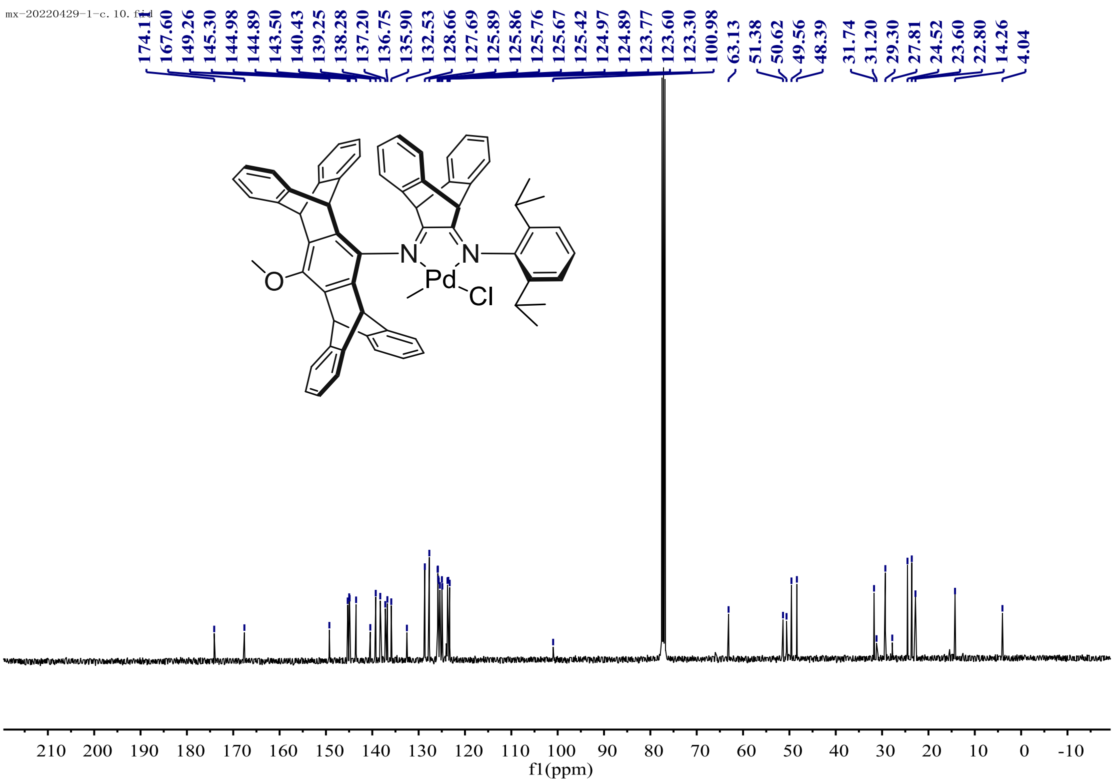

Figure S4. ^{13}C NMR spectrum of **Ipty/^{i}Pr-Pd** in $CDCl_3$.

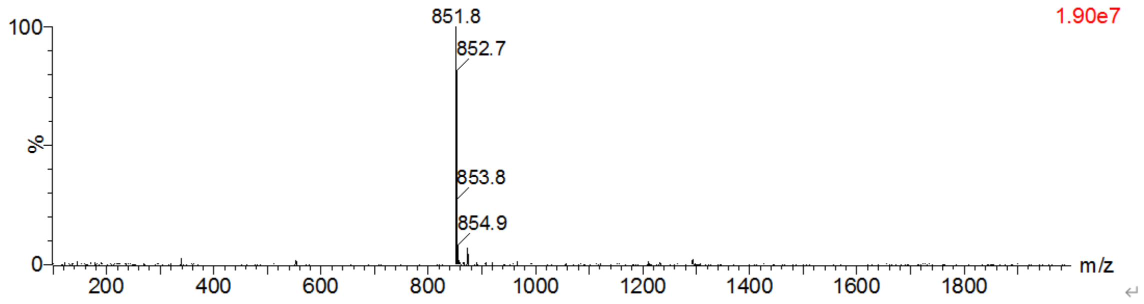

Figure S5. MALDI-TOF-MS of **Ipty/^{i}Pr-Ni**.

2. NMR figures of (co)polymers

$$\text{Me groups / 1000C} = \frac{2\times I_{Me}}{3\times I_{tot}} * 1000 = \frac{2\times 3}{3\times(27.68+3)} * 1000 = 65.2$$

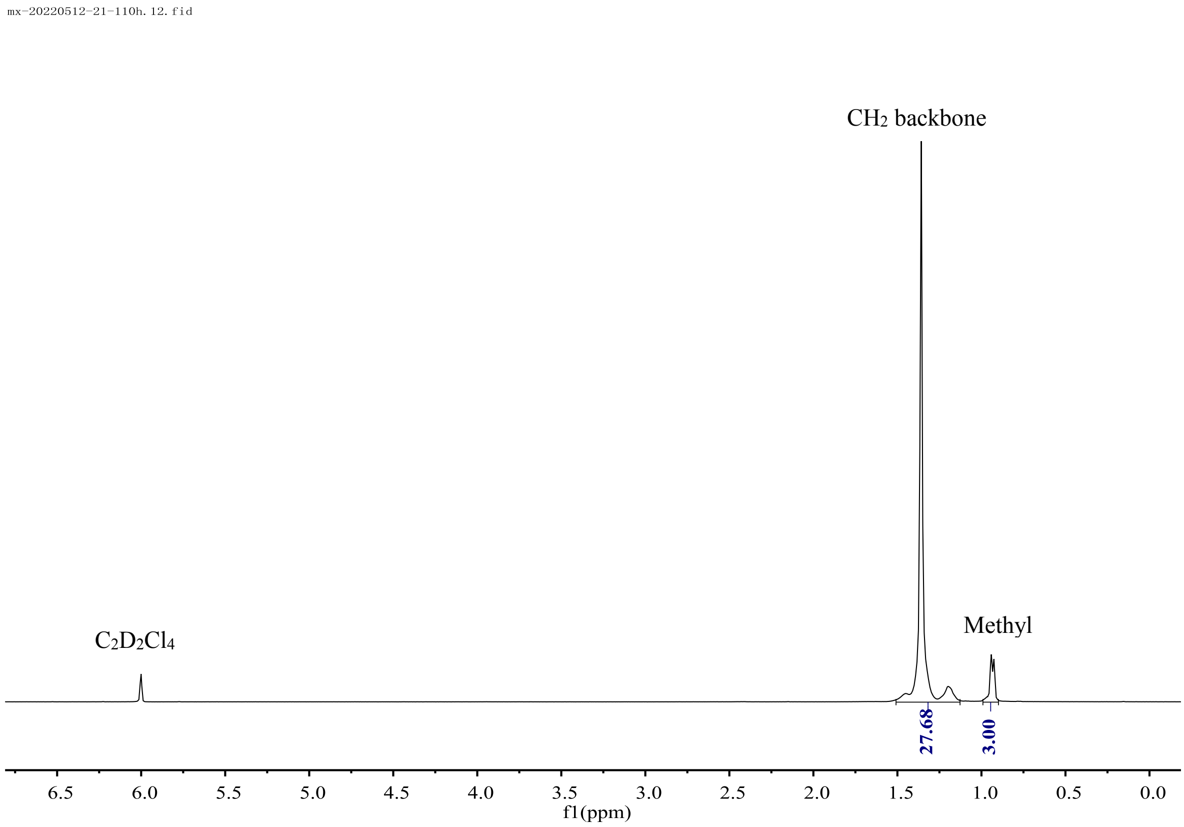

Figure S6. ^{1}H NMR spectrum (400 MHz, $C_2D_2Cl_4$, 110 °C) of polyethylene generated by **Ipty/^{i}Pr-Ni** from table 1, entry 1.

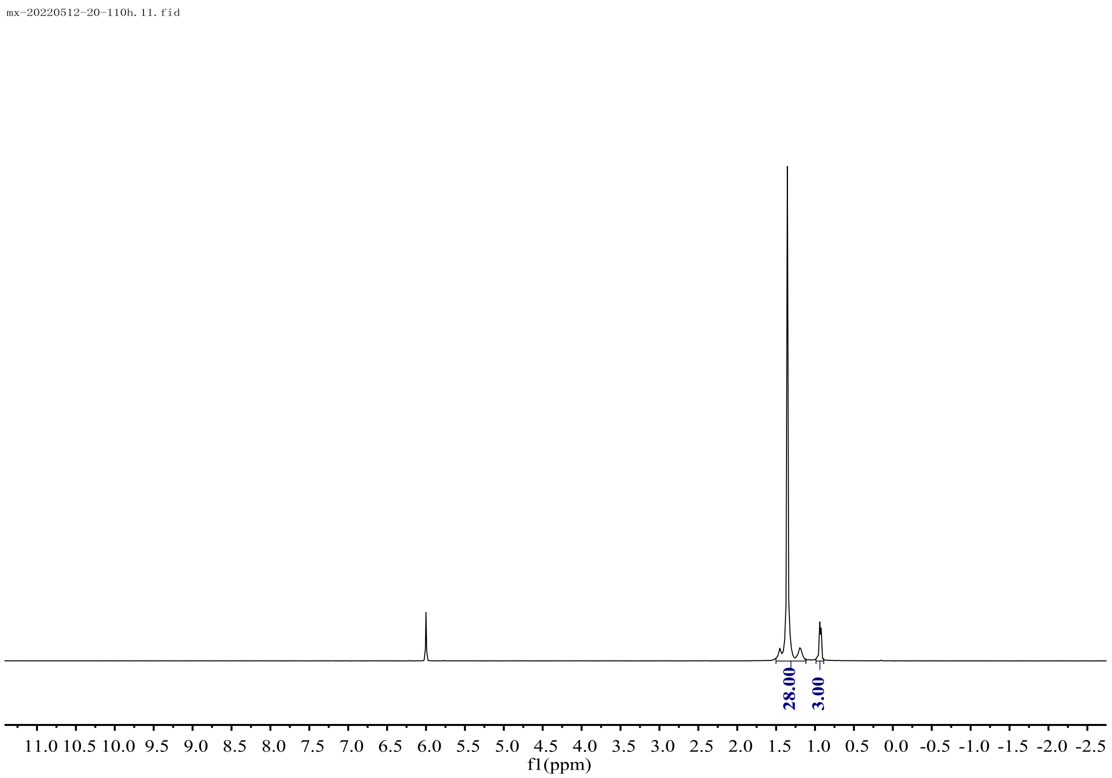

Figure S7. ^{1}H NMR spectrum (400 MHz, $C_2D_2Cl_4$, 110 °C) of polyethylene generated by **Ipty/^{i}Pr-Ni** from table 1, entry 2.

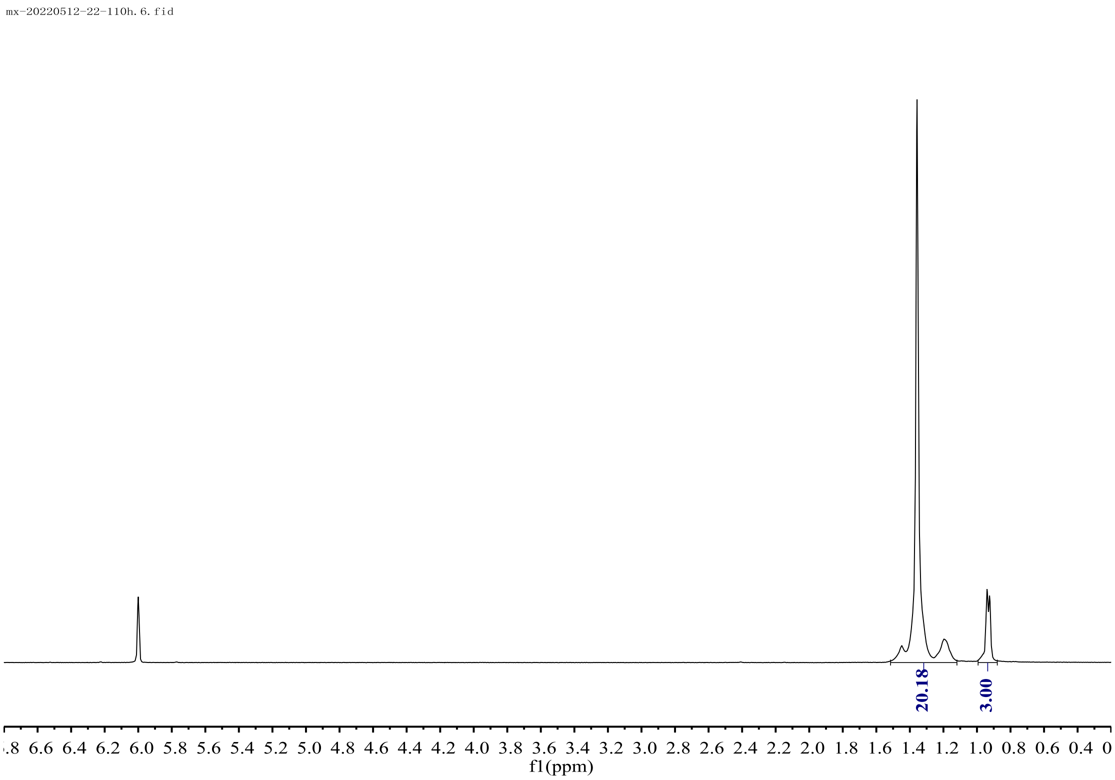

Figure S8. ^{1}H NMR spectrum (400 MHz, $C_2D_2Cl_4$, 110 °C) of polyethylene generated by **Ipty/^{i}Pr-Ni** from table 1, entry 3.

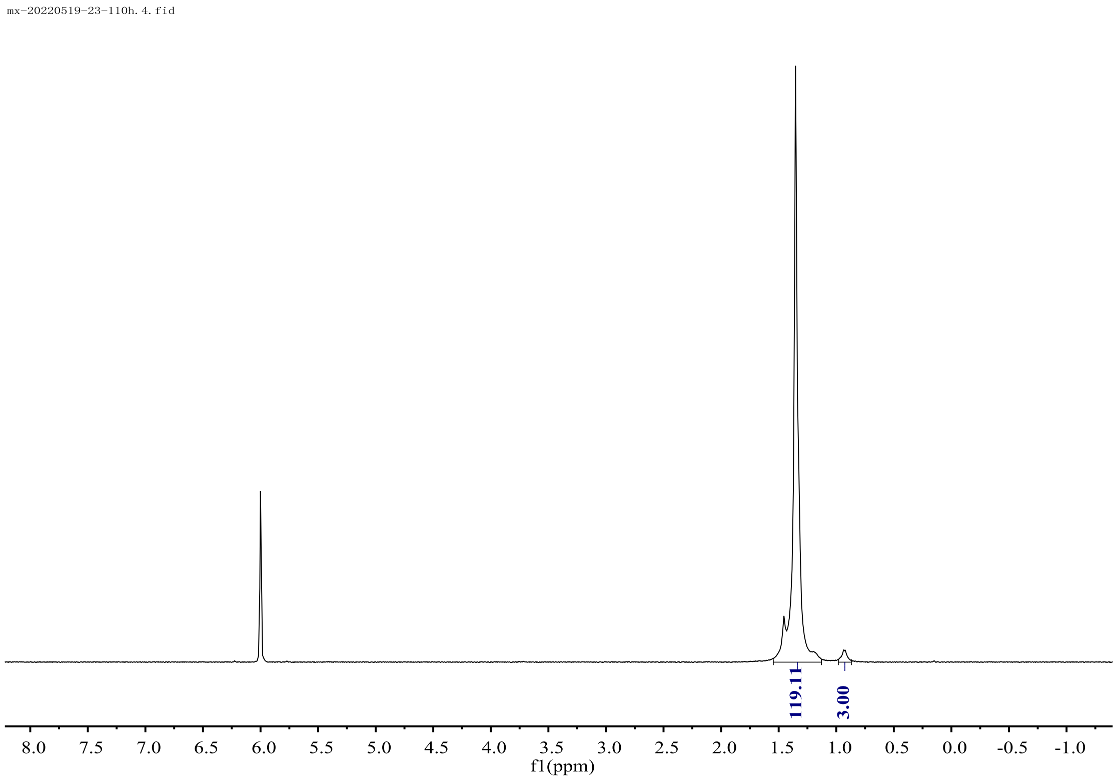

Figure S9. ^{1}H NMR spectrum (400 MHz, $C_2D_2Cl_4$, 110 °C) of polyethylene generated by **Ipty/^{i}Pr-Ni** from table 2, entry 1.

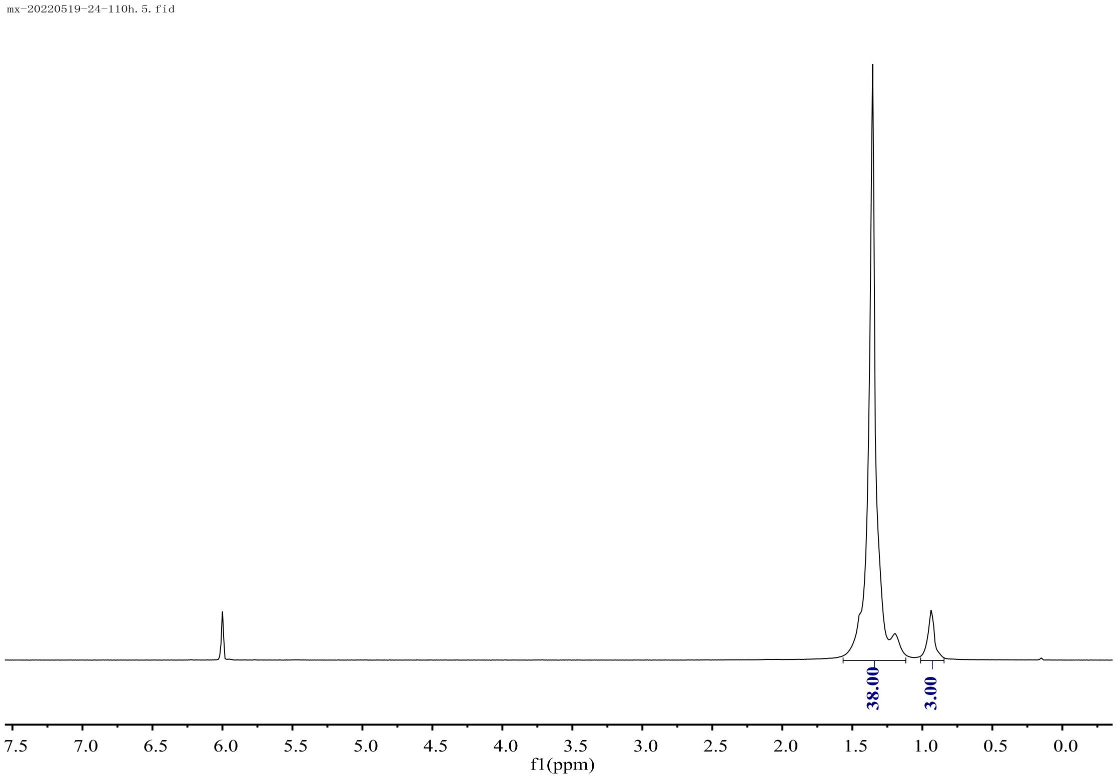

Figure S10. ^{1}H NMR spectrum (400 MHz, $C_2D_2Cl_4$, 110 °C) of polyethylene generated by **Ipty/^{i}Pr-Ni** from table 2, entry 2.

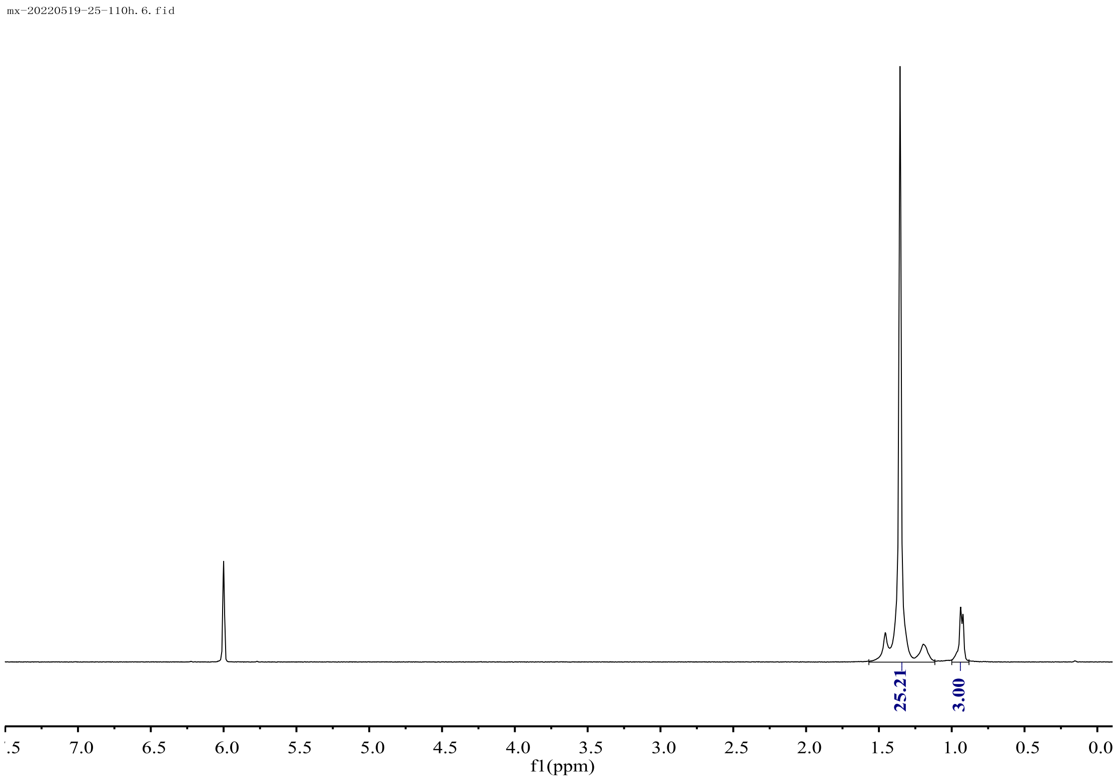

Figure S11. ^{1}H NMR spectrum (400 MHz, $C_2D_2Cl_4$, 110 °C) of polyethylene generated by **Ipty/^{i}Pr-Ni** from table 2, entry 3.

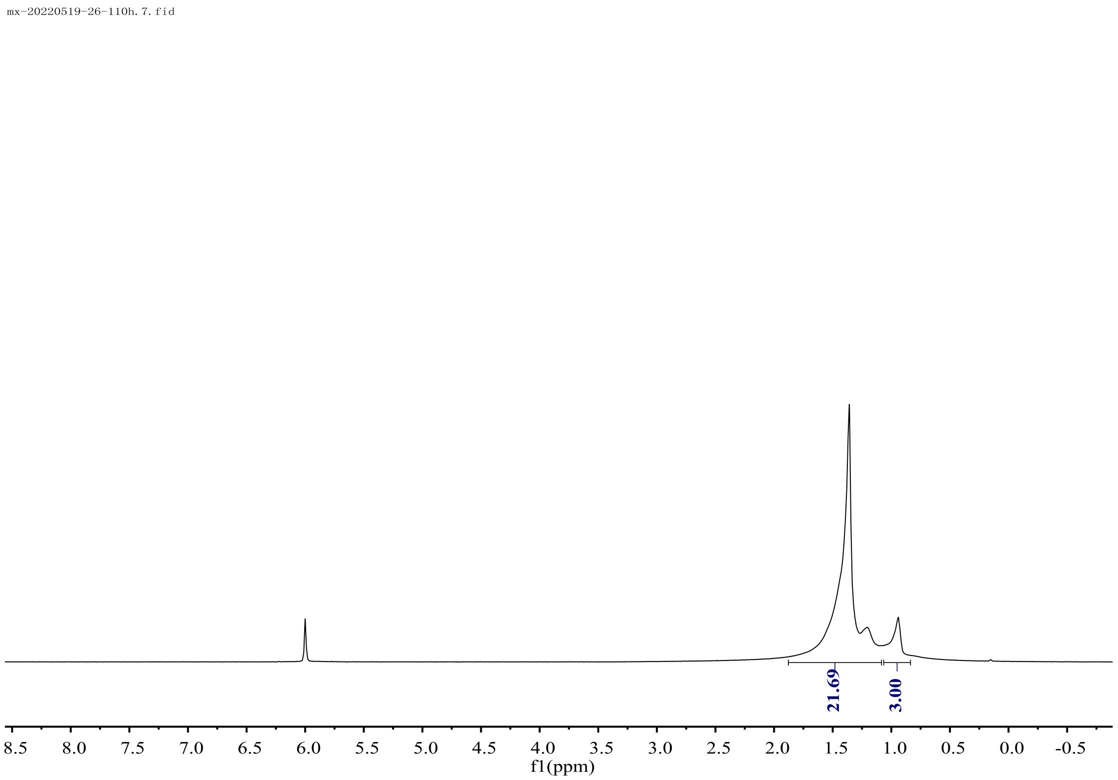

Figure S12. ^{1}H NMR spectrum (400 MHz, $C_2D_2Cl_4$, 110 °C) of polyethylene generated by **Ipty/^{i}Pr-Ni** from table 2, entry 4.

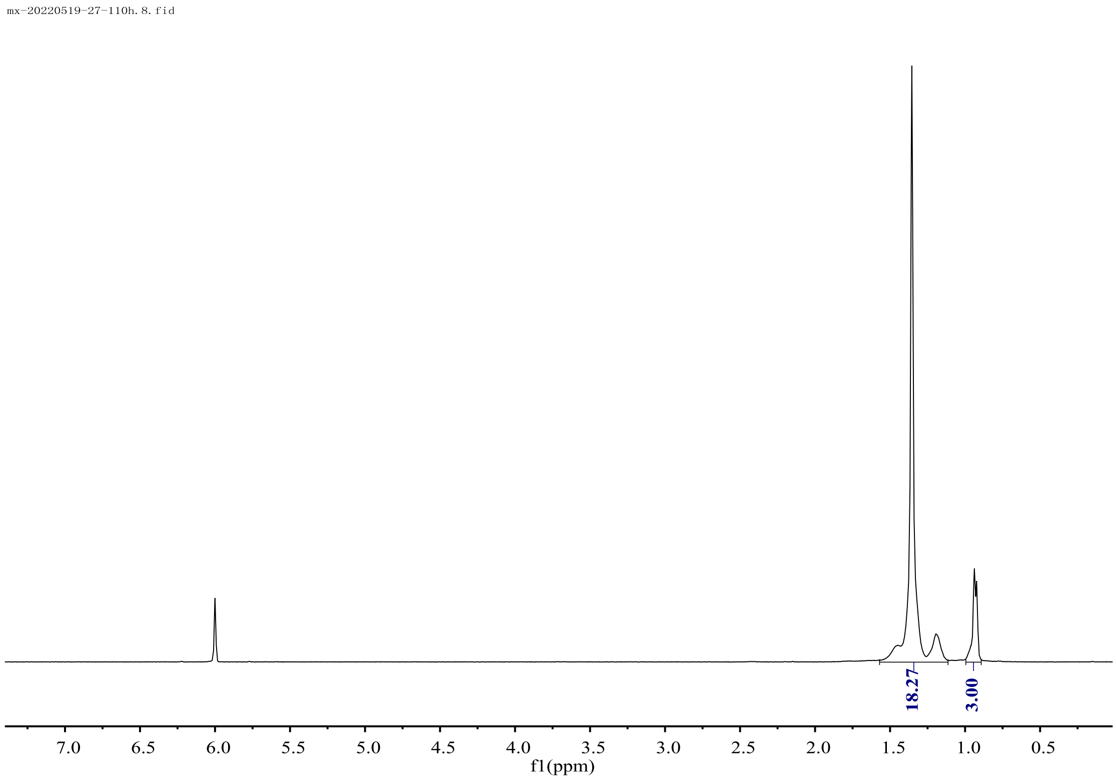

Figure S13. ^{1}H NMR spectrum (400 MHz, $C_2D_2Cl_4$, 110 °C) of polyethylene generated by **Ipty/*i*Pr-Ni** from table 2, entry 5.

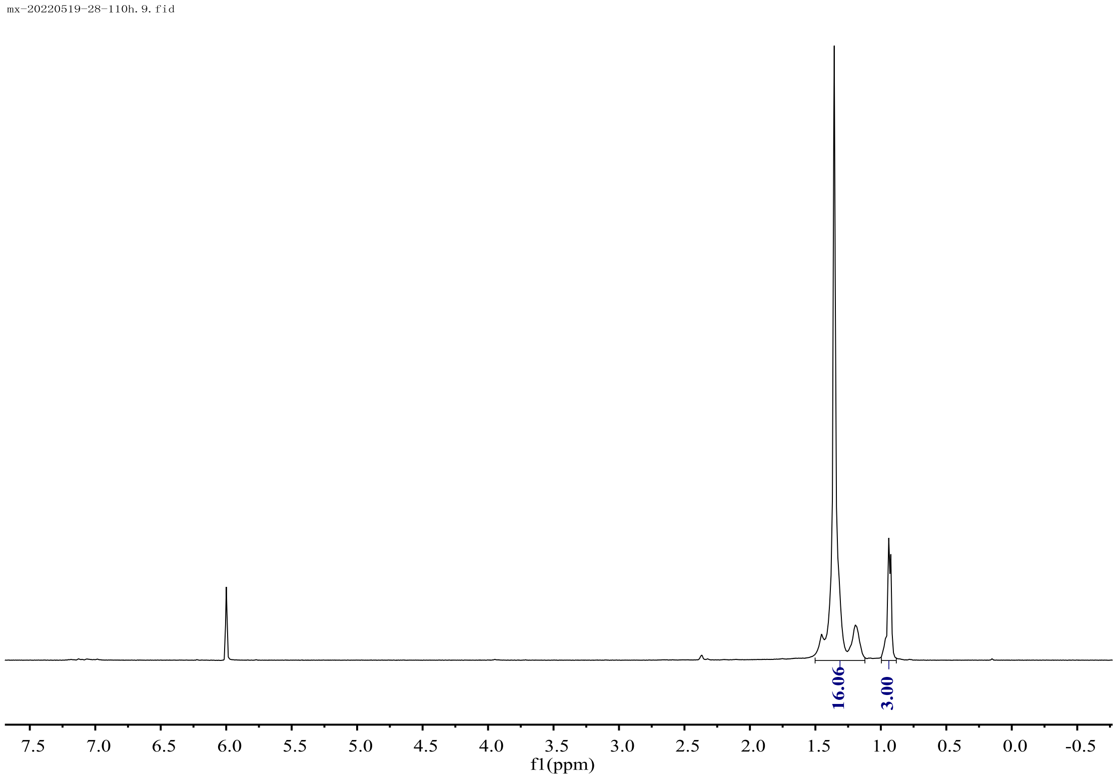

Figure S14. ^{1}H NMR spectrum (400 MHz, $C_2D_2Cl_4$, 110 °C) of polyethylene generated by **Ipty/*i*Pr-Ni** from table 2, entry 6.

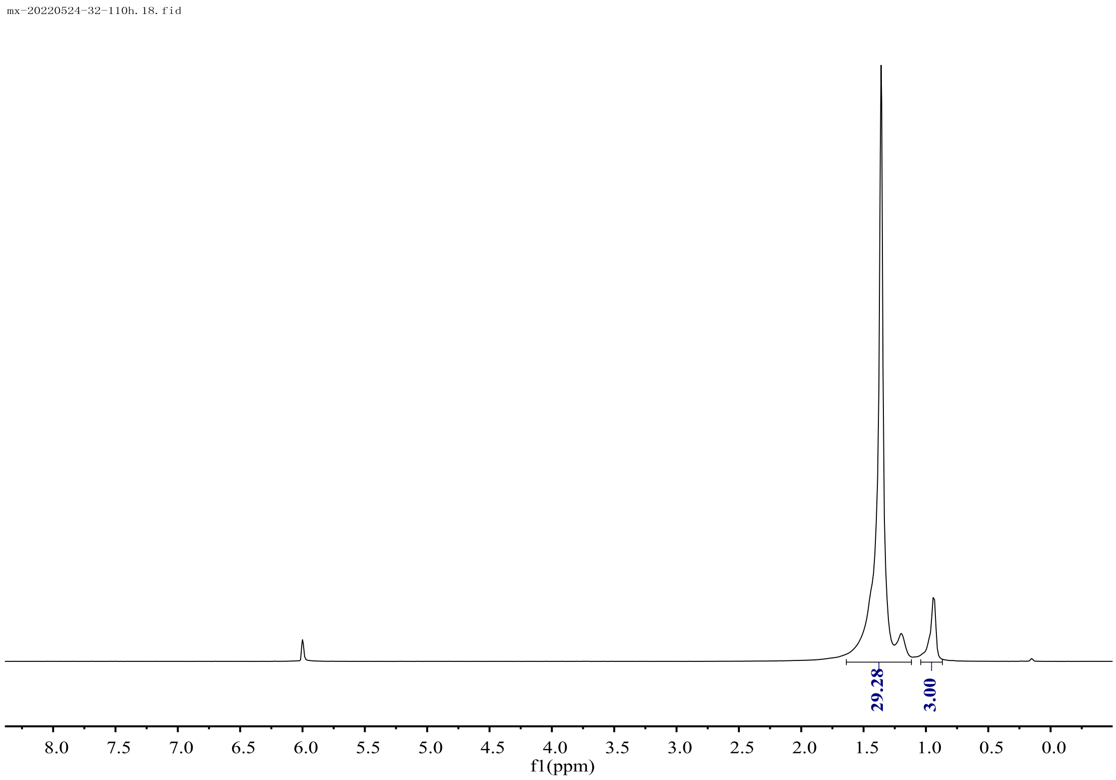

Figure S15. ^{1}H NMR spectrum (400 MHz, $C_2D_2Cl_4$, 110 °C) of polyethylene generated by **Ipty/^{i}Pr-Ni** from table 2, entry 7.

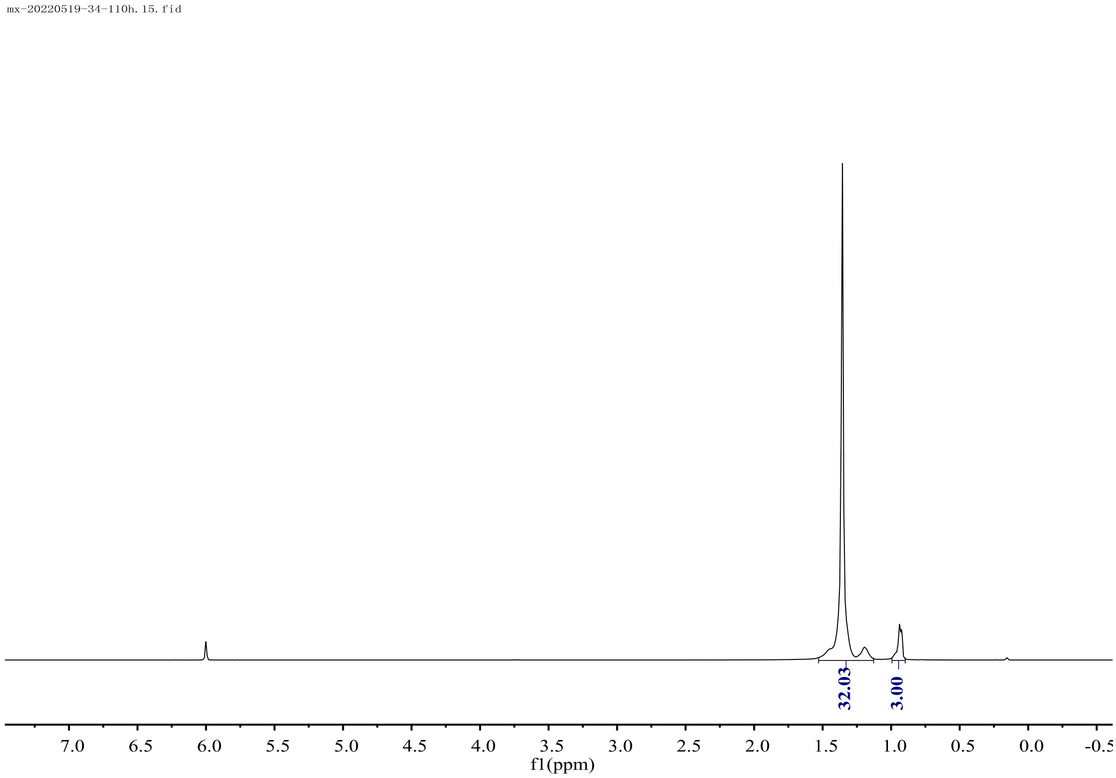

Figure S16. ^{1}H NMR spectrum (400 MHz, $C_2D_2Cl_4$, 110 °C) of polyethylene generated by **^{i}Pr-Ni** from table 2, entry 8.

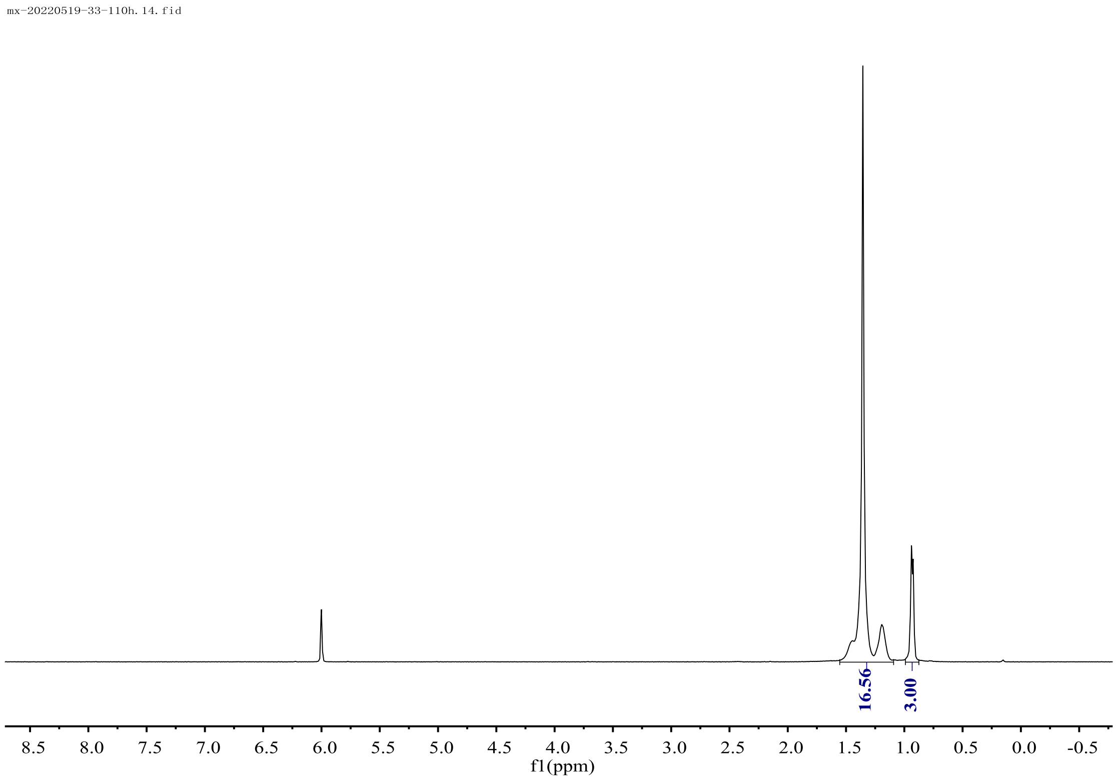

Figure S17. ^{1}H NMR spectrum (400 MHz, $C_2D_2Cl_4$, 110 °C) of the polyethylene generated by **Ipty-Ni** from table 2, entry 9.

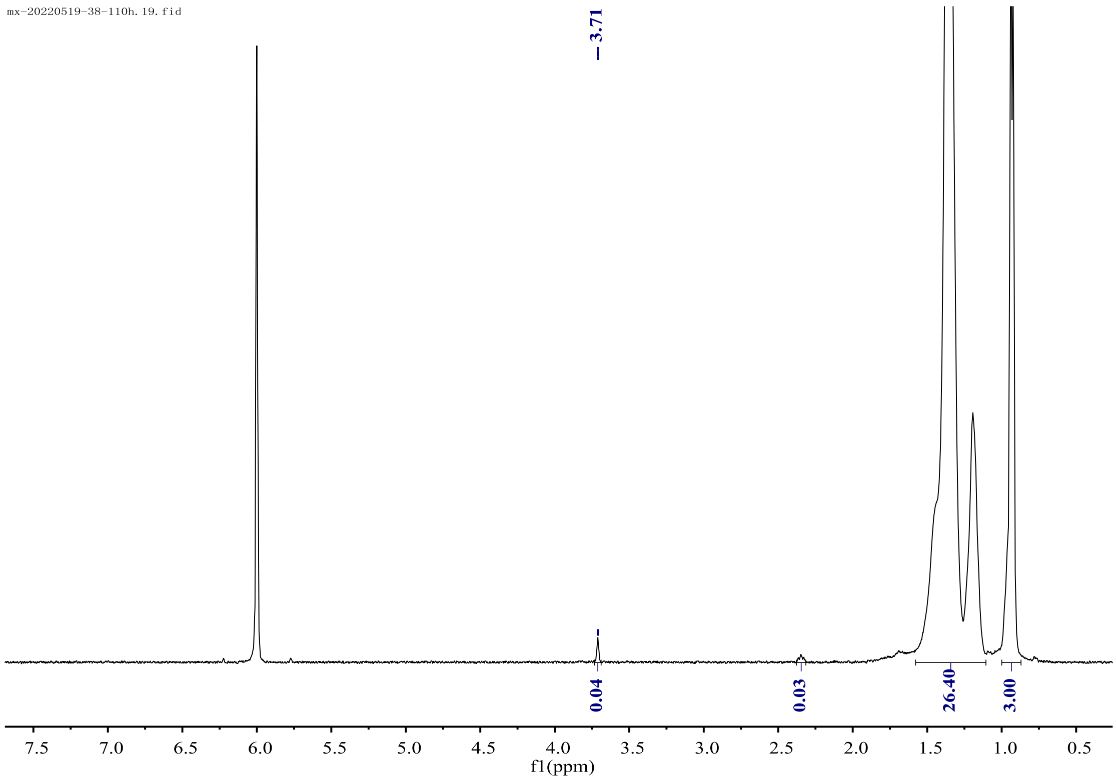

Figure S18. ^{1}H NMR spectrum (400 MHz, $C_2D_2Cl_4$, 110 °C) of the copolymer generated by **Ipty/^{i}Pr-Ni** from table 3, entry 2.

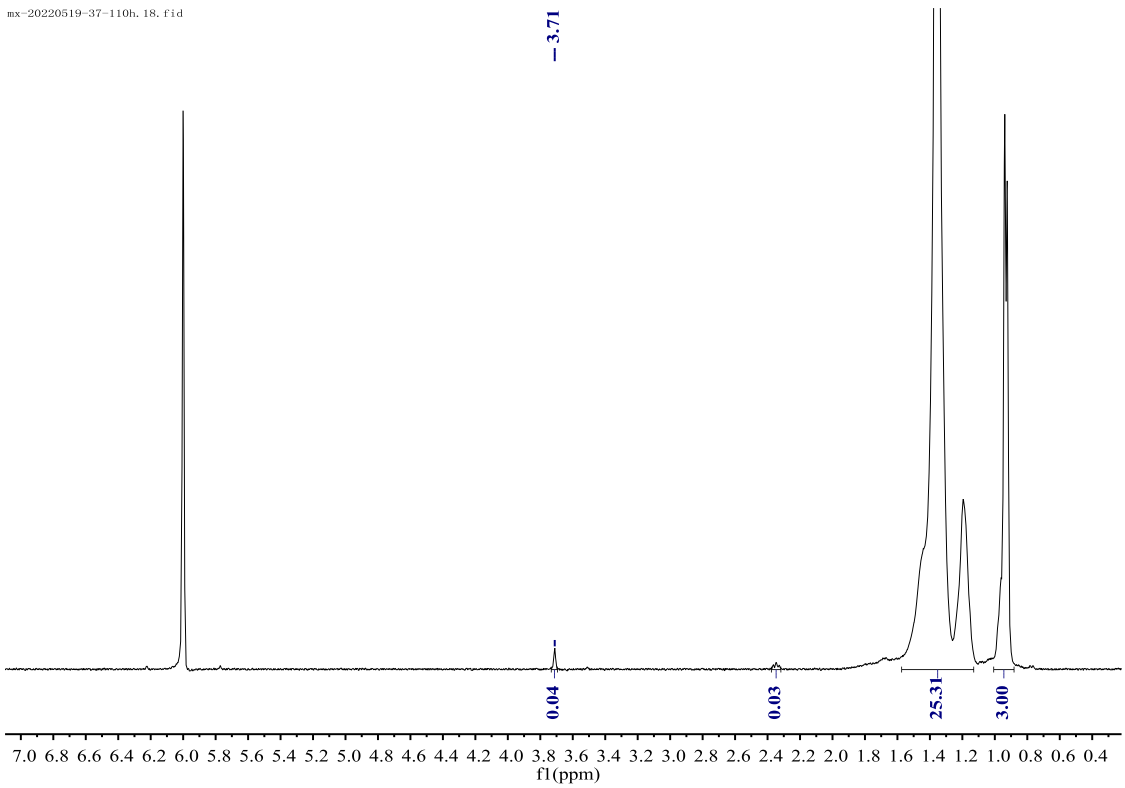

Figure S19. ^{1}H NMR spectrum (400 MHz, $C_2D_2Cl_4$, 110 °C) of the copolymer generated by **Ipty/*i*Pr-Ni** from table 3, entry 3.

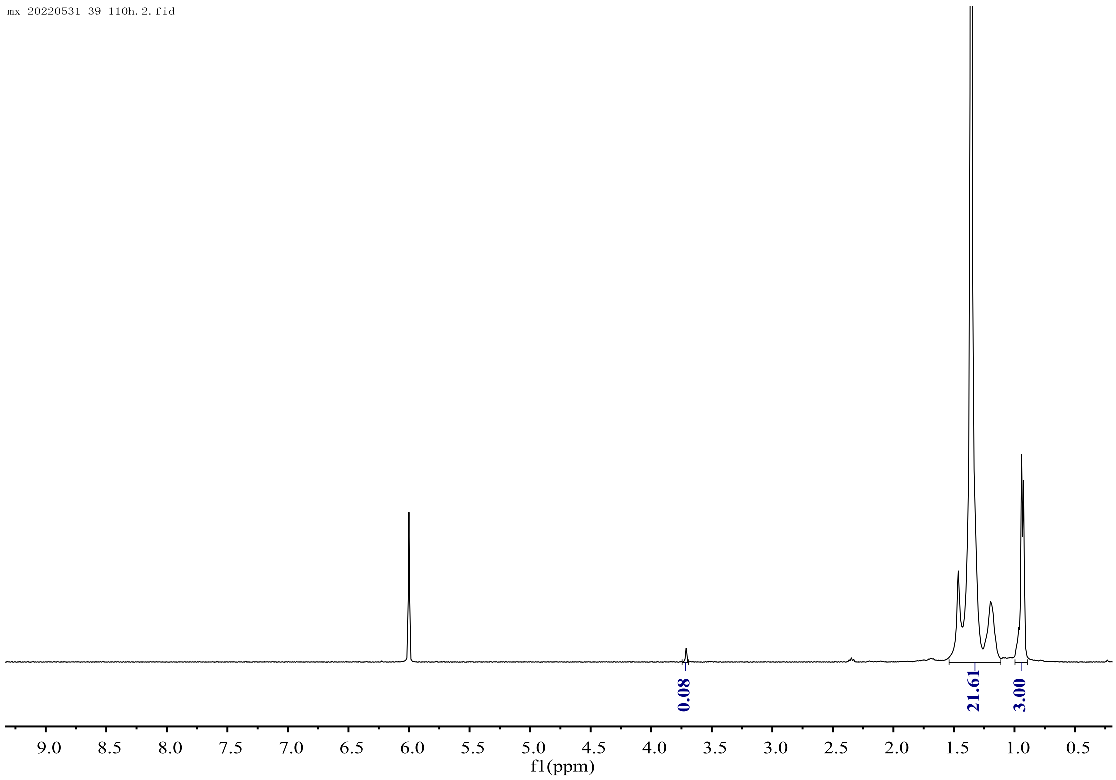

Figure S20. ^{1}H NMR spectrum (400 MHz, $C_2D_2Cl_4$, 110 °C) of the copolymer generated by **Ipty/*i*Pr-Ni** from table 3, entry 4.

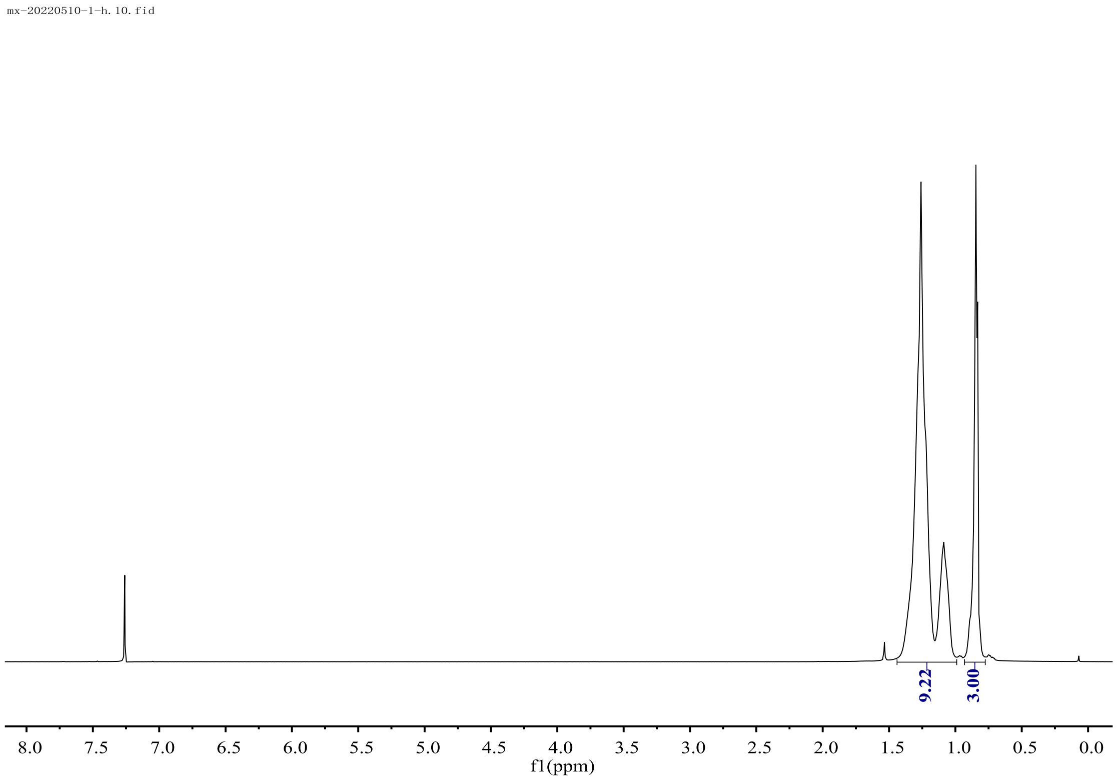

Figure S21. ^{1}H NMR spectrum (500 MHz, $CDCl_3$, 25 °C) of polyethylene generated by **Ipty/^{i}Pr-Pd** from table 4, entry 1.

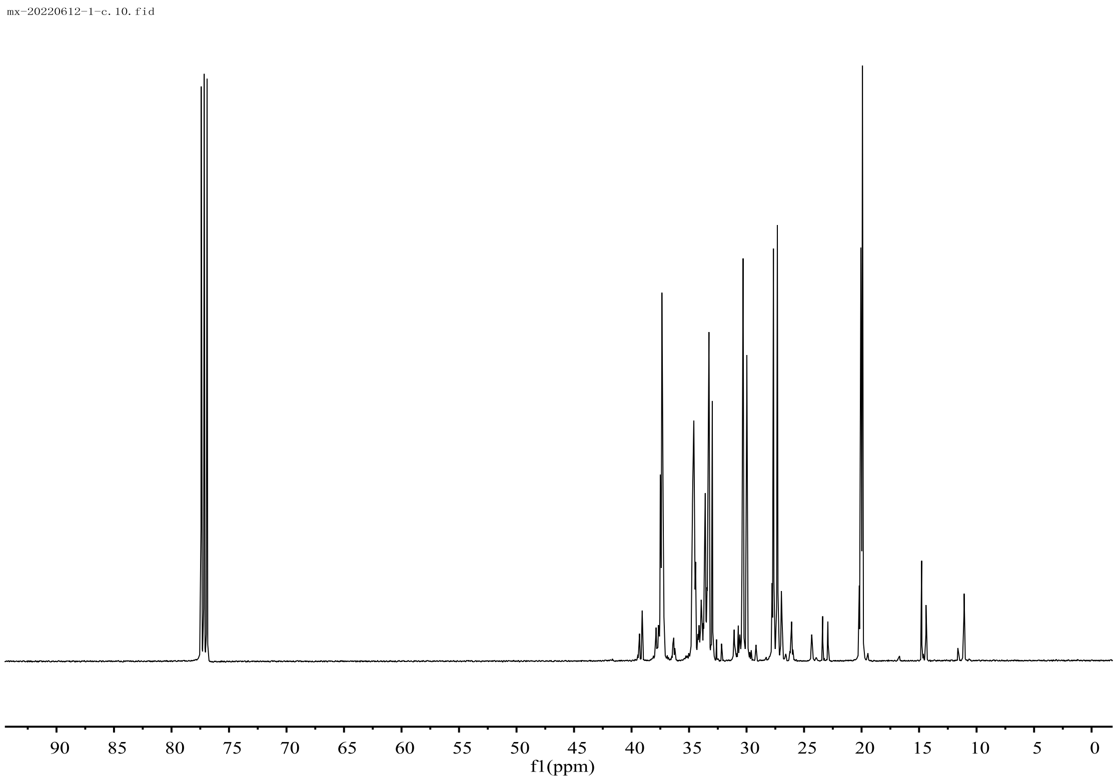

Figure S22. ^{13}C NMR spectrum (500 MHz, $CDCl_3$, 25 °C) of polyethylene generated by **Ipty/^{i}Pr-Pd** from table 4, entry 1.

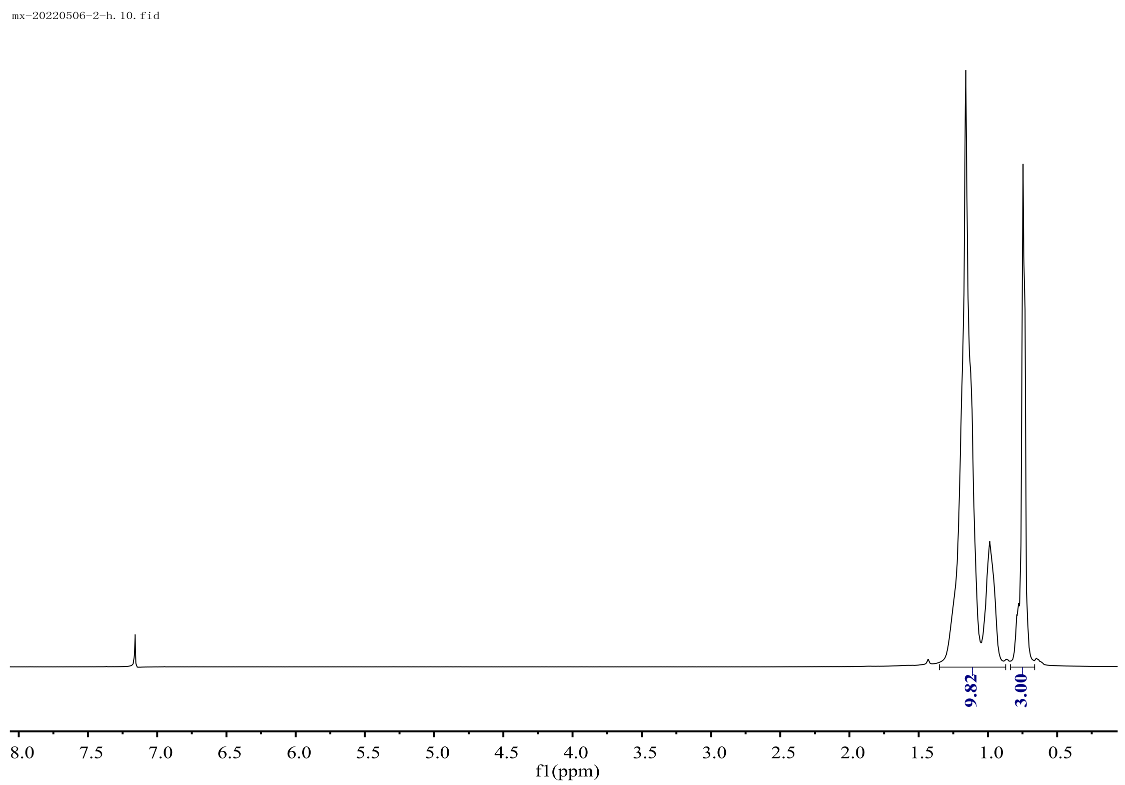

Figure S23. ^{1}H NMR spectrum (500 MHz, $CDCl_3$, 25 °C) of polyethylene generated by **Ipty/*i*Pr-Pd** from table 4, entry 2.

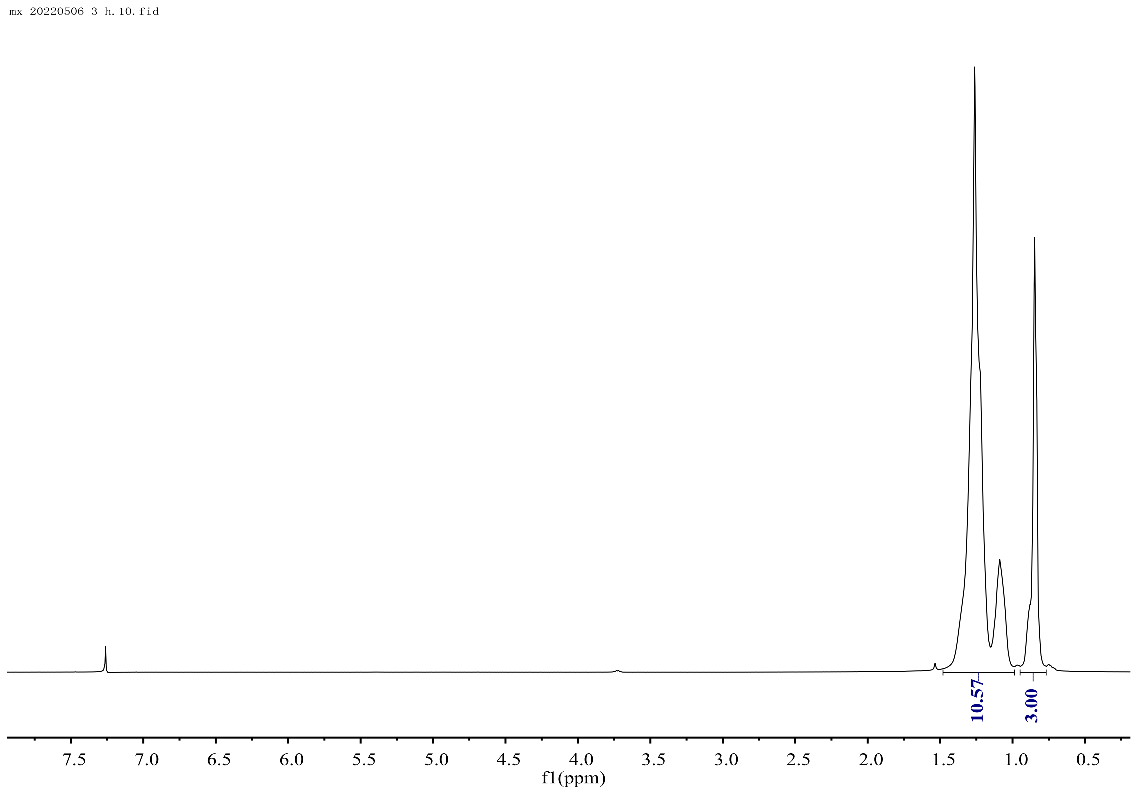

Figure S24. ^{1}H NMR spectrum (500 MHz, $CDCl_3$, 25 °C) of polyethylene generated by **Ipty/*i*Pr-Pd** from table 4, entry 3.

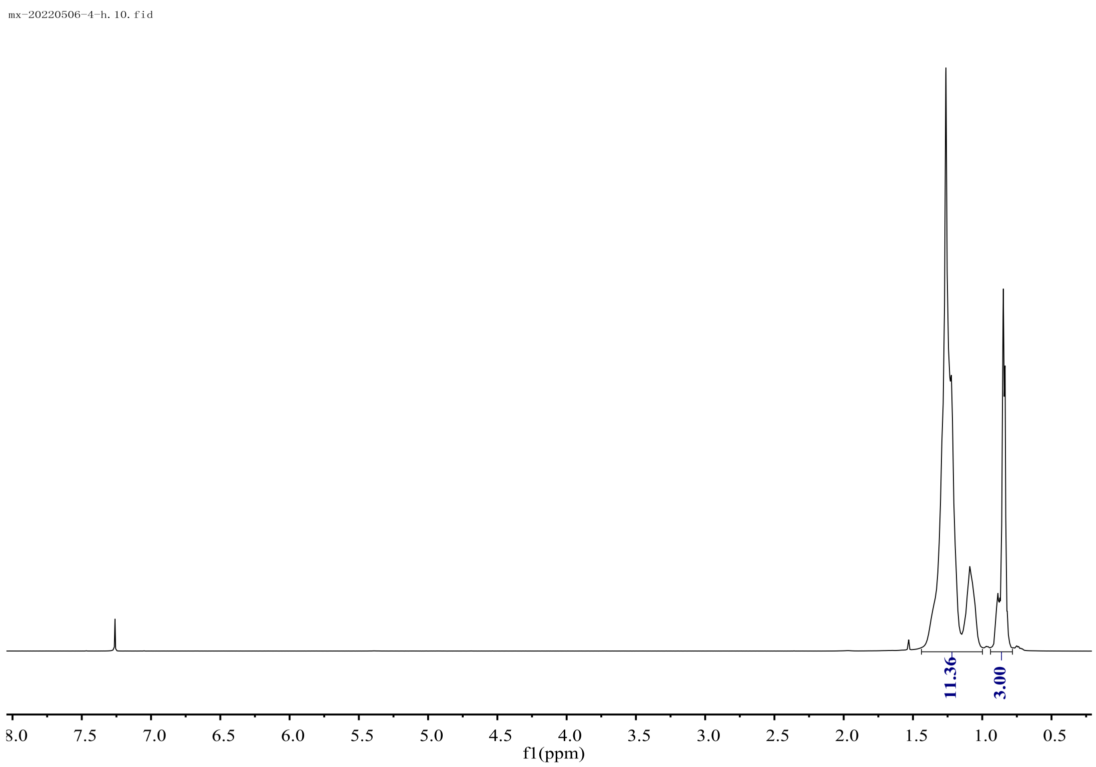

Figure S25. ^{1}H NMR spectrum (500 MHz, $CDCl_3$, 25 °C) of polyethylene generated by **Ipty/^{i}Pr-Pd** from table 4, entry 4.

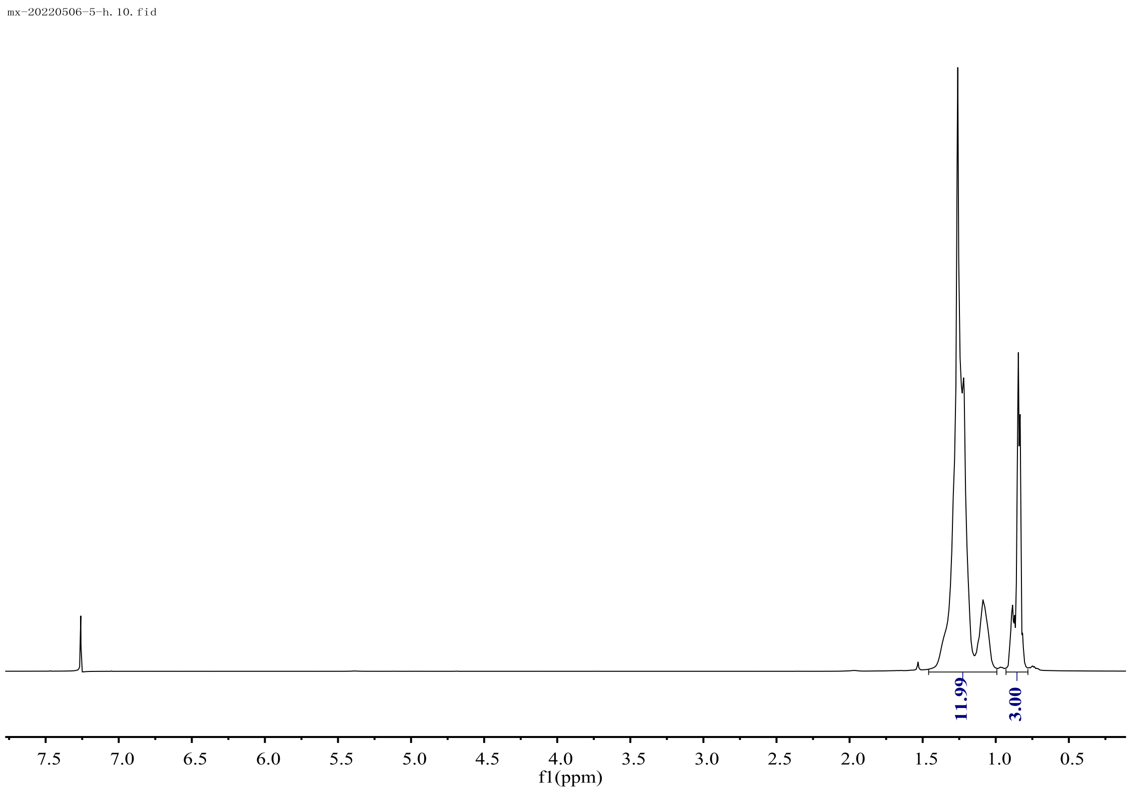

Figure S26. ^{1}H NMR spectrum (500 MHz, $CDCl_3$, 25 °C) of polyethylene generated by **Ipty/^{i}Pr-Pd** from table 4, entry 5.

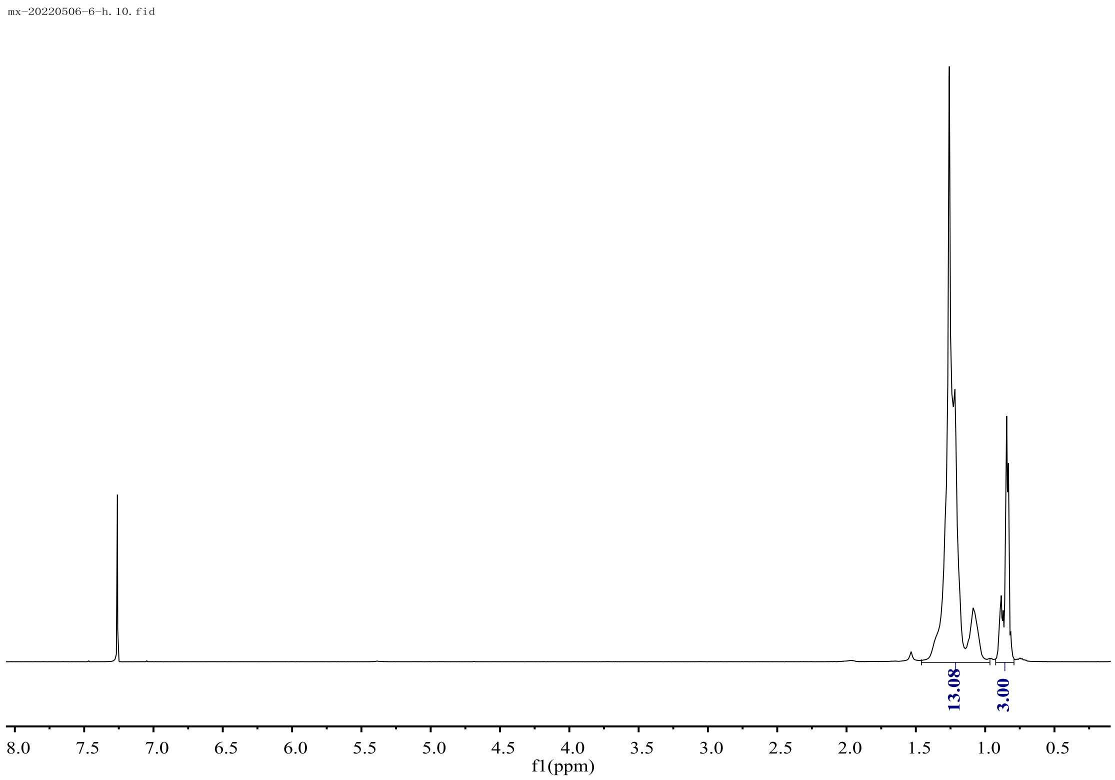

Figure S27. ^{1}H NMR spectrum (500 MHz, $CDCl_3$, 25 °C) of polyethylene generated by **Ipty/^{i}Pr-Pd** from table 4, entry 6.

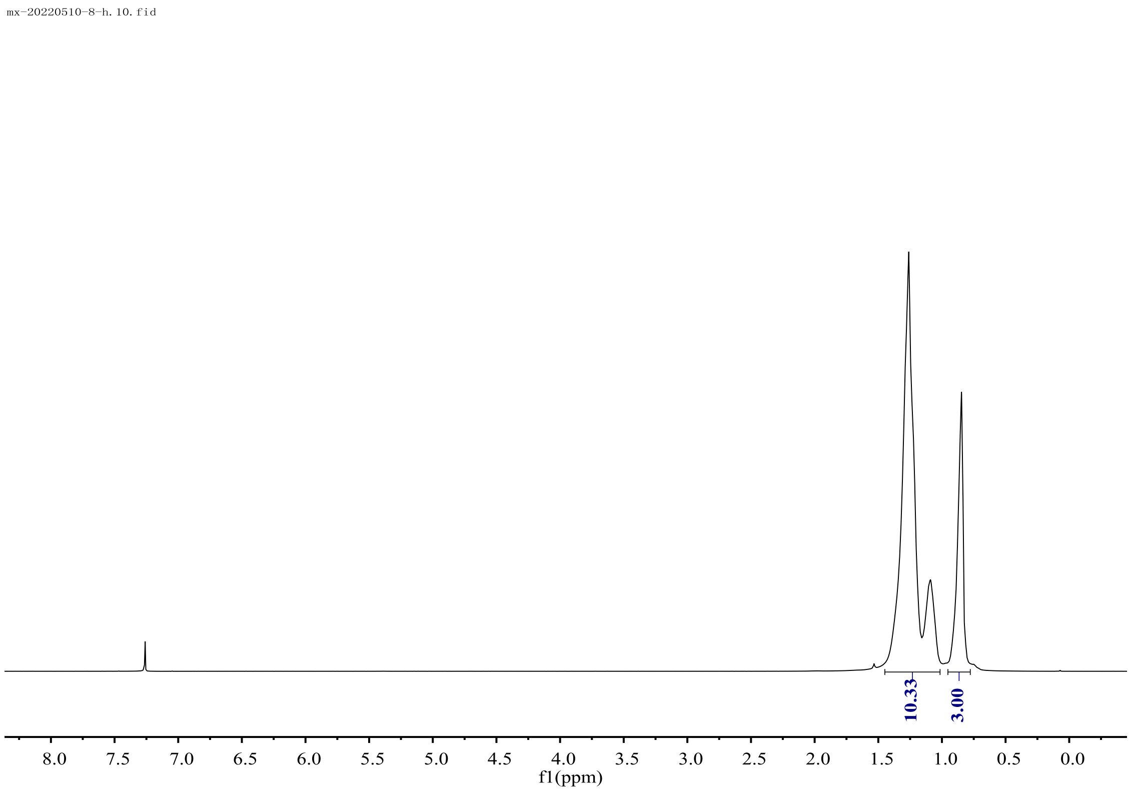

Figure S28. ^{1}H NMR spectrum (500 MHz, $CDCl_3$, 25 °C) of polyethylene generated by **Ipty/^{i}Pr-Pd** from table 4, entry 7.

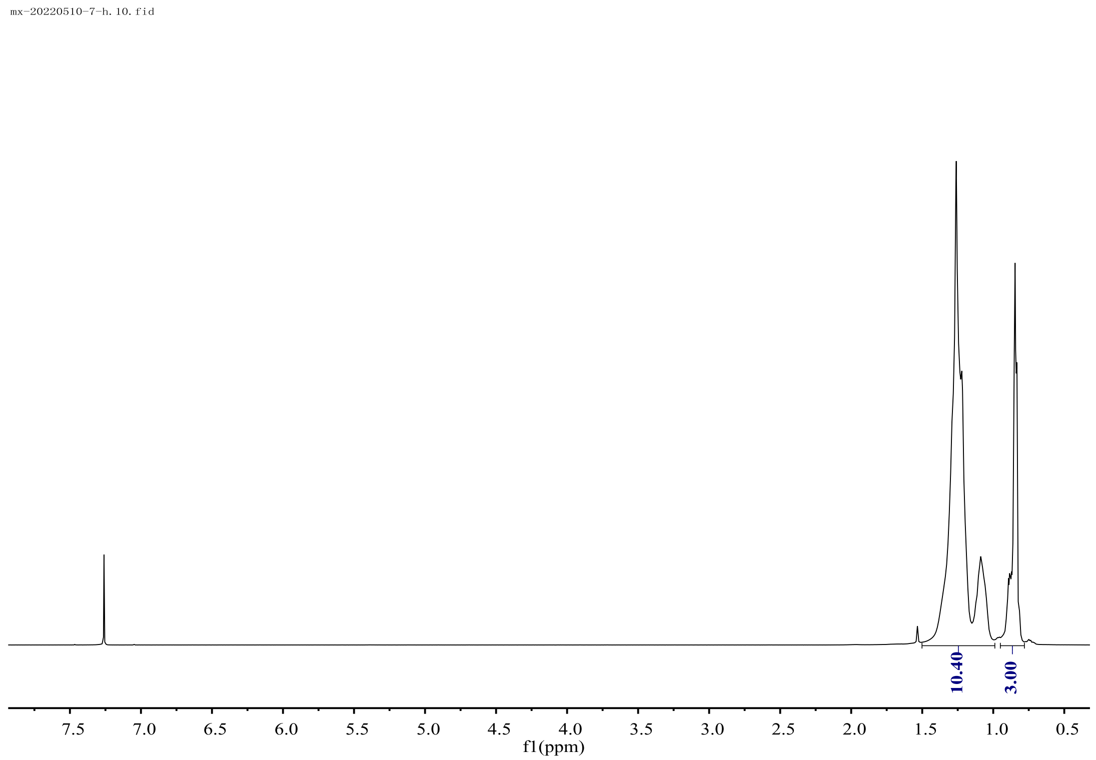

Figure S29. ^{1}H NMR spectrum (500 MHz, $CDCl_3$, 25 °C) of polyethylene generated by **Ipty/*i*Pr-Pd** from table 4, entry 8.

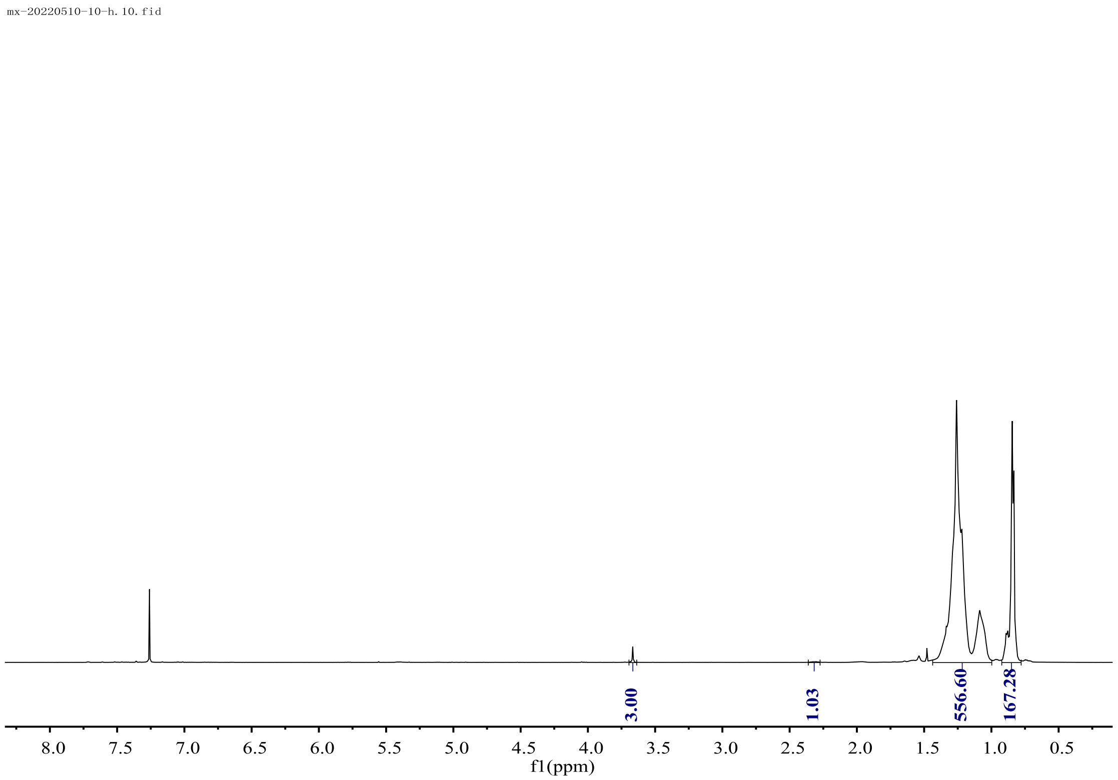

Figure S30. ^{1}H NMR spectrum (500 MHz, $CDCl_3$, 25 °C) of the copolymer generated by **Ipty/*i*Pr-Pd** from table 5, entry 1.

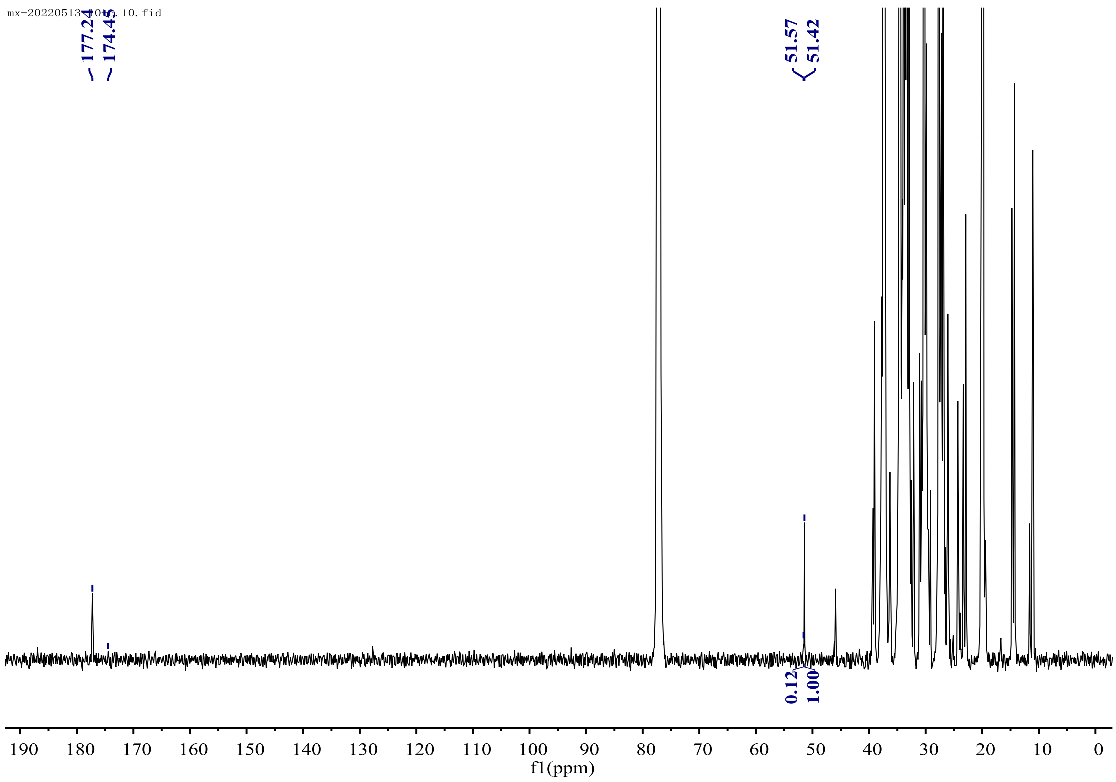

Figure S31. ^{13}C NMR spectrum (500 MHz, $CDCl_3$, 25 °C) of the copolymer generated by **Ipty/^{i}Pr-Pd** from table 5, entry 1.

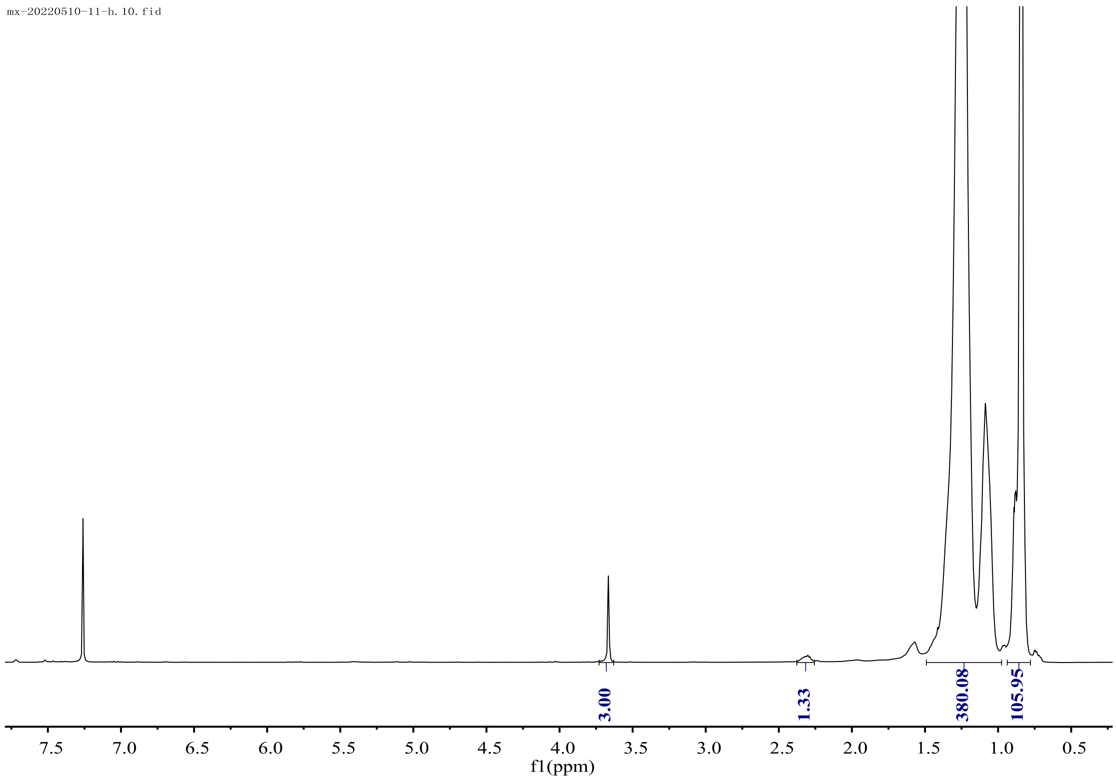

Figure S32. ^1H NMR spectrum (500 MHz, $CDCl_3$, 25 °C) of the copolymer generated by **Ipty/^{i}Pr-Pd** from table 5, entry 2.

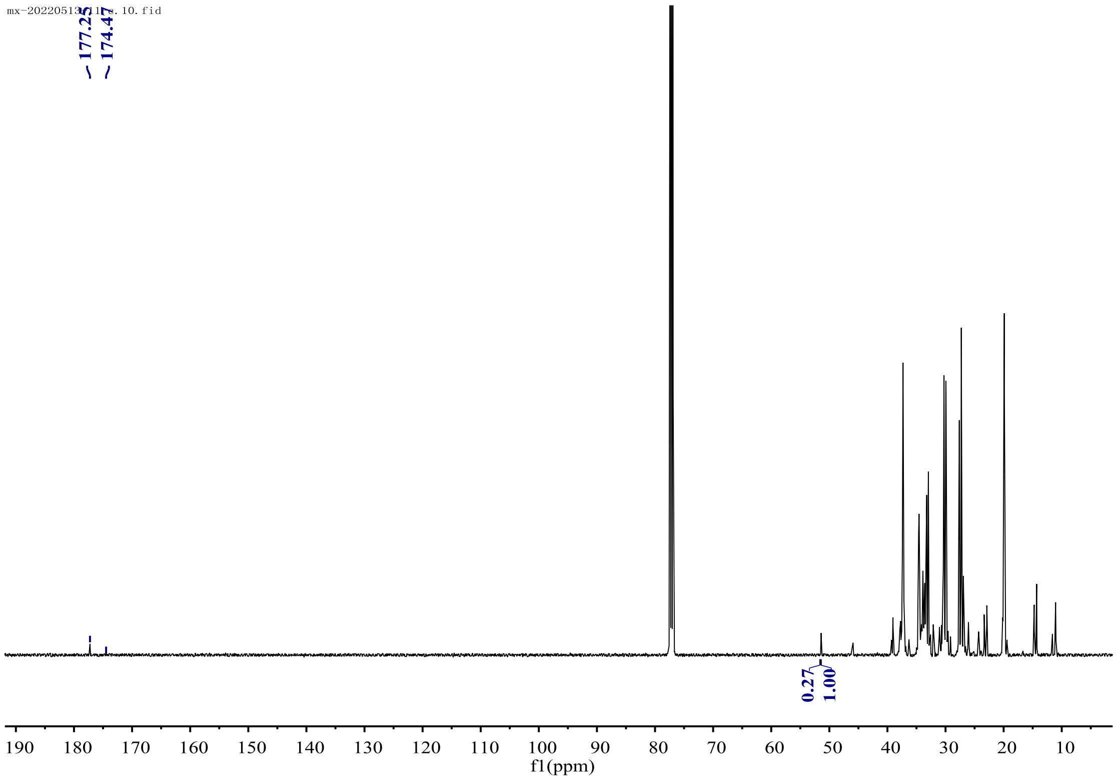

Figure S33. ^{13}C NMR spectrum (500 MHz, $CDCl_3$, 25 °C) of the copolymer generated by **Ipty/^{i}Pr-Pd** from table 5, entry 2.

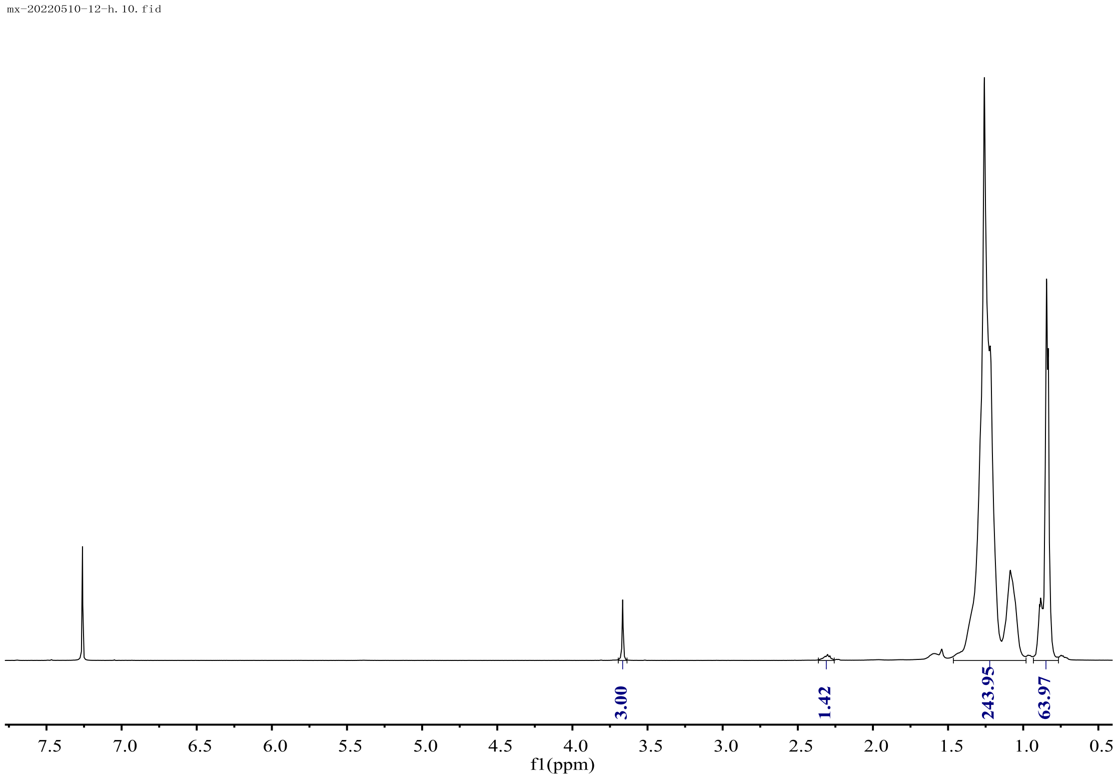

Figure S34. ^1H NMR spectrum (500 MHz, $CDCl_3$, 25 °C) of the copolymer generated by **Ipty/^{i}Pr-Pd** from table 5, entry 3.

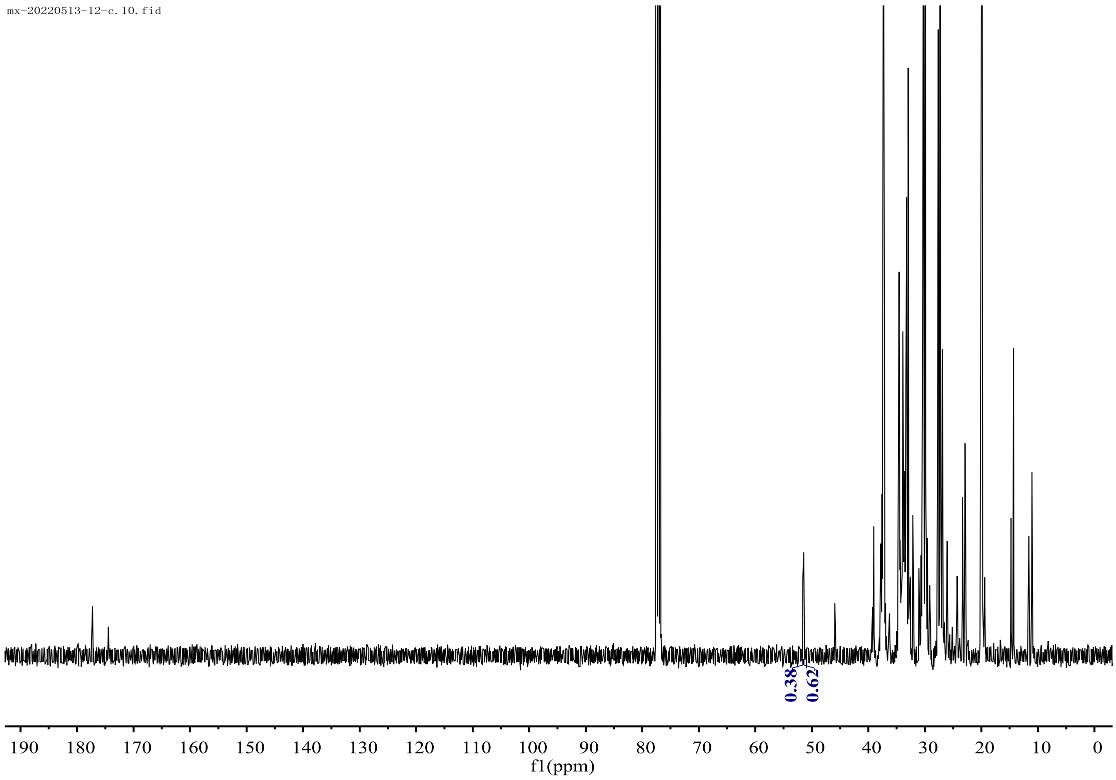

Figure S35. ^{13}C NMR spectrum (500 MHz, $CDCl_3$, 25 °C) of the copolymer generated by **Ipty/*ⁱ*Pr-Pd** from table 5, entry 3.

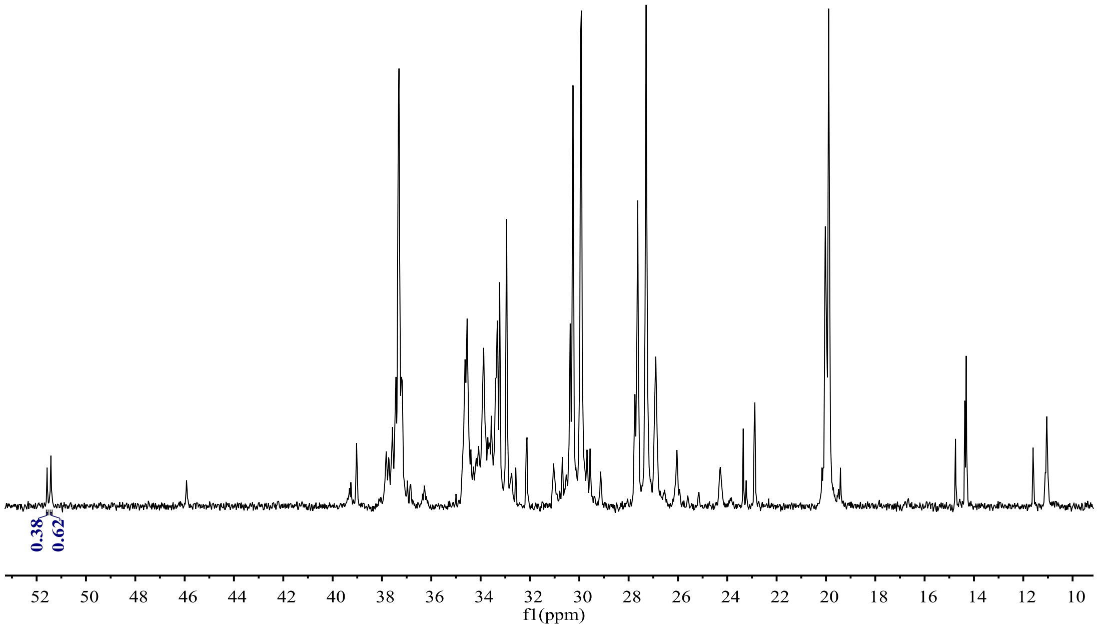

Figure S36. Partial enlargement of ^{13}C NMR spectrum (500 MHz, $CDCl_3$, 25 °C) of the copolymer generated by **Ipty/^{i}Pr-Pd** from table 5, entry 3.

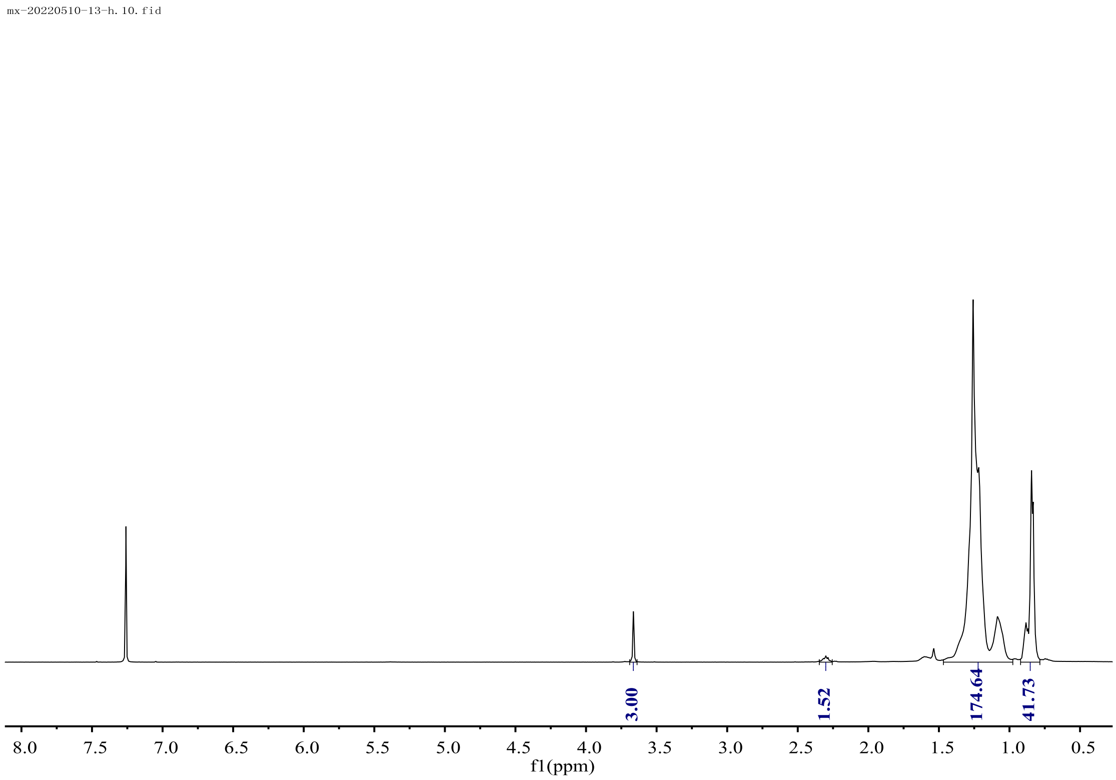

Figure S37. ^{1}H NMR spectrum (500 MHz, $CDCl_3$, 25 °C) of the copolymer generated by **Ipty/^{i}Pr-Pd** from table 5, entry 4.

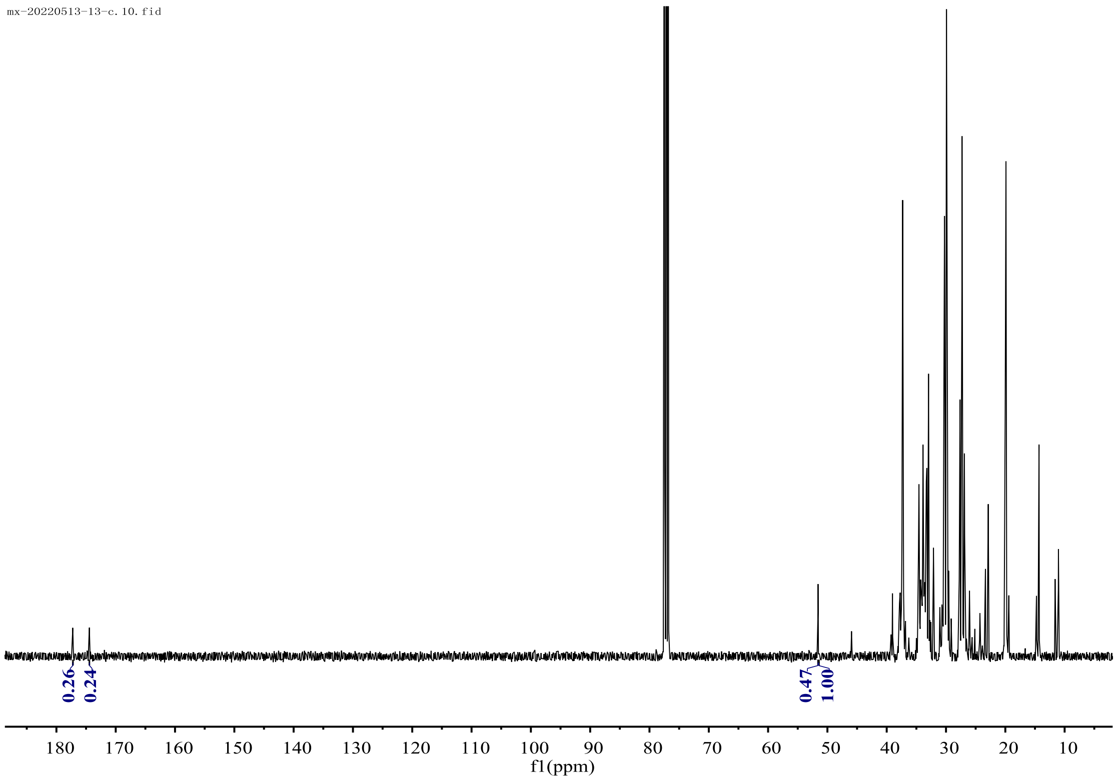

Figure S38. ^{13}C NMR spectrum (500 MHz, $CDCl_3$, 25 °C) of the copolymer generated by **Ipty/^{i}Pr-Pd** from table 5, entry 4.

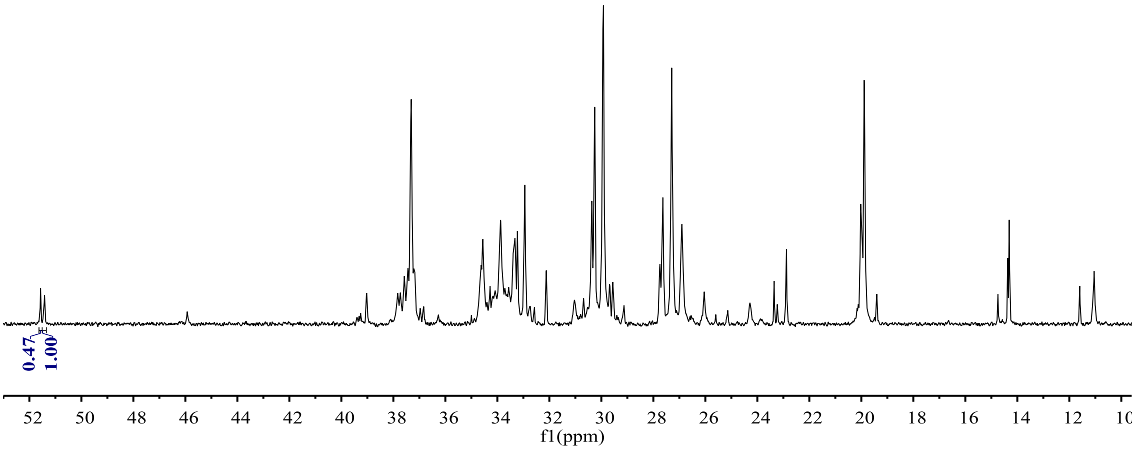

Figure S39. Partial enlargement of ^{13}C NMR spectrum (500 MHz, $CDCl_3$, 25 °C) of the copolymer generated by **Ipty/^{i}Pr-Pd** from table 5, entry 4.

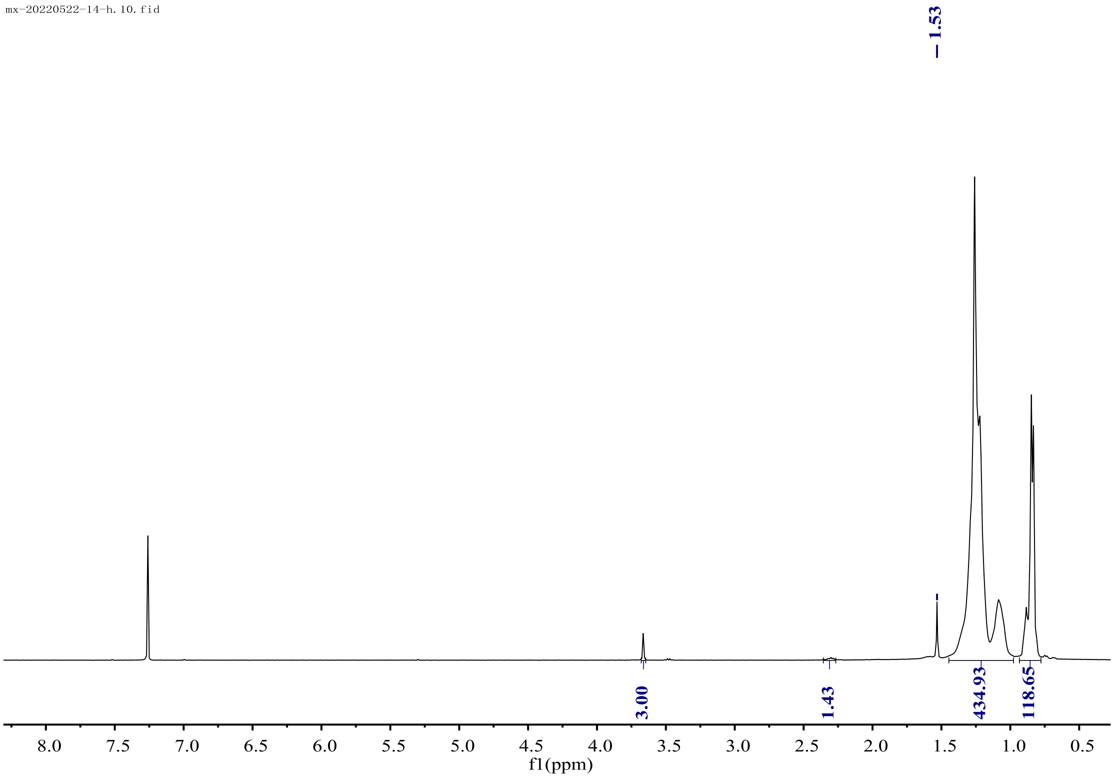

Figure S40. ^1H NMR spectrum (500 MHz, $CDCl_3$, 25 °C) of the copolymer generated by **Ipty/^{i}Pr-Pd** from table 5, entry 5.

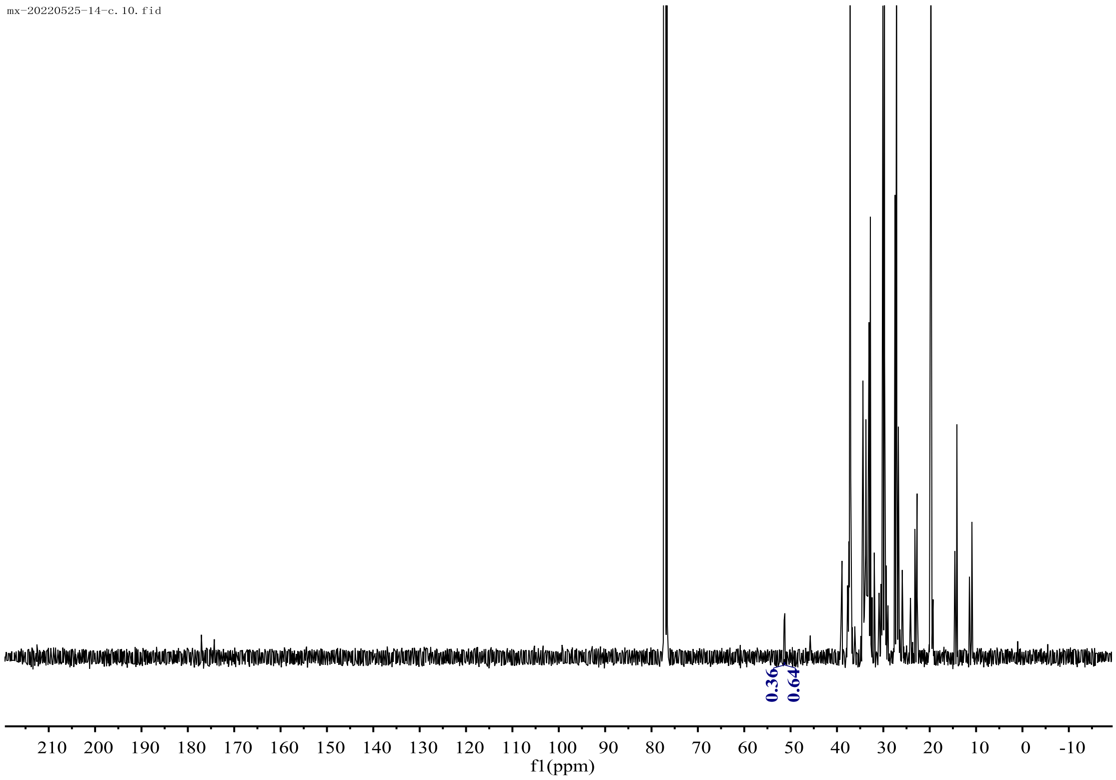

FigureS41. ^{13}C NMR spectrum (500 MHz, $CDCl_3$, 25 °C) of the copolymer generated by **Ipty/^{i}Pr-Pd** from table 5, entry 5.

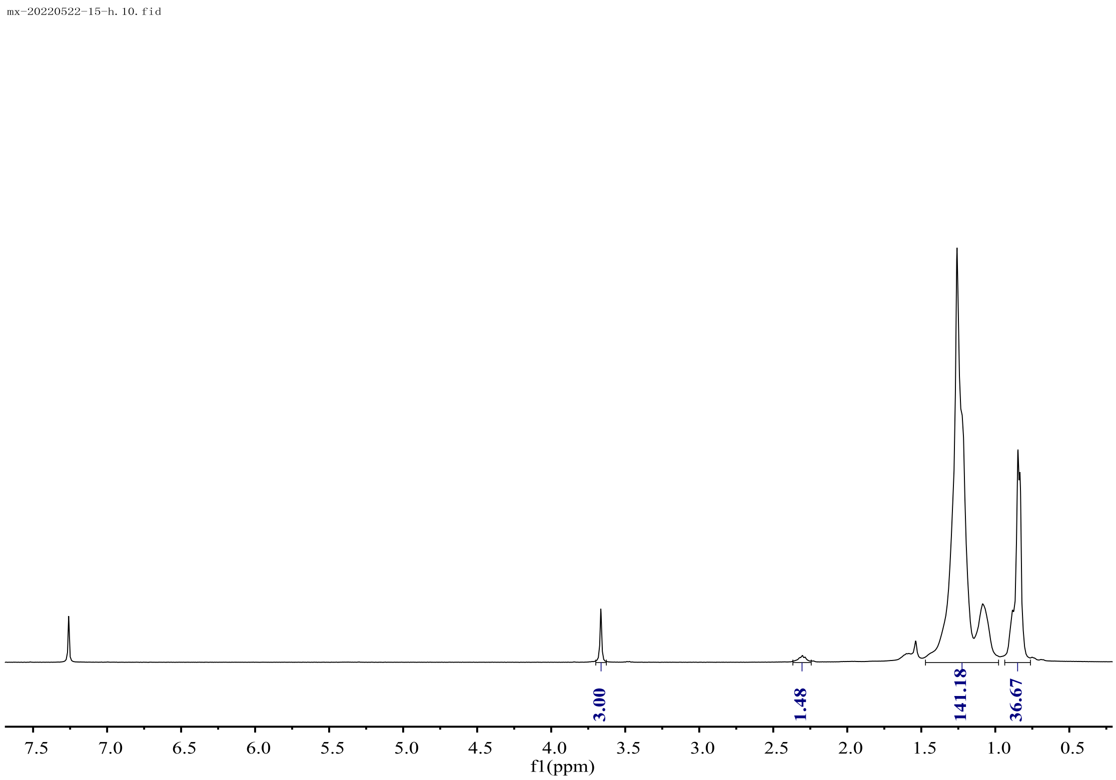

Figure S42. ^1H NMR spectrum (500 MHz, $CDCl_3$, 25 °C) of the copolymer generated by **Ipty/^{i}Pr-Pd** from table 5, entry 6.

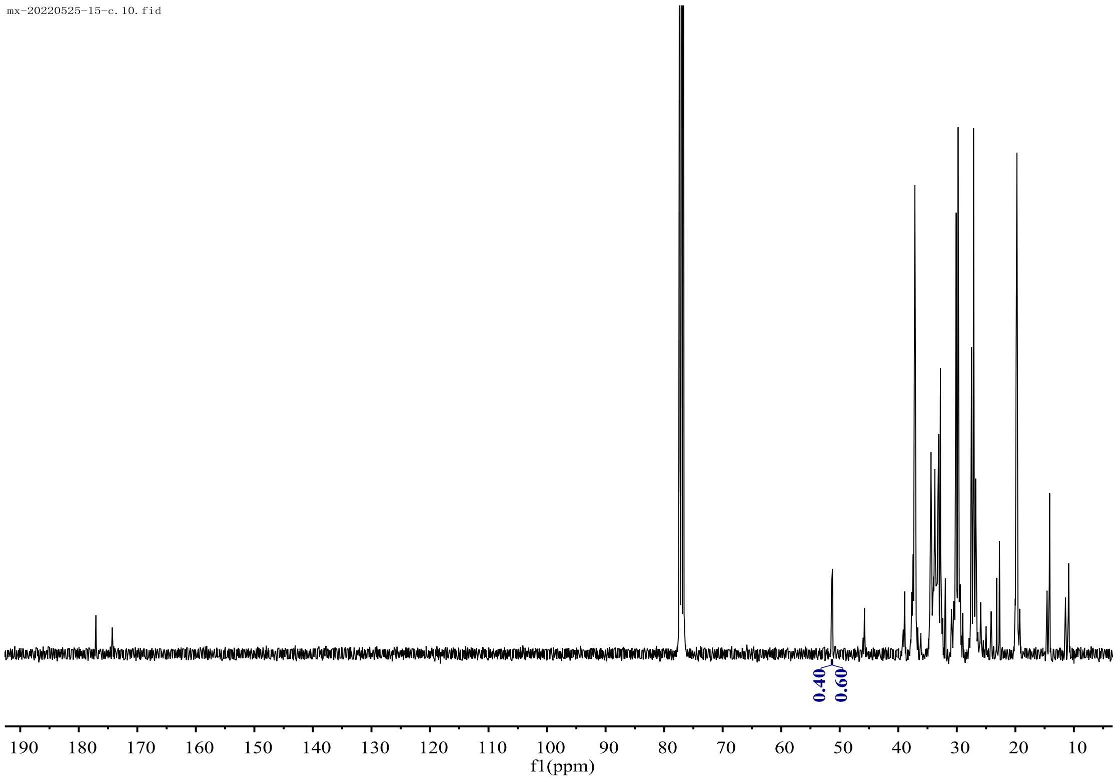

Figure S43. ^{13}C NMR spectrum (500 MHz, $CDCl_3$, 25 °C) of the copolymer generated by **Ipty/^{i}Pr-Pd** from table 5, entry 6.

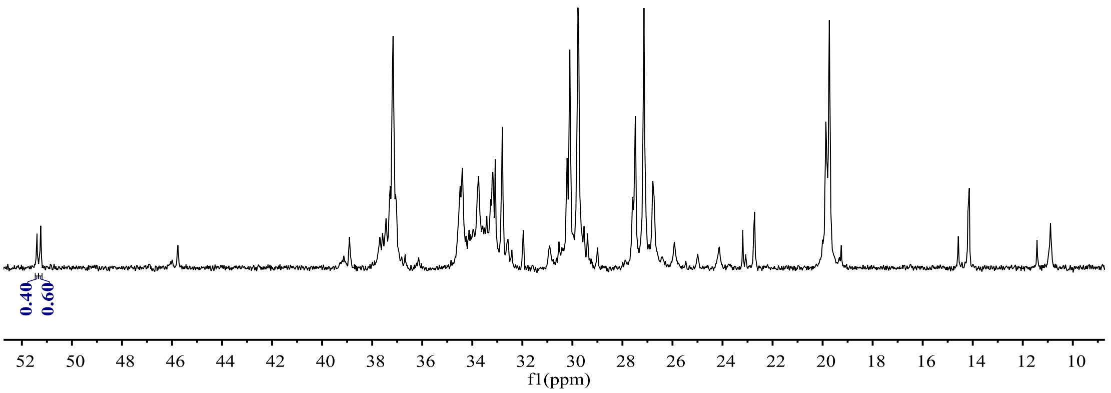

Figure S44. Partial enlargement of ^{13}C NMR spectrum (500 MHz, $CDCl_3$, 25 °C) of the copolymer generated by **Ipty/^{i}Pr-Pd** from table 5, entry 6.

3. GPC figures of (co)polymers

MW Averages

Mp: 1093738	Mn: 811951	Mv: 1319183	Mw: 1417359
Mz: 2295493	Mz+1: 3421968	PD: 1.7456	

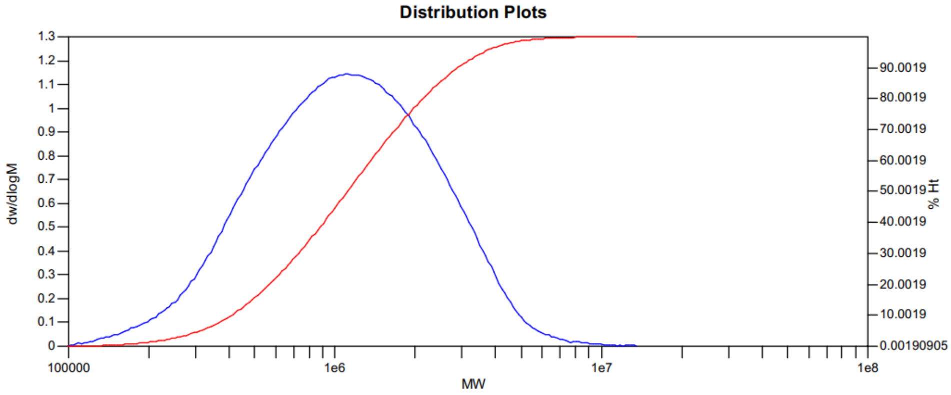

Figure S45. GPC trace of the polymer from table 1, entry 1.

MW Averages

Mp: 1261355	Mn: 920209	Mv: 1411597	Mw: 1503229
Mz: 2262177	Mz+1: 3036338	PD: 1.6336	

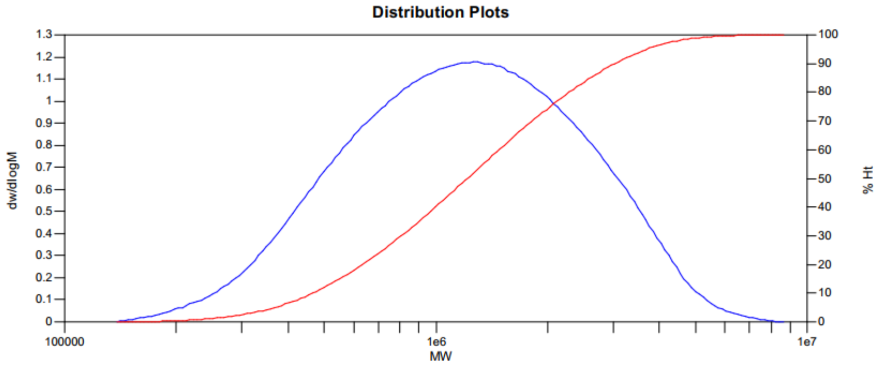

Figure S46. GPC trace of the polymer from table 1, entry 2.

MW Averages

Mp: 730234	Mn: 480774	Mv: 821541	Mw: 882787
Mz: 1417381	Mz+1: 2046055	PD: 1.8362	

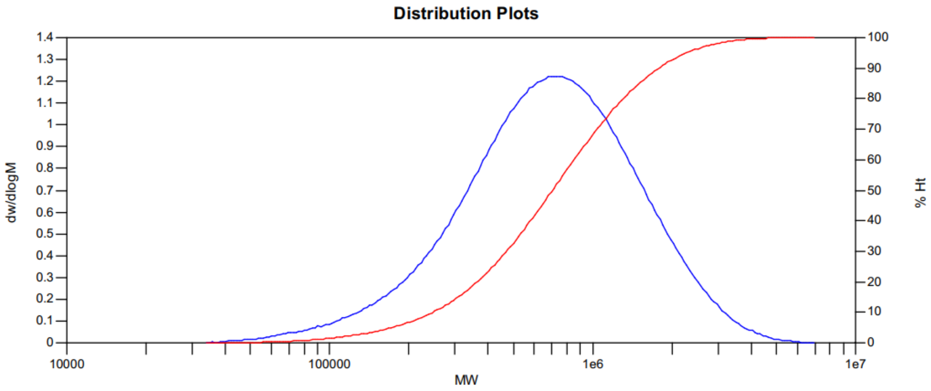

Figure S47. GPC trace of the polymer from table 1, entry 3.

MW Averages

Mp: 1717932 Mn: 1113145 Mv: 1730991 Mw: 1835682

Mz: 2697272 Mz+1: 3687949 PD: 1.6491

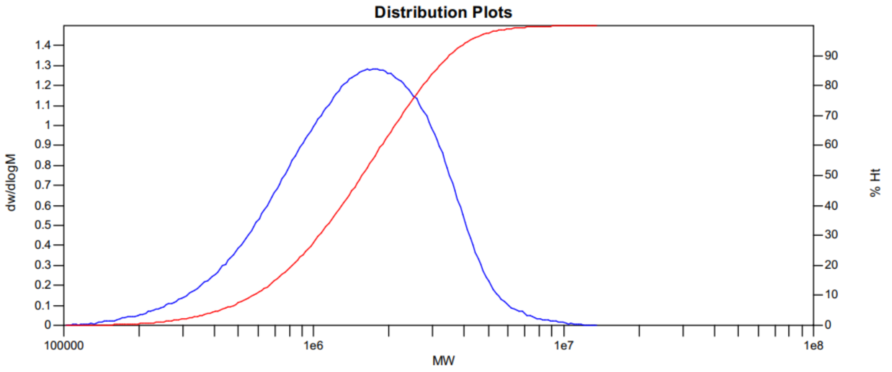

Figure S48. GPC trace of the polymer from table 2, entry 2.

MW Averages

Mp: 1018477 Mn: 655900 Mv: 1119558 Mw: 1202913

Mz: 1924737 Mz+1: 2766909 PD: 1.8340

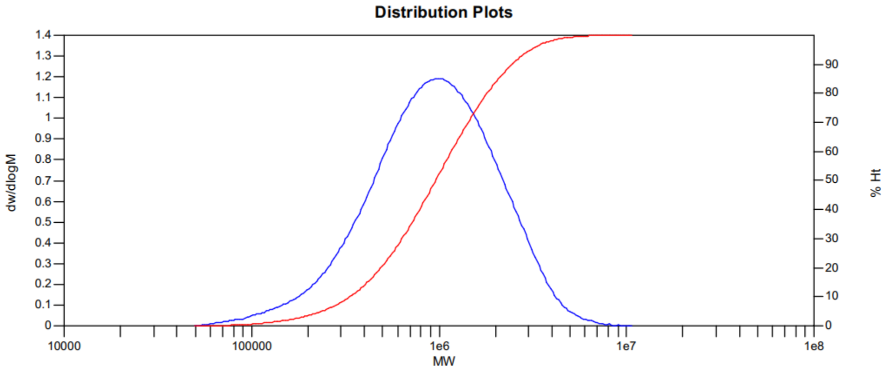

Figure S49. GPC trace of the polymer from table 2, entry 3.

MW Averages

Mp: 575778 Mn: 403543 Mv: 686516 Mw: 740445

Mz: 1236656 Mz+1: 1903006 PD: 1.8349

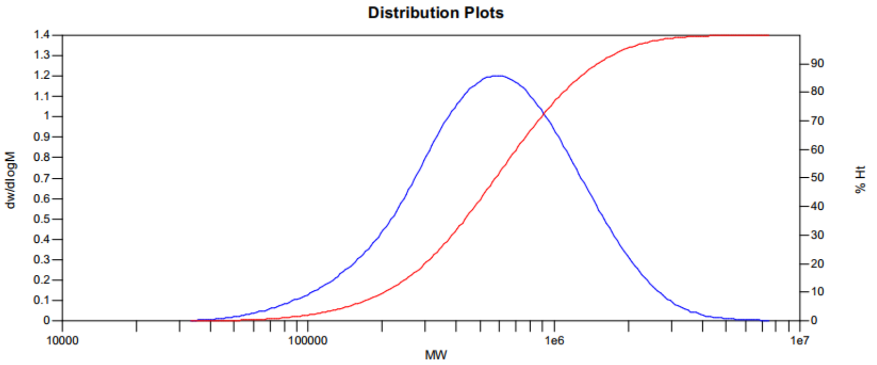

Figure S50. GPC trace of the polymer from table 2, entry 4.

MW Averages

Mp: 175478 Mn: 103642 Mv: 190150 Mw: 206074

Mz: 350278 Mz+1: 533672 PD: 1.9883

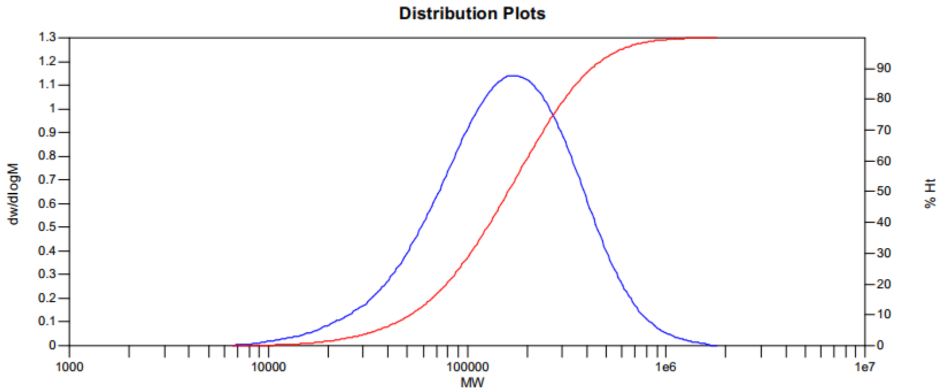

Figure S51. GPC trace of the polymer from table 2, entry 6.

MW Averages

Mp: 1844879 Mn: 962059 Mv: 1623332 Mw: 1730773

Mz: 2584133 Mz+1: 3464572 PD: 1.7990

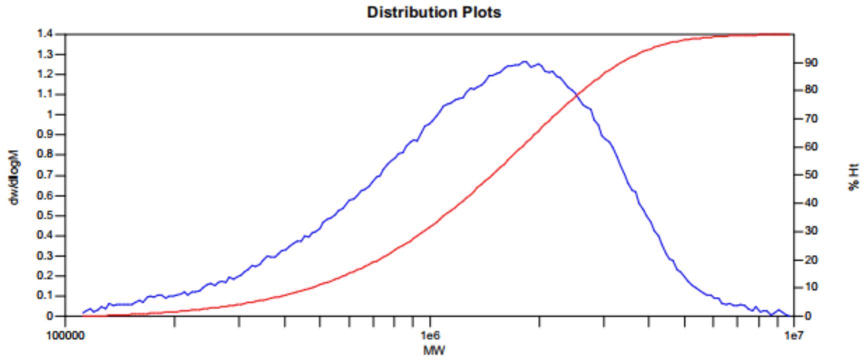

Figure S52. GPC trace of the polymer from table 2, entry 7.

MW Averages

Mp: 536159 Mn: 334936 Mv: 544933 Mw: 579659

Mz: 871936 Mz+1: 1229285 PD: 1.7307

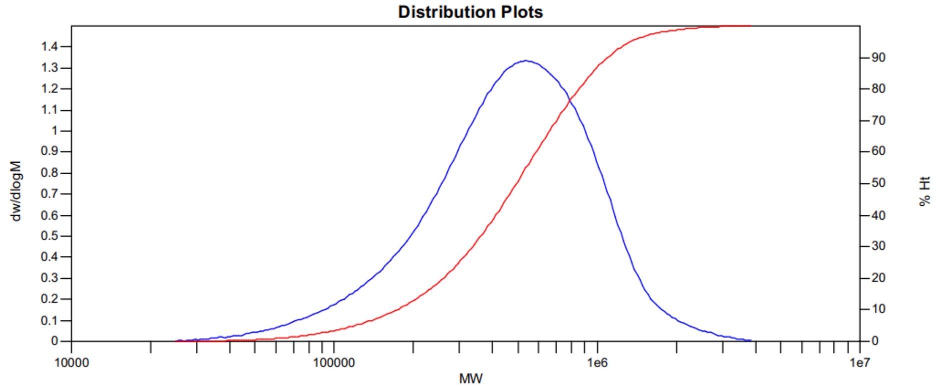

Figure S53. GPC trace of the polymer from table 2, entry 8.

MW Averages

Mp: 422753	Mn: 297887	Mv: 453066	Mw: 480250
Mz: 702790	Mz+1: 939452	PD: 1.6122	

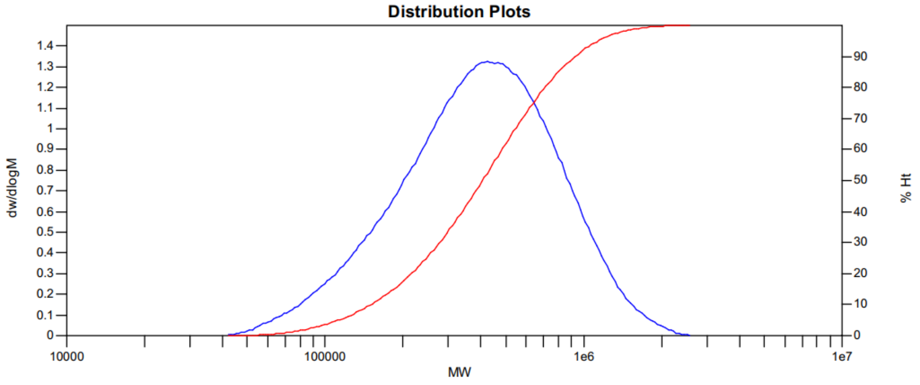

Figure S54. GPC trace of the polymer from table 2, entry 9.

MW Averages

Mp: 432920	Mn: 257237	Mv: 417517	Mw: 442776
Mz: 653940	Mz+1: 916944	PD: 1.7213	

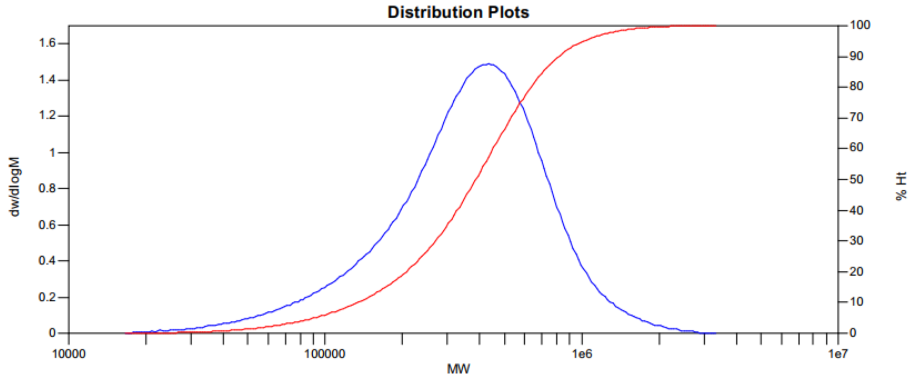

Figure S55. GPC trace of the copolymer from table 3, entry 1.

MW Averages

Mp: 349560	Mn: 219994	Mv: 376104	Mw: 403940
Mz: 647174	Mz+1: 944984	PD: 1.8361	

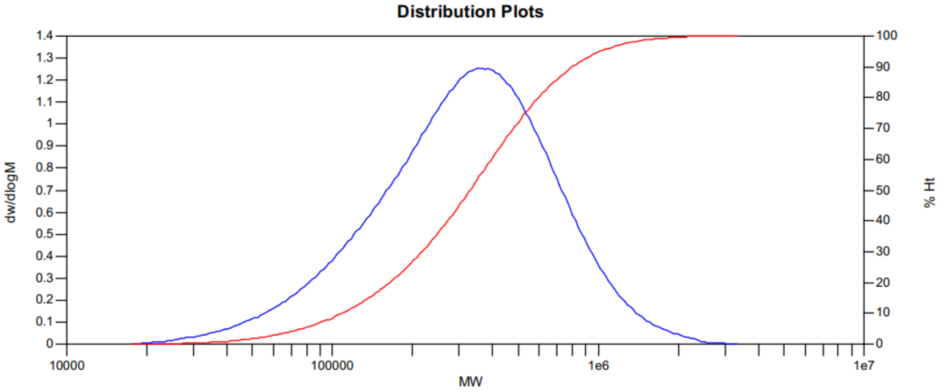

Figure S56. GPC trace of the copolymer from table 3, entry 2.

MW Averages

Mp: 163403	Mn: 104874	Mv: 148237	Mw: 155457
Mz: 216805	Mz+1: 299088	PD: 1.4823	

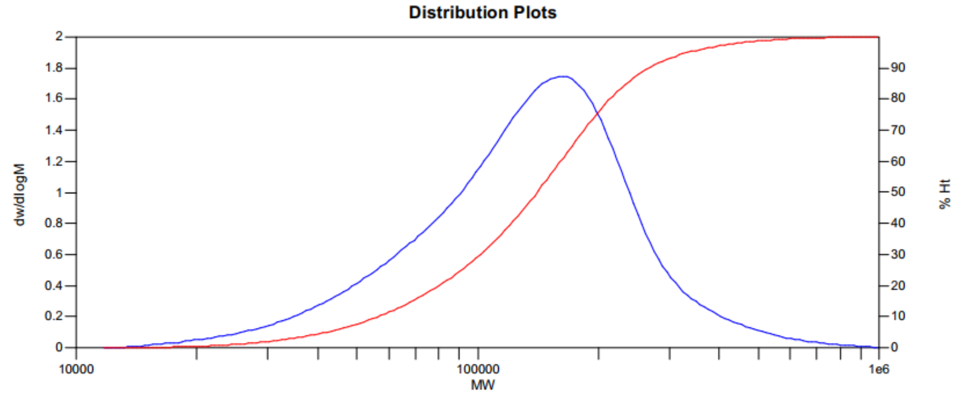

Figure S57. GPC trace of the polymer from table 4, entry 1.

MW Averages

Mp: 148586	Mn: 97013	Mv: 145357	Mw: 152011
Mz: 207169	Mz+1: 282952	PD: 1.5669	

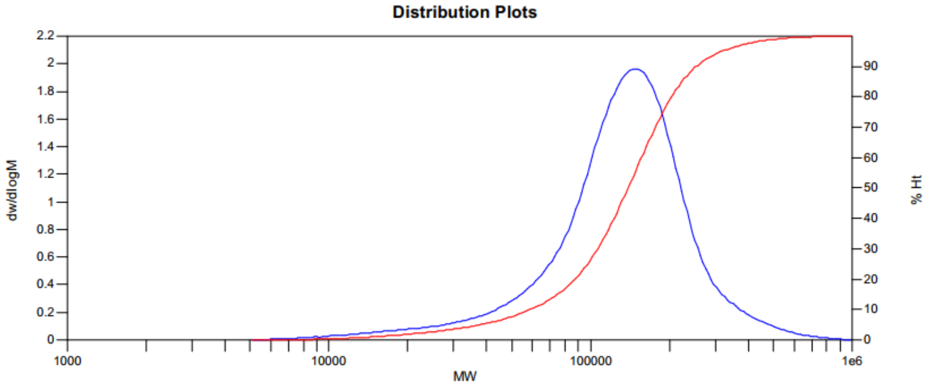

Figure S58. GPC trace of the polymer from table 4, entry 2.

MW Averages

Mp: 138361	Mn: 70957	Mv: 147704	Mw: 162106
Mz: 292654	Mz+1: 453010	PD: 2.2846	

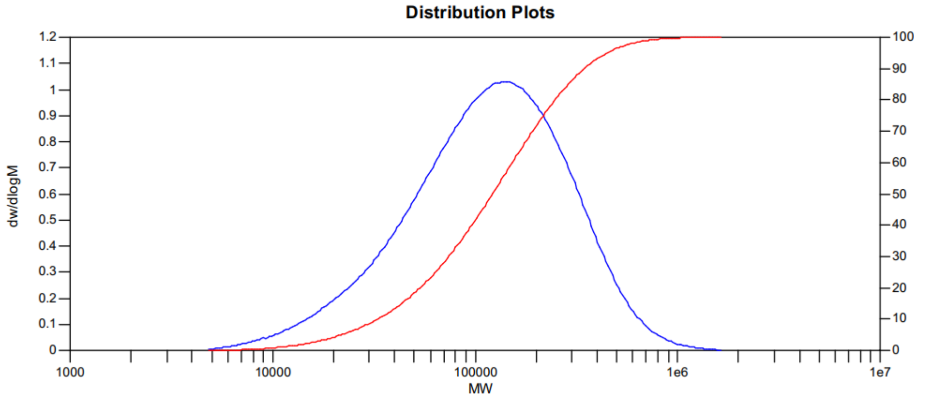

Figure S59. GPC trace of the polymer from table 4, entry 3.

MW Averages

Mp: 63158	Mn: 36897	Mv: 70738	Mw: 77270
Mz: 138995	Mz+1: 221398	PD: 2.0942	

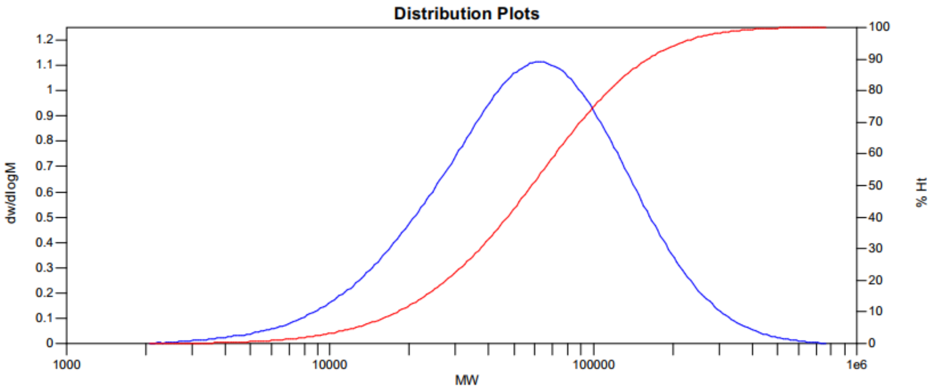

Figure S60. GPC trace of the polymer from table 4, entry 4.

MW Averages

Mp: 37443	Mn: 21775	Mv: 42280	Mw: 46206
Mz: 84411	Mz+1: 140788	PD: 2.1220	

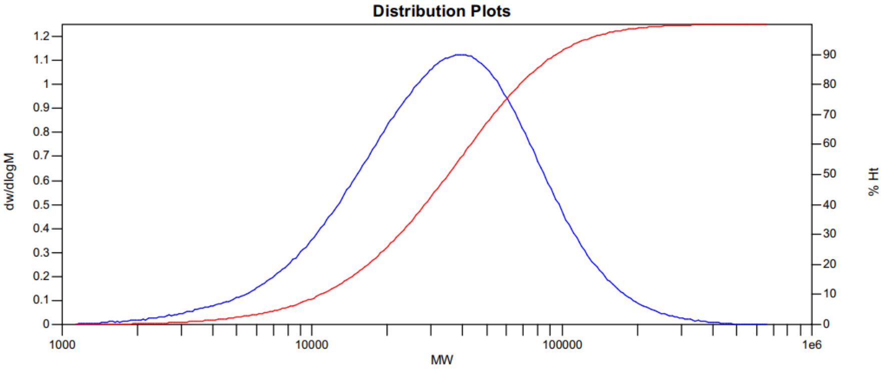

Figure S61. GPC trace of the polymer from table 4, entry 5.

MW Averages

Mp: 17924	Mn: 10834	Mv: 20090	Mw: 21794
Mz: 37145	Mz+1: 56137	PD: 2.0116	

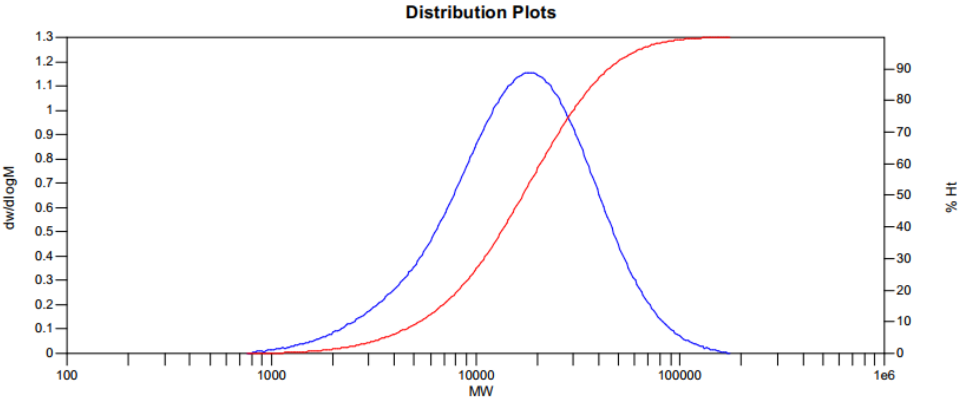

Figure S62. GPC trace of the polymer from table 4, entry 6.

MW Averages

Mp: 145096	Mn: 80332	Mv: 151397	Mw: 164446
Mz: 280975	Mz+1: 422995	PD: 2.0471	

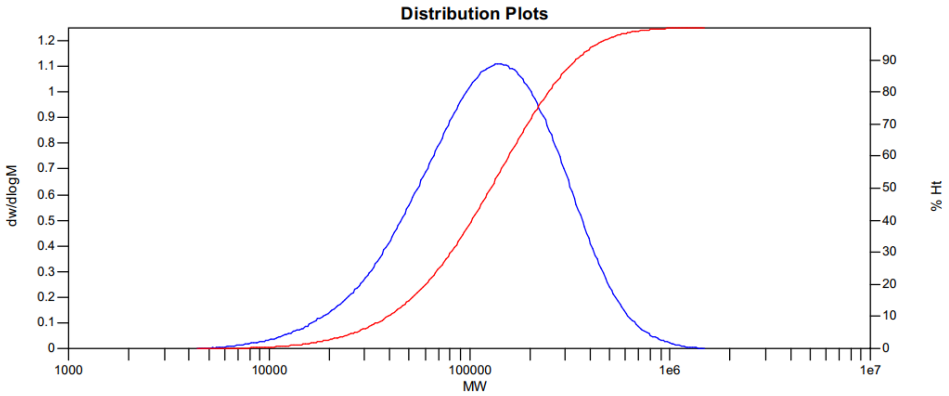

Figure S63. GPC trace of the polymer from table 4, entry 7.

MW Averages

Mp: 33248	Mn: 15823	Mv: 33357	Mw: 35944
Mz: 58484	Mz+1: 88976	PD: 2.2716	

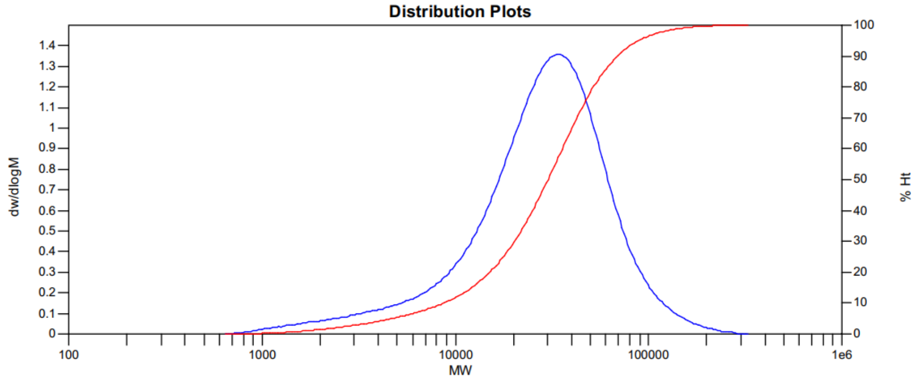

Figure S64. GPC trace of the copolymer from table 5, entry 1.

MW Averages

Mp: 60227	Mn: 40557	Mv: 62639	Mw: 66150
Mz: 96425	Mz+1: 137203	PD: 1.6310	

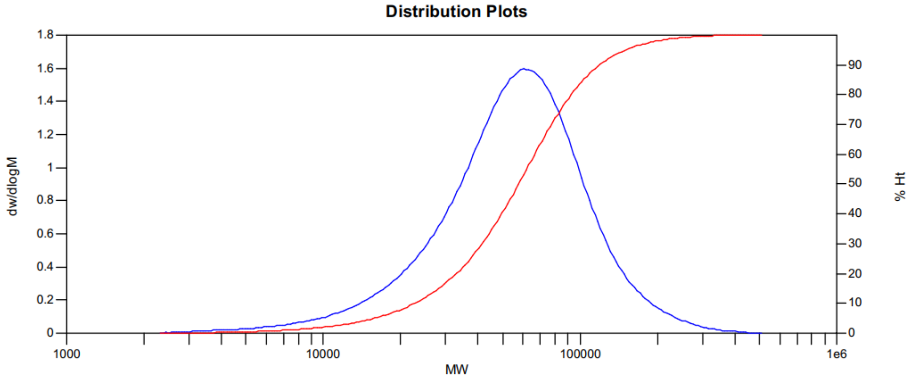

Figure S65. GPC trace of the copolymer from table 5, entry 2.

MW Averages

Mp: 63158	Mn: 38941	Mv: 68455	Mw: 73555
Mz: 119033	Mz+1: 178384	PD: 1.8889	

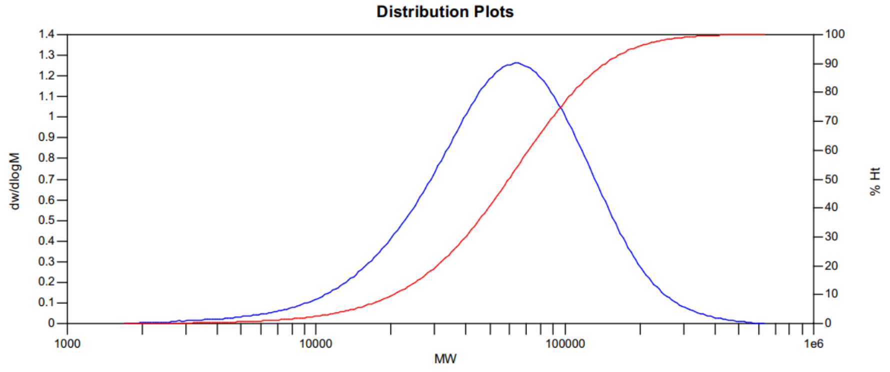

Figure S66. GPC trace of the copolymer from table 5, entry 3.

MW Averages

Mp: 32468	Mn: 17881	Mv: 34981	Mw: 37978
Mz: 65381	Mz+1: 101817	PD: 2.1239	

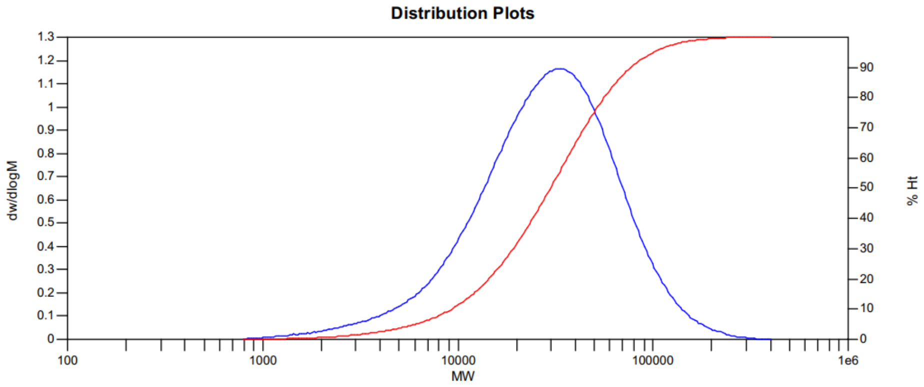

Figure S67. GPC trace of the copolymer from table 5, entry 4.

4. DSC figures of (co)polymers

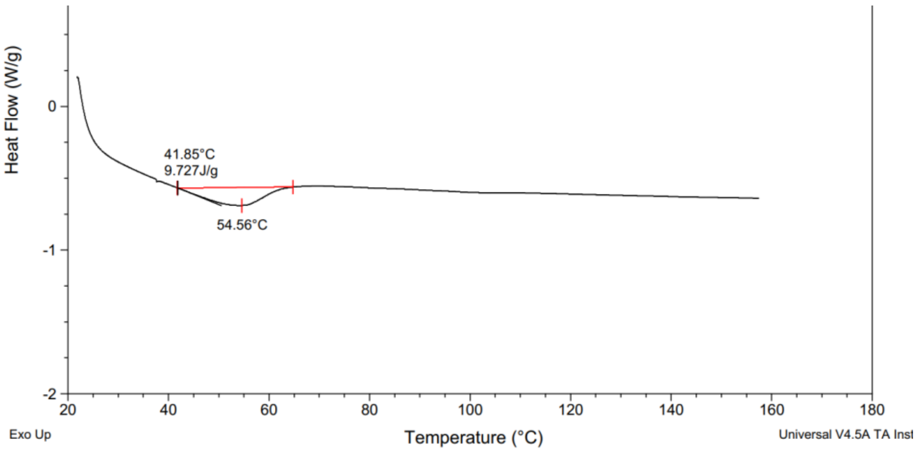

Figure S68. DSC data of the polymer from table 1, entry 1.

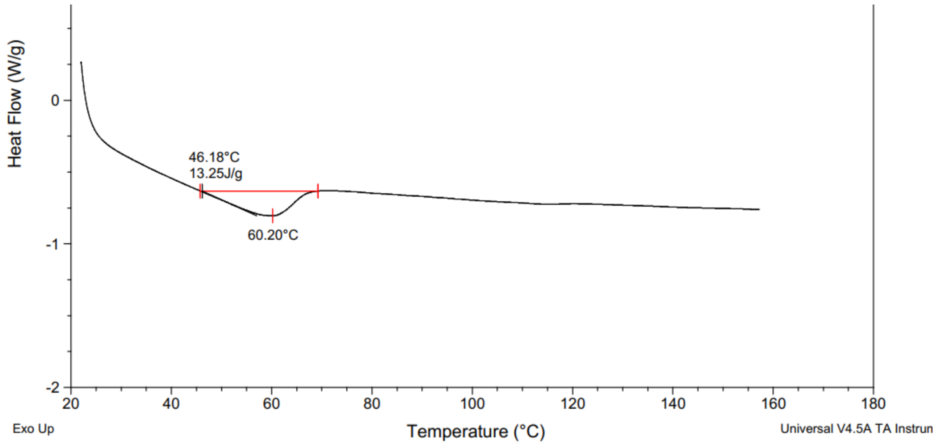

Figure S69. DSC data of the polymer from table 1, entry 2.

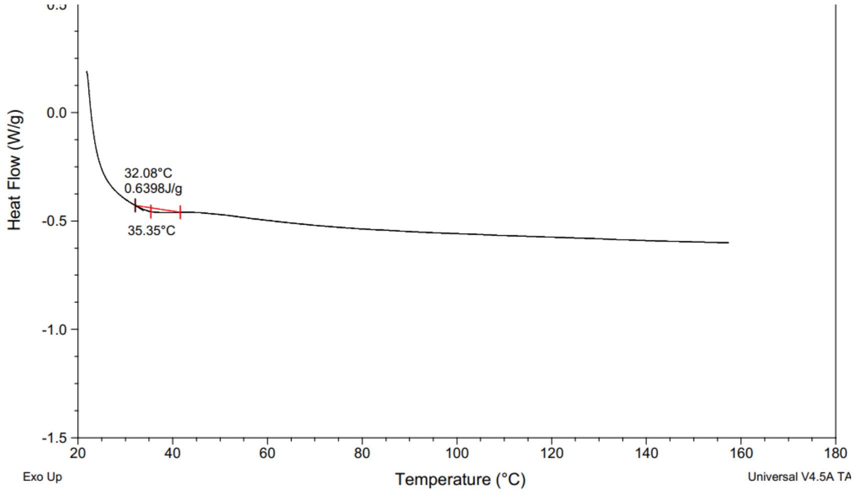

Figure S70. DSC data of the polymer from table 1, entry 3.

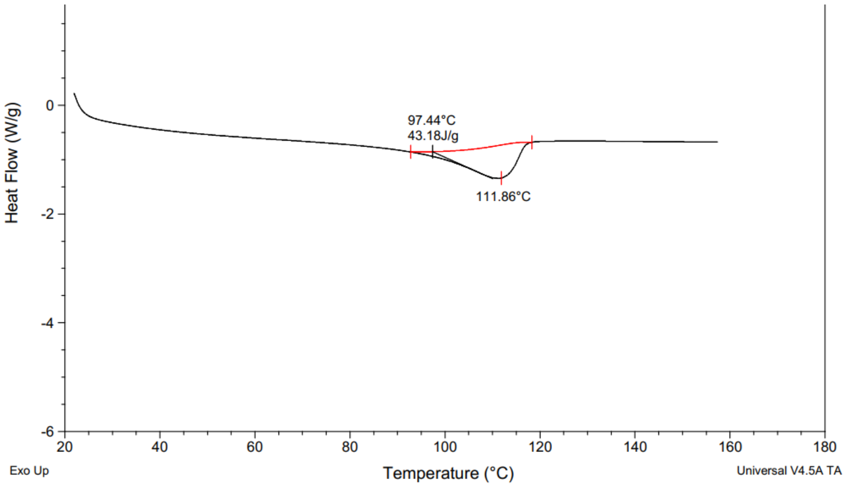

Figure S71. DSC data of the polymer from table 2, entry 1.

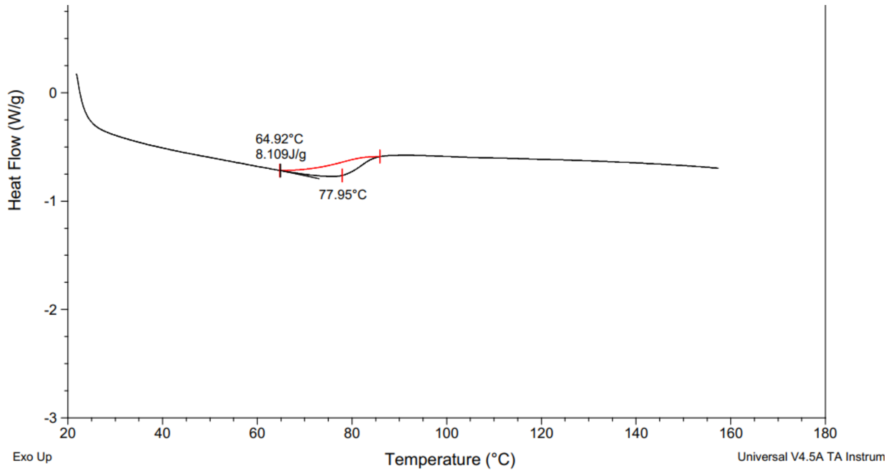

Figure S72. DSC data of the polymer from table 2, entry 2.

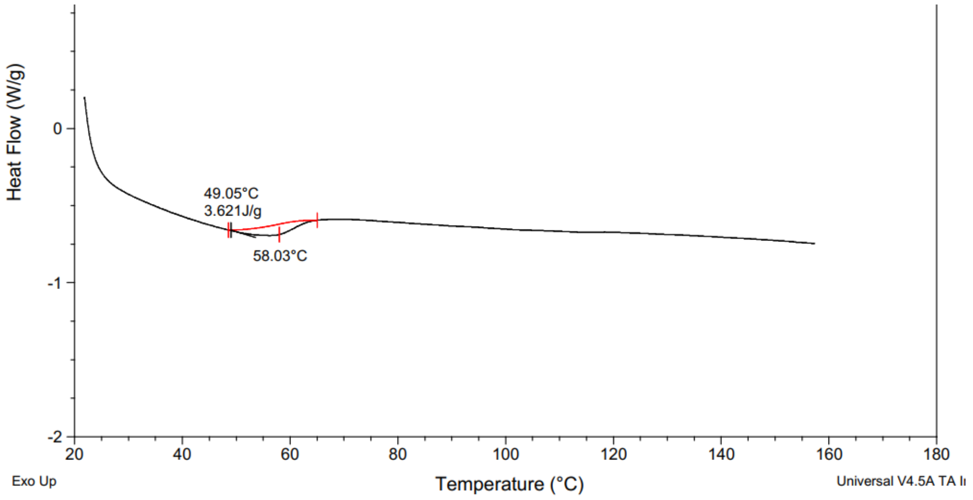

Figure S73. DSC data of the polymer from table 2, entry 3.

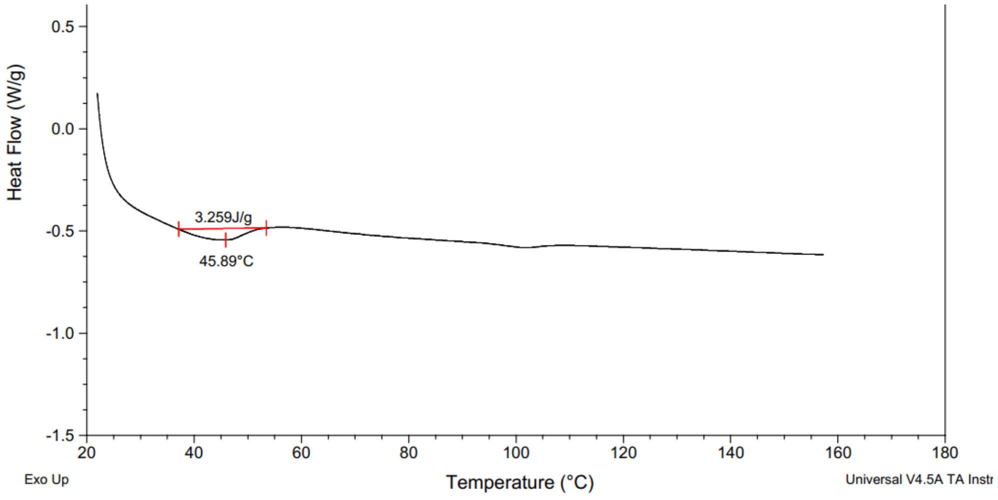

Figure S74. DSC data of the polymer from table 2, entry 4.

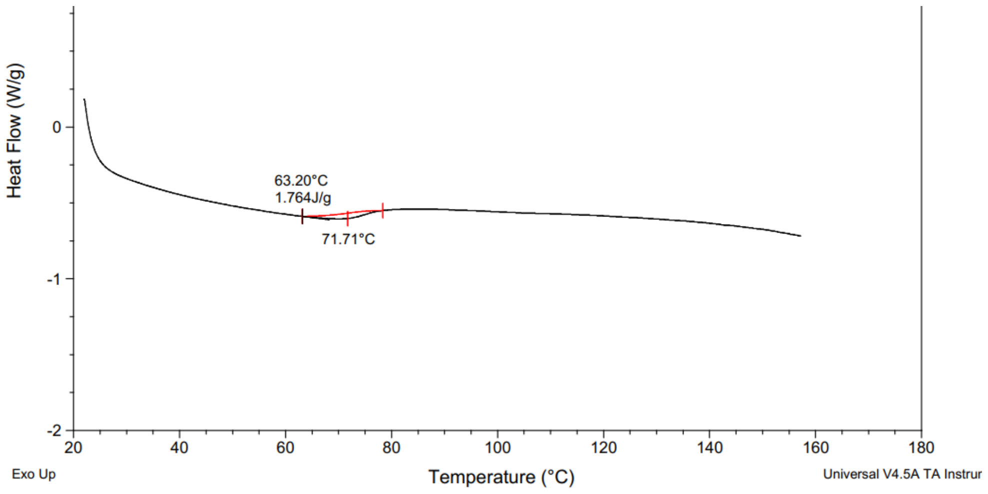

Figure S75. DSC data of the polymer from table 2, entry 7.

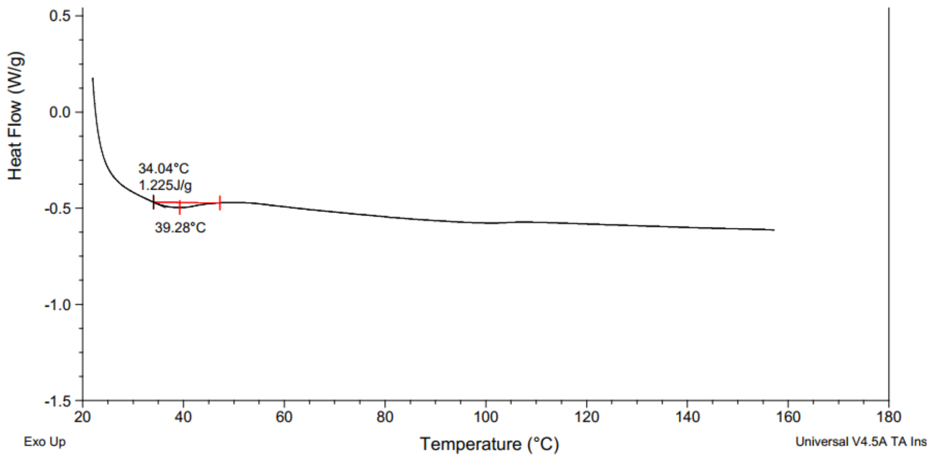

Figure S76. DSC data of the polymer from table 2, entry 8.

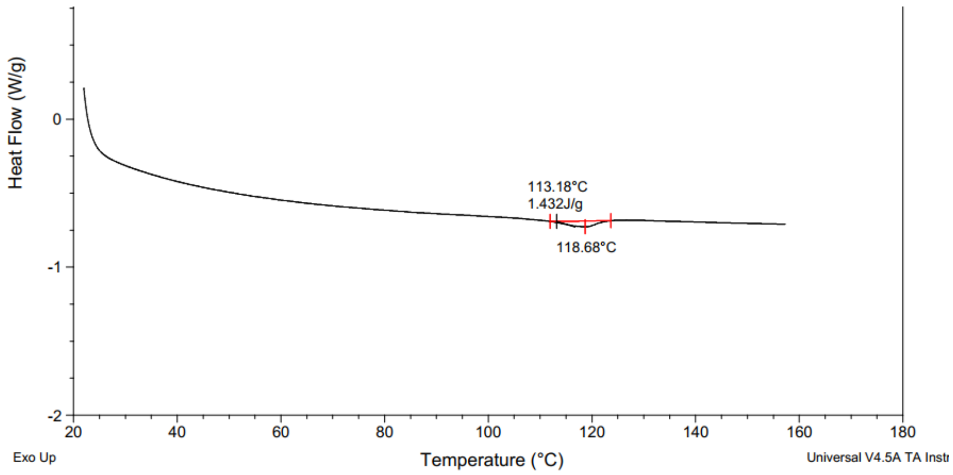

Figure S77. DSC data of the polymer from table 2, entry 9.

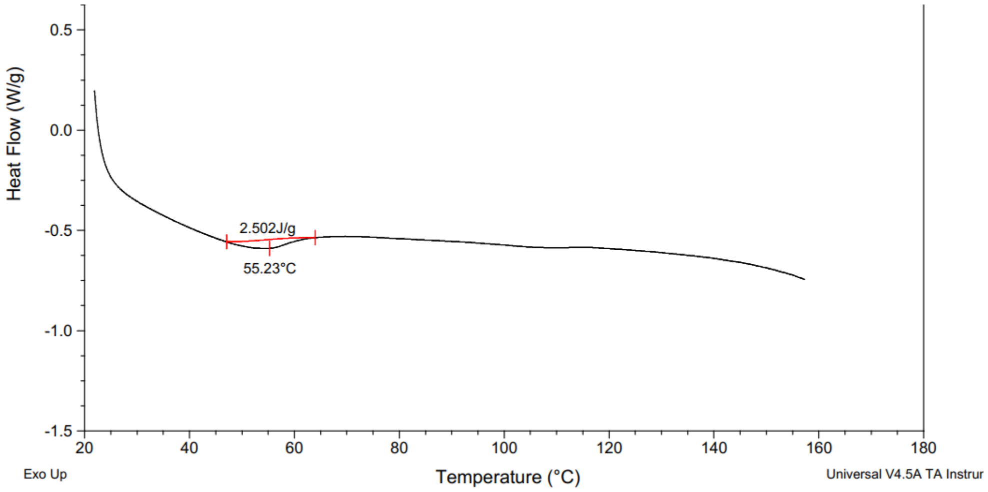

Figure S78. DSC data of the polymer from table 3, entry 2.

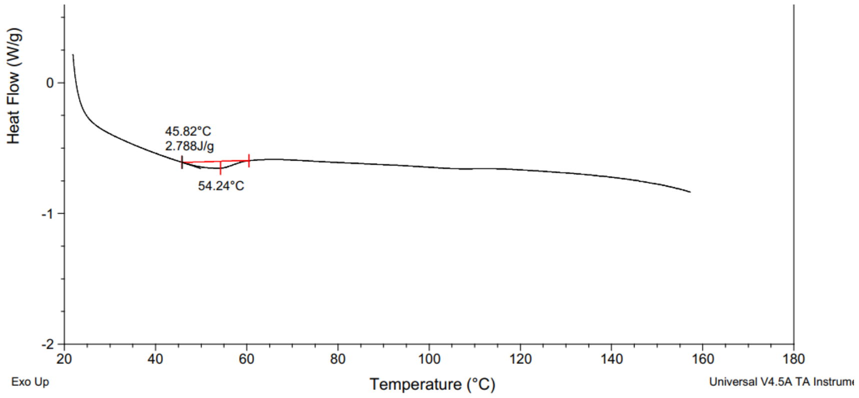

Figure S79. DSC data of the polymer from table 3, entry 3.

5. Crystallographic data

Table S1. Crystallographic data for Ipty/^{i}Pr-Ni and Ipty/^{i}Pr-Pd.

	**Ipty/^{i}Pr-Ni**	**Ipty/^{i}Pr-Pd CH_2Cl_2**
Formula	$C_{63}H_{50}Br_2N_2NiO$	$C_{65}H_{55}Cl_3N_2OPd$
Formula weight	1069.58	1092.86
Crystal dimensions (mm^3)	0.30 × 0.28 × 0.26	0.30 × 0.28 × 0.02
Crystal system	orthorhombic	orthorhombic
Space group	Pna21	Pna21
a (Å)	16.5290(17)	16.5938(6)
b (Å)	16.2669(17)	16.1101(6)
c (Å)	23.061(2)	23.2371(9)
α (°)	90	90
β (°)	90	90
γ (°)	90	90
Volume (Å^3)	6200.5(11)	6211.9(4)
Z	4	4
T (K)	293(2)	293(2)

D_{calcd} (g cm^{-3})	1.146	1.169
μ (mm^{-1})	1.638	3.892
F (000)	2192	2256
No. of rflns. collected	41834	27955
No. of indep. rflns. /R_{int}	10670 / 0.1016	8644 / 0.0367
No. of obsd. rflns. [$I_0 > 2\sigma(I_0)$]	7073	7561
Data / restraints / parameters	10670 / 1 / 628	8644 / 1 / 651
R_1 / wR_2 [$I_0 > 2\sigma(I_0)$]	0.0734 / 0.1962	0.0397 / 0.1049
R_1 / wR_2 (all data)	0.1217 / 0.1665	0.0491 / 0.0995
GOF (on F^2)	1.018	1.049
Largest diff. peak and hole (e Å^{-3})	1.120 / −1.159	0.489 / −0.510
CCDC No.	2214840	2214844
